# Current prevalence of chronic hepatitis B and C virus infection in the general population, blood donors and pregnant women in the EU/EEA: a systematic review

**DOI:** 10.1017/S0950268817001947

**Published:** 2017-09-11

**Authors:** S. H. I. HOFSTRAAT, A. M. FALLA, E. F. DUFFELL, S. J. M. HAHNÉ, A. J. AMATO-GAUCI, I. K. VELDHUIJZEN, L. TAVOSCHI

**Affiliations:** 1 Centre for Infectious Disease Control, National Institute for Public Health and the Environment (RIVM), Bilthoven, The Netherlands; 2 Department of Infectious Disease Control, Municipal Public Health Service Rotterdam-Rijnmond, Rotterdam, The Netherlands; 3 Department of Public Health, Erasmus MC, University Medical Centre Rotterdam, Rotterdam, The Netherlands; 4 European Centre for Disease Prevention and Control, Stockholm, Sweden

**Keywords:** Hepatitis B, hepatitis C, prevalence, systematic review

## Abstract

This systematic review aimed at estimating chronic hepatitis B (HBV) and C virus (HCV) prevalence in the European Union (EU) and Economic Area (EEA) countries in the general population, blood donors and pregnant women. We searched PubMed^©^, Embase^©^ and Cochrane Library databases for reports on HBV and HCV prevalence in the general population and pregnant women in EU/EEA countries published between 2005 and 2015. Council of Europe data were used for HBV and HCV blood donor prevalence. HBV general population estimates were available for 13 countries, ranging from 0·1% to 4·4%. HCV general population estimates were available for 13 countries, ranging from 0·1% to 5·9%. Based on general population and blood donor estimates, the overall HBV prevalence in the EU/EEA is estimated to be 0·9% (95% CI 0·7–1·2), corresponding to almost 4·7 million HBsAg-positive cases; and the overall HCV prevalence to be 1·1% (95% CI 0·9–1·4), equalling 5·6 million anti-HCV-positive cases. We found wide variation in HCV and HBV prevalence across EU/EEA countries for which estimates were available, as well as variability between groups often considered a proxy for the general population. Prevalence estimates are essential to inform policymaking and public health practice. Comparing to other regions globally, HBV and HCV prevalence in the EU/EEA is low.

## INTRODUCTION

Both hepatitis B (HBV) and C virus (HCV) affect the liver and can cause acute and chronic hepatitis. People with chronic HBV or HCV infection may transmit the virus to others and are at risk of developing serious liver disease such as cirrhosis or hepatocellular cancer (HCC) [1, 2]. Transmission of HBV and HCV can occur via sexual or blood–blood contact, or vertically (mother-to-child) [3, 4]. In Europe, the high-risk groups for acquisition of HBV include men who have sex with men (MSM) and people who inject drugs (PWID). The key risk groups for HCV include PWID, people in prison and MSM.

The risk of developing chronic HBV infection depends on the age at which people are infected, with 90% of infants infected at birth developing chronic infection, compared with 1–10% of those infected at an older age or as adults [5, 6]. Globally 248 million people were estimated to be chronically infected with HBV in 2010 [7]. Approximately 780 000 people die each year from HBV infection, mostly from chronic HBV infection-related sequelae such as cirrhosis and HCC [8].

Initial infection with HCV is often asymptomatic or mild (70–90% of cases); however, the majority of those infected with the virus (70–80%) develop chronic infection and, over a period of 20–30 years, 10–20% on average will develop cirrhosis and 1–5% will develop liver cancer [2]. An estimated 184 million people globally have chronic HCV infection [9] and 350 000–500 000 deaths are attributable each year to HCV-related liver diseases [8].

In 2011, the European Centre for Disease Prevention and Control (ECDC) started enhanced European Union (EU)-wide surveillance for HBV and HCV, based on annual data collection from EU and Economic Area (EEA) Member States (MS). In 2014, 22442 newly diagnosed HBV infection cases were reported from 30 MS, a rate of 4·2 cases/100 000 population [10]. In the same year, 35 321 newly diagnosed HCV infection cases were reported from 28 MS, a crude rate of 8·8 cases/100 000 population in the EU/EEA [11]. However, because HBV and HCV infections are typically asymptomatic until advanced liver disease develops [1, 2], notification data are known to be incomplete and reflect national screening and testing practices rather than the real number of infections. Supplementary information in the form of reliable and timely prevalence data is therefore important to describe the current burden of disease in the EU/EEA more accurately.

The recent development of more effective HBV and HCV treatment means that elimination of chronic viral hepatitis in Europe is now a possibility [12, 13]. However, 65–90% of infected people remain unaware of their infection and models predict that associated mortality will continue to increase as the current infected population ages [12, 14, 15]. Achieving potential population health gains through treatment will require significant expansion of screening and treatment among the most affected populations. Robust strategic information will be of even more relevance in view of the recently approved WHO viral hepatitis global health sector strategy, the corresponding European regional action plan, and its monitoring needs [16, 17].

We updated a previous systematic review undertaken by ECDC in 2009 [18] with the aim to assess any changes and estimate the current prevalence of chronic HBV and HCV infection in EU/EEA countries in the general population, blood donors and pregnant women. As a secondary goal, we reviewed the availability, quality and geographical coverage of HBV and HCV prevalence data in the region in view of designing and monitoring future prevention and control initiatives.

## METHODS

### Search strategy and selection criteria

Original research articles were retrieved from PubMed®, Embase® and Cochrane Library databases in March 2015. The search strategies (Supplementary Fig. S1) combined controlled (MeSH/Emtree terms) and natural vocabulary (keywords) to define disease-related (HBV/HCV infection), outcome-related (prevalence) and geographic-related (EU/EEA) search parameters. The search was limited to records published from 1 January 2005 to 12 March 2015. Articles in all EU/EEA languages were included. The results of the search were shared with ECDC National Focal Points (NFP) for viral hepatitis in all 31 EU/EEA MS in May 2015 for review and to validate the list of included references for their country.

Inclusion and exclusion criteria (Supplementary Table S2) encompassed time-related criteria including publishing date (2005 or later); sampling timeframe (data collection ending after 2000 or beginning from 2000 onwards); geographical coverage (EU/EEA MS and/or any of their regions/districts) and disease-specific markers (HBsAg/anti-HCV (and DNA/RNA) prevalence). Only studies reporting original data were included, although reference lists of relevant reviews were consulted for any original articles not captured by the literature search. Articles reporting prevalence in the general population or pregnant women with a sample size of <100 participants were excluded. Articles reporting only self-reported HBsAg/anti-HCV prevalence were also excluded.

To ensure consistent application of the inclusion criteria, two reviewers (SHIH and AMF) independently reviewed the title and abstract of the same random selection of retrieved articles (5%). The inclusion criteria were further refined and a second round of reviewing was conducted to ensure consistent application (>95% agreement) of the criteria. Following this, title and abstract screening for all articles continued independently using Endnote. The full texts of all publications included after title/abstract screening were assessed for relevance by members of the research team where language comprehension existed (articles in English, Dutch, French, Italian and German) or by ECDC reviewers (other EU/EEA languages). In case of uncertainty about in- or exclusion, the two reviewers consulted each other and cases of disagreement were resolved by consultation with a third team member (IKV).

### Definitions

Chronic HBV and HCV were defined as the presence of HBsAg and anti-HCV in serum, saliva or dry blood spot samples, respectively. The general population was defined as people (all ages or adults only) living in a defined geographical area; patients attending community/primary health care settings; and workforce/specific professional groups (e.g. workplace screening) but not healthcare workers. Pregnant women were defined as those women undergoing antenatal care screening, and blood donors were defined as first-time blood donors (Supplementary Table S3).

### Data extraction and quality assessment

Data extraction using a pre-defined set of variables (Supplementary Table S4) was performed simultaneously with full-text screening. The unit for data extraction was ‘study’, defined as a prevalence data report on HBsAg or anti-HCV for a defined population group, in a defined country, over a discrete period; one article may therefore include more than one study. Studies published in more than one article were extracted only once and the article with most details about the study used as the reference.

Each included study was evaluated for risk of selection bias using a framework developed *ad hoc* by the research team. Separate assessment frameworks were developed for the general population and pregnant women to account for differences in possible sources of selection bias. For general population studies, the domains age, gender, sampling method and response rate, and geographical coverage were considered as possible sources of selection bias (Supplementary Table S5). For pregnant women studies, potential selection bias sources included sampling method and geographical coverage (Supplementary Table S6). Points were awarded in each domain for representativeness or lower risk of bias, and a total score was calculated by summing the values in each domain. This resulted in a score between zero and six for the general population studies and between zero and three for the pregnant women studies. We refer to the total score as study quality score, since a higher score indicates a lower risk of bias. A general population estimate was considered of high quality when it reached a study quality score ⩾4. A high-quality estimate of prevalence in pregnant women was defined as a study quality score ⩾2.

### Data analysis

This review reports HBsAg and anti-HCV prevalence, rather than a viraemic marker of HCV chronic infection, since information on HCV RNA and HBV DNA prevalence was reported in too few studies to conduct an analysis.

National weighted or standardized (e.g. for age and/or sex distribution) prevalence estimates, if available (Czech Republic and Belgium for HBV), were preferred over unweighted or crude estimates. Crude estimates for the general population with the highest quality (score ⩾4) were pooled at country level, where available, by summing cases and sample size. Ninety-five per cent confidence intervals (95% CI) were then calculated using the Fisher's exact method in Microsoft Excel^©^. General population estimates were reported separately for adults and children where available. All higher quality estimates of HBsAg and anti-HCV prevalence (score ⩾4) retrieved for each country for the general population are presented in forest plots (Microsoft Excel^©^). Higher quality prevalence estimates from pregnant women studies (score ⩾2) were also pooled where possible (using the methodology as described above) and separate forest plots prepared for HBV and HCV. Prevalence maps of Europe for pooled or single higher quality estimates were created using the ECDC Mapping and Multi-Layer Analysis (EMMA) tool [19].

### Blood donor data

To assess the HBV and HCV prevalence among blood donors, data from 2014 collected by the Council of Europe were used [20]. The Council of Europe collects comprehensive national data on blood donors. For countries with no data in the 2014 Council of Europe report, the most recent data from previous Council of Europe reports were used. No risk of bias assessment was performed for data on blood donors, as no data were available other than the number of first-time blood donors and the number of infections. When data on number of cases were available, we calculated 95% CI using the Fisher's exact method.

### The burden of chronic hepatitis B and C in the EU/EEA

In order to estimate the current burden of chronic HBV and HCV in the EU/EEA, an algorithm combining general population and blood donor data was applied. If a pooled or single higher quality general population prevalence crude estimate was available for a country, this was used to determine the HBV and HCV prevalence in that country; if a higher quality estimate was not available, lower quality general population crude estimates were used (these were pooled when possible); if no general population estimates were available, prevalence data from blood donors were used. To determine the total number of chronic HBV and HCV cases in each country, total population size (based on Eurostat 2014 data) for each country was multiplied by the estimated HBV and HCV prevalence in each country.

## RESULTS

**Fig. 1.**
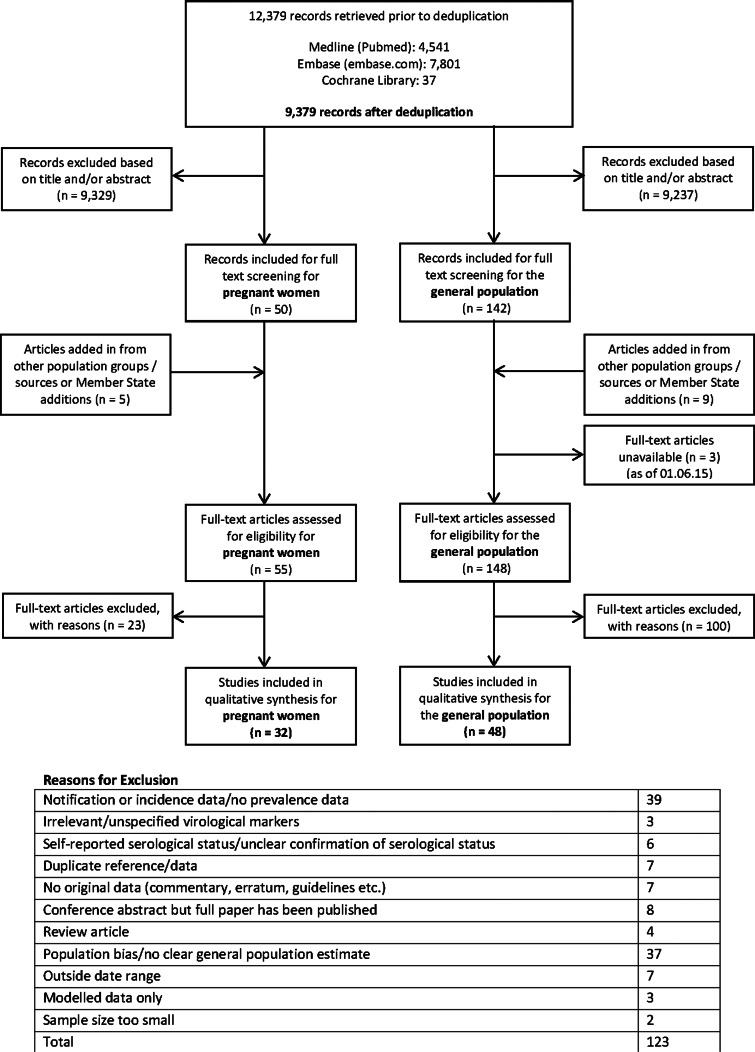
Flow chart of study selection for the general population and pregnant women; EU/EEA countries, 2005–2015.

The literature search retrieved 9379 citations. After title/abstract screening, 142 articles for the general population and 50 articles for pregnant women were included. Seventeen MS validated the search results and/or provided additional references, adding nine additional citations for the general population and five for pregnant women. While all 55 full texts were available for pregnant women, three general population articles could not be retrieved. Following full-text screening, 48 articles for the general population and 32 articles for pregnant women were finally included (Fig. 1).

### General population

From the 48 articles included, 53 HBsAg prevalence estimates and 45 anti-HCV estimates were extracted. For HBV, multiple estimates were available for 13 of 15 countries covered, with the most estimates (10) available for Italy (Supplementary Table S7). For HCV, more than one estimate was available for nine countries of 16 countries covered, with most estimates (14) again available for Italy (Supplementary Table S8).

**Fig. 2.**
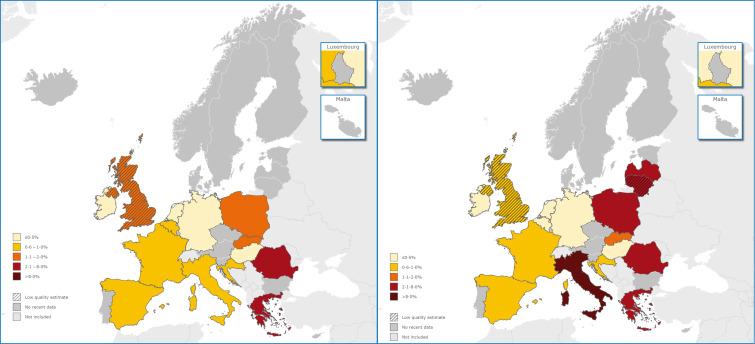
HBsAg prevalence (left) and anti-HCV prevalence (right) in the adult general population in the EU/EEA, based on studies published between 2005 and 2015.

**Fig. 3.**
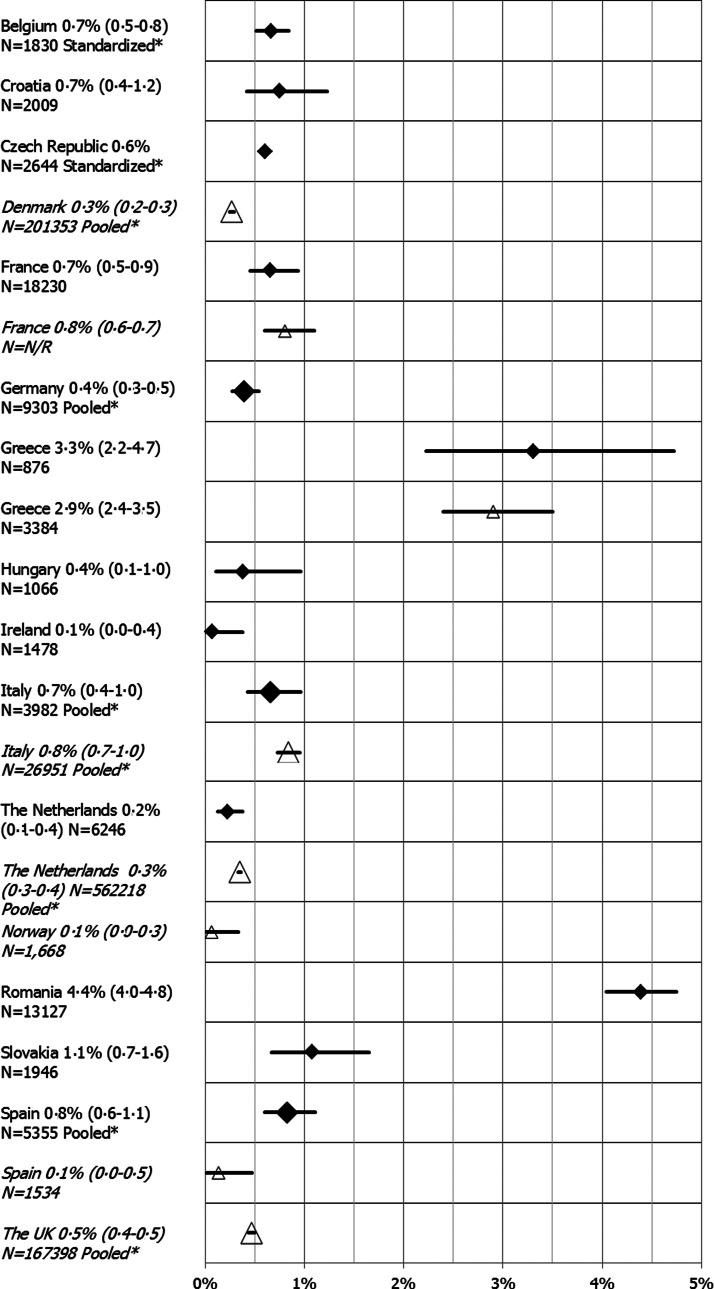
HBsAg prevalence estimates from studies with a lower risk of bias for the general population (study quality score ⩾4) and for pregnant women (study quality score ⩾2), in the EU/EEA, 2005–2015 (legend: country, prevalence estimate (95% CI) and sample size (N), general population estimates represented by diamond data points, pregnant women estimates represented in italics with triangle data points). *Standardized estimates were used for Belgium and Czech Republic. *Pooled estimates were used for Germany, Italy and Spain for the general population and for Denmark, Italy, the Netherlands and the UK for pregnant women.

From the 53 prevalence HBsAg estimates, 18 estimates in 13 countries (Belgium, Croatia, Czech Republic, France, Germany, Greece, Hungary, Ireland, Italy, the Netherlands, Romania, Slovakia and Spain) were considered to be of higher quality (score ⩾4, Supplementary Table S9). These data are presented in Figures 2 and 3. For Germany, Italy and Spain, multiple higher quality estimates were available and used to calculate a pooled estimate. The HBsAg prevalence in the general population ranged from 0·1% in Ireland [21] to 4·4% in Romania [22] (Fig. 3). Eleven of the 13 estimates were around or below 1%. Several higher quality prevalence estimates were available for Italy which, when pooled, resulted in an estimated HBV prevalence of 0·7%. There is, however, wide heterogeneity among these single study prevalence estimates from Italy, ranging from 0·5% (Apuglia, Southern Italy [23]) to 5·8% (Bergamo, Northern Italy [24]).

**Fig. 4.**
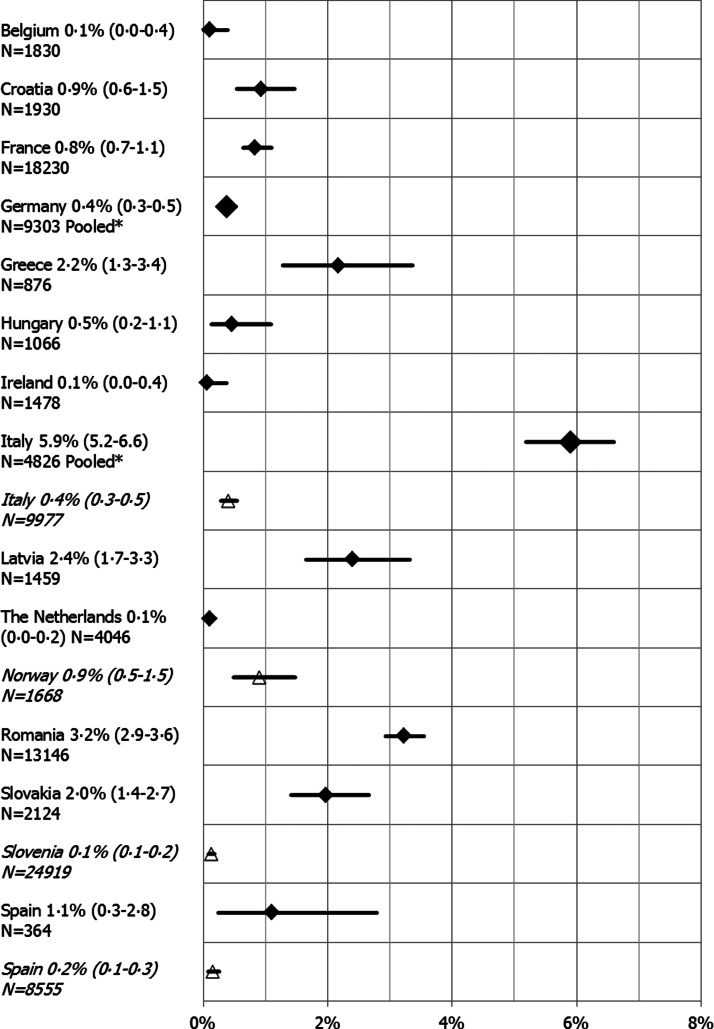
Anti-HCV prevalence estimates from studies with a lower risk of bias for the general population (study quality score ⩾4) and for pregnant women (study quality score ⩾2), in the EU/EEA, 2005–2015 (legend: country, prevalence estimate (95% CI) and sample size (N), general population estimates represented by diamond data points, pregnant women estimates in italics with triangle data points). *Pooled estimates were used for Germany and Italy.

Of the 45 anti-HCV prevalence estimates, 19 higher quality (score ⩾4, Supplementary Table S9) prevalence estimates from 13 countries (Belgium, Croatia, France, Germany, Greece, Hungary, Ireland, Italy, Latvia, the Netherlands, Romania, Slovakia and Spain) were available. These data are presented in Figures 2 and 4. Multiple higher quality estimates were available for a pooled estimate in Germany and Italy. The anti-HCV prevalence in the general population ranged from 0·1% in Belgium [25], Ireland [21] and the Netherlands [26] to 5·9% in Italy (Fig. 4). Relatively high anti-HCV prevalence was also found in Romania (3·2%) [27], Greece (2·2%) [28], Latvia (2·4%) [29] and Slovakia (2·0%) [30]. The estimate for Greece, however, is based on a sample from the population of Crete [28]. Four estimates were available for Spain, of which only one was of higher quality and reported an anti-HCV prevalence of 1·1% [31]. The others ranged from 0·4% in Barcelona [32] and 0·6% in Murcia and Madrid [33] to 1·5% in multiple GP practices around Barcelona [32].

### Pregnant women

To estimate the prevalence in pregnant women, 27 HBsAg estimates from 11 countries (Supplementary Table S10) and 15 anti-HCV estimates from eight countries (Supplementary Table S11) were retrieved from 32 eligible studies. Multiple estimates were available for nine countries, with the highest number of estimates (six) retrieved for Greece. Pooled estimates were available for Denmark, Italy, the Netherlands and the United Kingdom. Higher quality estimates (score ⩾2, Supplementary Table S12) of HBsAg prevalence were available for seven countries, ranging from 0·1% in Norway [34] and Spain [35] to 0·8% in France [36] and Italy (Fig. 3). For the Netherlands, HBsAg prevalence in pregnant women increased slightly from 0·3% in 2006 [37] and 2007 [37] to 0·4% in 2008 [37].

Of the 15 HCV estimates for pregnant women, higher quality estimates (score ⩾2, Supplementary Table S12) were available for Slovenia, Spain, Italy and Norway, with prevalence ranging from 0·1% in Slovenia [38] to 0·9% in Norway [34] (Fig. 4). The estimate for Slovenia is pooled, calculated using data from 2003, 2009 and 2013, which indicates a slight decrease in anti-HCV prevalence from 0·2% in 2003 to 0·1% in 2009 and 2013 [38].

### First-time blood donors

The prevalence of HBsAg and anti-HCV in first-time blood donors was available for 30 countries (Table 1). For Latvia and Portugal, the absolute number of positive cases and first-time blood donors were unavailable, thus no 95% CI could be calculated. The prevalence of chronic HBV infection among first-time blood donors ranged from 0·0% in Finland and Luxembourg to 3·2% in Bulgaria. Most countries (18/31, 58%) had an HBsAg prevalence that was around or below 0·1%.

**Table 1.** Prevalence of HBsAg and anti-HCV in first-time blood donors, EU/EEA*

Country	Prevalence of HBsAg (95% CI)	Prevalence of anti-HCV (95% CI)	Council of Europe Report
Austria	0·099% (0·072–0·132)	0·039% (0·023–0·061)	2010
Belgium	0·077% (0·055–0·104)	0·039% (0·024–0·060)	2011
Bulgaria	3·224% (3·039–3·418)	0·342% (0·282–0·410)	2011
Croatia	0·233% (0·142–0·359)	0·140% (0·072–0·244)	2011
Republic of Cyprus	0·441% (0·270–0·681)	0·221% (0·106–0·405)	2008
Czech Republic	0·059% (0·040–0·085)	0·216% (0·177–0·261)	2011
Denmark	0·016% (0·004–0·040)	0·016% (0·004–0·040)	2011
Estonia	0·267% (0·128–0·490)	0·959% (0·673–1·326)	2011
Finland	0·000% (0·000–0·019)	0·025% (0·008–0·059)	2011
France	0·070% (0·062–0·079)	0·034% (0·028–0·040)	2011
Germany	0·116% (0·107–0·126)	0·062% (0·055–0·069)	2011
Greece	1·374% (1·280–1·473)	1·202% (1·114–1·295)	2011
Hungary	0·009% (0·003–0·021)	0·159% (0·128–0·195)	2011
Iceland	0·072% (0·002–0·398)	0·000% (0·000–0·264)	2011
Ireland	0·039% (0·013–0·090)	0·008% (0·000–0·043)	2011
Italy	0·168% (0·155–0·181)	0·094% (0·085–0·104)	2011
Latvia^†^	1·127%	2·170%	2003
Liechtenstein	–	–	n/a
Lithuania	0·560% (0·468–0·665)	1·537% (1·382–1·704)	2011
Luxembourg	0·000% (0·000–0·406)	0·221% (0·027–0·794)	2011
Malta	0·174% (0·047–0·445)	0·043% (0·001–0·242)	2011
The Netherlands	0·034% (0·018–0·060)	0·020% (0·008–0·041)	2011
Norway	0·028% (0·009–0·065)	0·033% (0·012–0·073)	2011
Poland	0·450% (0·425–0·476)	0·742% (0·710–0·775)	2010
Portugal	0·094%	0·165%	2006
Romania	3·078% (2·965–3·195)	0·590% (0·541–0·643)	2011
Slovakia	0·072% (0·048–0·104)	0·025% (0·012–0·046)	2011
Slovenia	0·087% (0·043–0·155)	0·016% (0·002–0·057)	2009
Spain	0·168% (0·152–0·185)	0·099% (0·086–0·112)	2011
Sweden	0·043% (0·026–0·065)	0·059% (0·040–0·085)	2009
United Kingdom	0·038% (0·030–0·047)	0·037% (0·030–0·047)	2011

* Adapted from Table 1 and 7·1, Council of Europe Report 2014 [20].

† Latvia: no data are available after 2003.

The prevalence of anti-HCV among first-time blood donors ranged from 0·0% in Iceland to 2·2% in Latvia, and 58% of countries had an HCV prevalence that was about or below 0·1%.

### European HBV/HCV prevalence estimates

Using prevalence estimates for the general population and blood donors, the HBsAg prevalence in the EU/EEA as a whole is estimated to be 0·9% (95% CI 0·7–1·2), equivalent to almost 4·7 million chronic HBV cases. An overview of the estimated prevalence and data used for each country is in Supplementary Table S13. The United Kingdom has the largest estimated burden of chronic HBV in the EU/EEA with over a million cases, followed by Romania (877 682), and Spain, France and Italy (each with between 400 000 and 500 000 cases).

The anti-HCV prevalence in the EU/EEA is estimated at 1·1% (95% CI 0·9–1·4) equivalent to approximately 5·6 million anti-HCV-positive cases. Of these, an estimated 70% are chronically infected, i.e. viraemic replication with detectable HCV RNA [17]. France, Italy, Poland, Romania, Spain and the United Kingdom have the largest burden of chronic HCV with between 350 000 and 2·5 million anti-HCV-positive people.

## DISCUSSION

In this review, we have compiled recent evidence available on the prevalence of chronic HBV and HCV infection in the general population, pregnant women and blood donors from EU/EEA countries to provide further information on the epidemiology of these infections and to identify gaps in the available evidence.

The prevalence of chronic HBV and HCV in the general population varies widely across the 16 EU/EEA countries for which estimates were available, with a higher HBsAg and anti-HCV prevalence in countries in the Eastern and Southern part of the EU/EEA. There is great diversity across the region and the estimated prevalence in the country with the highest HBsAg estimate in the EU/EEA (Romania) is 44 times higher than in the country with the lowest estimated prevalence (Ireland). We also found that the highest single estimated anti-HCV prevalence (in Italy) is nearly 60 times higher than estimates from countries with the lowest prevalence (Belgium, Ireland and the Netherlands). Differing risk factors and transmission routes might partially explain this variation across countries, as well as different implementation of prevention and control strategies.

This study updates the previous ECDC systematic review [18] and adds new estimates of HBsAg in the general population for six countries (Germany, Greece, Ireland, Italy, the Netherlands and Romania). Although we did not conduct a statistical analysis of differences between the reviews, there is evidence of change over time in all countries except Ireland. A decline in HBV prevalence was observed in Germany (0·6–0·4%) and Romania (5·6–4·4%). Improved primary prevention programmes including antenatal screening and HBV vaccination are likely explanations for this decline, especially in Romania. A decline in estimated prevalence is also likely for Italy, but with only regional prevalence estimates reported in 2009 (due to heterogeneity across North, Central and South Italy), we cannot compare the pooled 0·8% HBsAg prevalence estimate derived from the samples included in this study. This study also observed heterogeneity in the estimates for Italy derived from higher quality studies (0·5–5·8%). An increase in estimated prevalence was observed in Greece (2·1–3·3%) and the Netherlands (0·1–0·2%). Limited geographical coverage is a likely explanation for the increase in Greece as neither estimate is from a national sample (the 2009 study covered the Peloponnesus [39]; the study in this review was conducted in Crete [28]). The slight increase in the Netherlands can be explained by the increase of the migrant population in the country, as was reported in the original study [40].

New high-quality anti-HCV prevalence estimates were available for five countries (France, Germany, Greece, the Netherlands and Spain). Again, although no statistical testing was conducted, results suggest that while the chronic HCV prevalence remained at 0·4% in Germany, it declined over time in France, the Netherlands and Spain, and increased in Greece. As with HBsAg prevalence, it is possible that the increase in Greece is mostly a reflection of the restricted geographical coverage of both the current estimate (Crete [28]) and the two reported in 2009 (Peloponnesus [39] and Zakynthos [41]). The new estimates for France and the Netherlands are derived from large-scale national random samples, whereas in 2009, the estimates were derived from regional/city-specific estimates where higher risk populations are over-represented. New estimates not previously available in 2009 were available for Hungary, Ireland, Croatia (not in the EU/EEA in 2009) and Latvia.

Both Ott *et al*. [42] and Schweitzer *et al*. [7] found that HBsAg prevalence increases eastwards across the EU/EEA; Schweitzer *et al.* reports the highest estimated prevalence found in Romania. While most HBsAg prevalence estimates are comparable, there are some notably different estimates reported by Schweitzer *et al*. for the 1990–2015 period, particularly for Greece (0·97% *vs.* 3·3% in this review) and the United Kingdom (0·01% *vs.* 1·74% in this review). Methodological differences, specifically the inclusion of a wider timeframe and a broader definition of the general population to include blood donors, pregnant women and health care workers, could explain these differences.

For anti-HCV, an increase in Eastern and Southern EU countries was also reported. Similar to the findings from this review, Gower *et al*. found the highest prevalence estimates for Romania (3·2%), Lithuania (2·9%) and Latvia (2·4%) [43]. However, there is some divergence in the reported estimates for Italy. Our pooled 5·9% prevalence is considerably higher than the published 2·2% [43], yet the wide ‘uncertainty range’ (notably not a CI) reported by Gower *et al*. does include 5·6%, suggesting some comparability. In our study, 14 highly heterogeneous estimates (0·6% [44] to 27·6% [45]) met the inclusion criteria, four of which were pooled. Gower *et al*., however, selected one ‘best estimate’ to represent a country. Ultimately, our findings suggest that the prevalence in Gower *et al*. could be an underestimate; on the other hand, our estimate for Italy might be skewed upwards by included studies conducted in remote areas of the centre of the country.

HCV estimates presented in Cornberg *et al*. for France, Germany, Hungary and Romania were very similar to the estimates in this paper or the same study was identified as the most reliable estimate [46, 47]. Cornberg *et al*. suggest that the best prevalence estimate for Italy is 4·4% (all ages) and 5·2% for adults, similar to the 5·9% we found. Cornberg *et al*. most diverges with our findings for Spain (2·6% *vs.* 1·1% in this review) [31]. Although they also present other estimates they conclude, along with Esteban *et al*., that the prevalence in Spain is around 2·5% [48], suggesting our findings may be an underestimate of the true prevalence in the country.

The differences in prevalence between countries and over time are difficult to interpret, because comparability between studies is limited by the use of different study designs, probabilistic and non-probabilistic sampling strategies, and use of different laboratory tests and sample types. In addition, geographical representativeness is limited as most studies were performed at sub-national level. Representative seroprevalence studies for the general population are thus needed for valid comparison.

### Pregnant women

Pregnant women are commonly considered as proxy for the general population, albeit to a different extent for HBV and HCV. The majority of EU/EEA MS offer antenatal HBV screening. Estimates of HBsAg prevalence among pregnant women, although slightly higher, mostly align with observed general population estimates in most countries, except in Greece and Spain, where the prevalence among pregnant women is lower. This is consistent with results of the 2009 systematic review [18]. The prevalence data for pregnant women in Spain are more recent than the general population estimates, so the difference between these groups might reflect a change in prevalence over time. For Greece, the prevalence data for pregnant women are from a national sample, while the general population data are derived from a regional sample. While a lower HBV prevalence could be expected in pregnant women, based on gender and age differences, groups with a higher risk of chronic viral hepatitis, such as migrants, are often under-represented in general population studies and may possibly be over-represented among samples in pregnant women.

Anti-HCV prevalence in pregnant women, where available for comparison with higher quality general population estimates, was found to be considerably lower. Chronic HCV infections in many EU/EEA countries have an age- and gender-specific prevalence distribution, with some studies from Southern Europe suggesting that 60% of the infected population is over 65 years of age [46]. Older and male populations, mostly infected through injecting drug use, contaminated blood or blood products, or improper infection control practices in health care, are not represented in studies in pregnant women [46], and our findings suggest that pregnant women are not a reliable proxy population to estimate prevalence in the general population.

### First-time blood donors

HBV and HCV seroprevalence data in first-time blood donors are readily available for most EU/EEA countries and are the most complete population prevalence source. Although blood donors are often used as a proxy population, this subgroup is generally considered not to be a representative sample due to self-selection of blood donors and strict regulation by blood banks [49]. These selection biases are reflected in our findings, which show that prevalence in first-time blood donors is considerably lower than general population estimates for all countries, although some confidence intervals overlap. Latvia may be the notable exception with a reported anti-HCV seroprevalence of 2·2% among first-time blood donors in 2003 (the latest estimate available), largely comparable with the higher quality estimate of 2·4% in 2008 in the general population [29].

### The burden of chronic hepatitis B and C in the EU/EEA

The HBV and HCV prevalence in the EU/EEA as a whole is estimated to be around 0·9% and 1·1%, respectively, resulting in an estimated total of 4·7 million chronic HBV cases and 5·6 million anti-HCV-positive cases. Considering that an estimated 70% of anti-HCV-positive cases are chronically infected [17], this corresponds with approximately 3·9 million chronic HCV cases.

The robustness of these figures is influenced not only by the intrinsic limitation of using prevalence estimates derived from an array of diverse studies, but also by the inclusion of prevalence estimates among blood donors as a proxy for the general population in the absence of other evidence. However, when taking into account both HBV and HCV data, general population estimates obtained from included studies accounted for approximately 83% of the total European population, with the remaining 17% covered by blood donor estimates.

Other than perhaps the population size of the country, no clear distribution across the EU can be observed in the availability of (higher quality) estimates in any of the targeted population groups. For one country, Liechtenstein, no information about HBV and HCV prevalence was available for any of the population groups. For Cyprus, Iceland and Malta, only prevalence data on first-time blood donors were available, and for Austria, Estonia, Lithuania, Poland and Sweden, only low-quality estimates were available.

### Strengths and limitations

An important strength of this review is that publications in all EU/EEA languages were included. In addition, consultation with MS further supplemented and validated the evidence retrieved. We feel that the lower sensitivity in the literature search due to the use of a geographical filter was effectively offset by the MS consultations. Another strength is that small-sample studies were excluded, a risk of bias assessment was developed and applied, and only high-quality estimates were selected and pooled (if multiple were available) for the analyses, to ease inter- and intra-country comparisons. The risk of bias assessment tool, however, has not been previously tested. An untested assumption in the tool is the equal weight given to each domain to calculate a final quality rating. For first-time blood donors, another data source was used rather than studies identified via a systematic review and the source was not assessed for bias.

This systematic literature review confirms the diversity in prevalence of chronic HBV and HCV infections across the EU/EEA, as well as the variability between groups often considered to provide a good proxy for the general population. Our findings suggest that using blood donor or pregnant women data as a proxy for HCV and, to a certain extent, HBV prevalence estimates for the general population is not desirable. Comparing to other regions globally, HBV and HCV prevalence in the EU/EEA is low, with some sign of decline, at least for HBV. The availability of studies with relatively recent data on the prevalence in the general population is limited, with data for around half of the 31 countries in the EU/EEA, reporting higher HBV and HCV prevalence in countries in the Eastern and Southern part of the region. The epidemiology of HBV and HCV is constantly changing, in part due to the impact of prevention and control programmes and changes in risk factors, but many countries lack recent robust epidemiological studies that provide reliable estimates of the burden of chronic viral hepatitis. The lack of high-quality, recent, nationwide prevalence estimates and the heterogeneity of available studies makes it challenging to gain an EU/EEA overview of the current epidemiological situation regarding chronic viral hepatitis. The need for high-quality strategic information on the burden of HBV and HCV is compelling, not only for scaling up secondary prevention services appropriately, but also to inform regional and global activities that will shape the response to these epidemics in years to come. This could be achieved by complementing case-based surveillance with alternative data sources with adequate standardization levels across the region. A standardized seroprevalence survey performed across the EU/EEA, while resource intensive, may be a well-needed intervention to consider.

## CONTRIBUTORS

LT and EFD coordinated the study. LT, EFD and AJA-G developed the overall concept and framework of the study. LT, EFD, SHIH, AMF, IKV and SJMH designed the study protocol. LT developed the search strategy and performed the literature searches. SHIH and AMF acquired and analysed the data. SHIH, AMF, LT and EFD interpreted the data. SJMH and IKV provided technical expertise and advice on relevance and accuracy of studies. SHIH drafted the first version of the manuscript. AMF, EFD, SJMH, AJA-G, IKV and LT contributed to critical revision of the manuscript for important intellectual content. All authors read and approved the final manuscript.

## Supplementary material

For supplementary material accompanying this paper visit https://doi.org/10.1017/S0950268817001947. click here to view supplementary material

## References

[1] EASL Clinical Practice Guidelines. Management of chronic hepatitis B virus infection. Journal of Hepatology 2012; 57: 167–185. 22436845 10.1016/j.jhep.2012.02.010

[2] EASL Clinical Practice Guidelines. Management of hepatitis C virus infection. Journal of Hepatology 2014; 60: 392–420. 24331294 10.1016/j.jhep.2013.11.003

[3] World Health Organization (http://www.who.int/mediacentre/factsheets/fs204_Jul2014/en/). Accessed June 2015.

[4] World Health Organization (http://www.who.int/mediacentre/factsheets/fs164/en/). Accessed June 2015.

[5] European Centre for Disease Prevention and Control. Surveillance and Prevention of Hepatitis B and C in Europe. Stockholm: ECDC, 2010.

[6] Edmunds WJ, The influence of age on the development of the hepatitis B carrier state. Proceedings Biological Sciences 1993; 253: 197–201. 8397416 10.1098/rspb.1993.0102

[7] Schweitzer A, Estimations of worldwide prevalence of chronic hepatitis B virus infection: a systematic review of data published between 1965 and 2013. The Lancet 2015; 386: 1546–1555. 10.1016/S0140-6736(15)61412-X 26231459

[8] Lozano R, Global and regional mortality from 235 causes of death for 20 age groups in 1990 and 2010: a systematic analysis for the Global Burden of Disease Study 2010. The Lancet 2012; 380: 2095–2128. 10.1016/S0140-6736(12)61728-0 PMC10790329 23245604

[9] Mohd Hanafiah K, Global epidemiology of hepatitis C virus infection: new estimates of age-specific antibody to HCV seroprevalence. Hepatology 2013; 57: 1333–1342. 23172780 10.1002/hep.26141

[10] European Centre for Disease Prevention and Control. Hepatitis B Surveillance in Europe – 2014. Stockholm: ECDC, 2016.

[11] European Centre for Disease Prevention and Control. Hepatitis C Surveillance in Europe – 2014. Stockholm: ECDC, 2016.

[12] Wedemeyer H, Strategies to manage hepatitis C virus (HCV) disease burden. Journal of Viral Hepatitis 2014; 21(Suppl. 1): 60–89. 24713006 10.1111/jvh.12249

[13] Kohli A, Treatment of hepatitis C: a systematic review. JAMA 2014; 312: 631–640. 25117132 10.1001/jama.2014.7085

[14] Wolffram I, Prevalence of elevated ALT values, HBsAg, and anti-HCV in the primary care setting and evaluation of guideline defined hepatitis risk scenarios. Journal of Hepatology 2015; 62: 1256–1264. 25617500 10.1016/j.jhep.2015.01.011

[15] European Liver Patients Association (ELPA). Report on Hepatitis Patient Self-Help in Europe 2010 (http://www.hepbcppa.org/wp-content/uploads/2011/11/Report-on-Patient-Self-Help.pdf). Accessed September 2015.

[16] World Health Organization. Global Health Sector Strategy on Viral Hepatitis 2016–2021 – Towards Ending Viral Hepatitis. Geneva: WHO, 2016.

[17] World Health Organization – Regional Office for Europe. Action Plan for the Health Sector Response to Viral Hepatitis in the WHO European Region. Copenhagen: WHO – Regional Office for Europe, 2016.

[18] Hahné SJ, Infection with hepatitis B and C virus in Europe: a systematic review of prevalence and cost-effectiveness of screening. BMC Infectious Diseases 2013; 13: 181. 23597411 10.1186/1471-2334-13-181 PMC3716892

[19] European Centre for Disease Prevention and Control. (https://emma.ecdc.europa.eu/Pages/home.aspx). Accessed September 2015.

[20] Van Hoeven LR, Janssen MP, Rautmann G. The Collection, Testing and Use of Blood and Blood Components in Europe. Strasbourg: European Directorate for the Quality of Medicines & HealthCare (EDQM) – Council of Europe, 2011.

[21] Talento AF, P18.05 serological screening of solid organ transplant donors in Ireland. The Journal of Hospital Infection 2010; 76(Suppl. 1): S58–S59.

[22] Gheorghe L, The prevalence and risk factors of hepatitis B virus infection in an adult population in Romania: a nationwide survey. European Journal of Gastroenterology & Hepatology 2013; 25: 56–64. 22968488 10.1097/MEG.0b013e328358b0bb

[23] Cozzolongo R, Epidemiology of HCV infection in the general population: a survey in a southern Italian town. The American Journal of Gastroenterology 2009; 104: 2740–2746. 19638964 10.1038/ajg.2009.428

[24] Del Corno G, Civardi E. Intrafamilial transmission of hepatitis B and C viruses in an Italian local health district. Annali Di Igiene 2006; 18: 287–295. 17063627

[25] Quoilin S, A population-based prevalence study of hepatitis A, B and C virus using oral fluid in Flanders, Belgium. European Journal of Epidemiology 2007; 22: 195–202. 17356926 10.1007/s10654-007-9105-6

[26] Vriend HJ, Hepatitis C virus prevalence in The Netherlands: migrants account for most infections. Epidemiology and Infection 2013; 141: 1310–1317. 22963908 10.1017/S0950268812001884 PMC9151883

[27] Gheorghe L, The prevalence and risk factors of hepatitis C virus infection in adult population in Romania: a nationwide survey 2006–2008. Journal of Gastrointestinal and Liver Diseases 2010; 19: 373–379. 21188327

[28] Drositis I, Epidemiology and molecular analysis of hepatitis A, B and C in a semi-urban and rural area of Crete. European Journal of Internal Medicine 2013; 24: 839–845. 23988264 10.1016/j.ejim.2013.08.003

[29] Tolmane I, The prevalence of viral hepatitis C in Latvia: a population-based study. Medicina 2011; 47: 532–535. 22186116

[30] Schreter I, Prevalence of hepatitis C virus infection in Slovakia. Klinicka Mikrobiologie a Infekcni Lekarstvi 2007; 13: 54–58. 17599293

[31] Lopez-Izquierdo R, Seroprevalence of viral hepatitis in a representative general population of an urban public health area in Castilla y Leon (Spain). Enfermedades Infecciosas Y Microbiologia Clinica 2007; 25: 317–323. 17504685 10.1157/13102267

[32] Caballeria L, Strategies for the detection of hepatitis C viral infection in the general population. Revista Clinica Espanola 2014; 214: 242–246. 24598246 10.1016/j.rce.2014.01.024

[33] Calleja-Panero JL, Prevalence of viral hepatitis (B and C) serological markers in healthy working population. Revista Espanola De Enfermedades Digestivas 2013; 105: 249–254. 23971655 10.4321/s1130-01082013000500002

[34] Kristiansen MG, Prevalences of viremic hepatitis C and viremic hepatitis B in pregnant women in Northern Norway. Hepato-Gastroenterology 2009; 56: 1141–1145. 19760958

[35] Salleras L, Seroepidemiology of hepatitis B virus infection in pregnant women in Catalonia (Spain. Journal of Clinical Virology 2009; 44: 329–332. 19230752 10.1016/j.jcv.2009.01.002

[36] Richaud-Eyraud E, Infectious diseases screening during pregnancy: results from the ELFE survey in maternity units, mainland France, 2011. Bulletin Epidemiologique Hebdomadaire 2015; 15: 254–263.

[37] Op de Coul EL, Antenatal screening for HIV, hepatitis B and syphilis in the Netherlands is effective. BMC Infectious Diseases 2011; 11: 185. 21718466 10.1186/1471-2334-11-185 PMC3160399

[38] Kopilovic B, Hepatitis C virus infection among pregnant women in Slovenia: study on 31,849 samples obtained in four screening rounds during 1999, 2003, 2009 and 2013. Euro Surveillance 2015; 20: 21144. 26062646 10.2807/1560-7917.es2015.20.22.21144

[39] Gogos CA, Prevalence of hepatitis B and C virus infection in the general population and selected groups in South-Western Greece. European Journal of Epidemiology 2003; 18: 551–557. 12908721 10.1023/a:1024698715741

[40] Hahné SJ, Prevalence of hepatitis B virus infection in The Netherlands in 1996 and 2007. Epidemiology and Infection 2012; 140: 1469–1480. 22078095 10.1017/S095026881100224X

[41] Goritsas C, HCV infection in the general population of a Greek island: prevalence and risk factors. Hepato-Gastroenterology 2000; 47: 782–785. 10919032

[42] Ott JJ, Time trends of chronic HBV infection over prior decades – a global analysis. Journal of Hepatology 2017; 66: 48–54. 27592304 10.1016/j.jhep.2016.08.013

[43] Gower E, Global epidemiology and genotype distribution of the hepatitis C virus infection. Journal of Hepatology 2014; 61(Suppl. 1): S45–S57. 25086286 10.1016/j.jhep.2014.07.027

[44] Parisi MR, Point-of-care testing for HCV infection: recent advances and implications for alternative screening. The New Microbiologica 2014; 37: 449–457. 25387283

[45] Petti S, Analysis of the shift of the transmission pattern for hepatitis C in a community in Central Italy. The New Microbiologica 2006; 29: 207–209. 17058788

[46] Cornberg M, A systematic review of hepatitis C virus epidemiology in Europe, Canada and Israel. Liver International 2011; 31(Suppl. 2): 30–60. 21651702 10.1111/j.1478-3231.2011.02539.x

[47] Meffre C, Prevalence of hepatitis B and hepatitis C virus infections in France in 2004: social factors are important predictors after adjusting for known risk factors. Journal of Medical Virology 2010; 82: 546–555. 20166185 10.1002/jmv.21734

[48] Esteban JI, Sauleda S, Quer J. The changing epidemiology of hepatitis C virus infection in Europe. Journal of Hepatology 2008; 48: 148–162. 18022726 10.1016/j.jhep.2007.07.033

[49] De Kort W, Blood donor selection in European Union directives: room for improvement. Blood Transfusion 2016; 14: 101–108. 26509824 10.2450/2015.0148-15 PMC4781776

