# Supplementary material for: Current prevalence of chronic hepatitis B and C virus infection in the general population, blood donors and pregnant women in the EU/EEA: a systematic review
Source: Epidemiol Infect. 2017 Sep 11;145(14):2873–85. doi: 10.1017/S0950268817001947 (PMC5647665; doi:10.1017/S0950268817001947)
Supplement: Supplementary file 1 [file S0950268817001947sup001.docx]

*‘Epidemiology and Infection’*

**Current prevalence of chronic hepatitis B and C virus infection in the general population, blood donors and pregnant women in the EU/EEA: a systematic review.**

S.H.I. Hofstraat, A.M. Falla, E. F. Duffell, S. J.M. Hahné, A. J. Amato-Gauci, I. K. Veldhuijzen, L. Tavoschi

*Supplementary Material*

**Supplementary Figure S1** Literature search strategies p2

**Supplementary Table S2** Study inclusion and exclusion criteria p29

**Supplementary Table S3** List and definition of key subgroups included in the systematic review p29

**Supplementary Table S4** Variables used in Data Extraction p30

**Supplementary Table S5** Risk of bias framework for general population studies p31

**Supplementary Table S6** Risk of bias framework for studies among pregnant women p31

**Supplementary Table S7** Extracted and pooled (where possible) HBsAg prevalence estimates for p32

the general population from included studies

**Supplementary Table S8** Extracted and pooled (where possible) anti-HCV prevalence estimates for p38

the general population from included studies

**Supplementary Table S9** Risk of bias assessment and characteristics of the general population p44

studies included in the HBV and HCV prevalence analysis

**Supplementary Table S10** Extracted and pooled (where possible) HBsAg prevalence estimates for p46

pregnant women from included studies

**Supplementary Table S11** Extracted and pooled (where possible) anti-HCV prevalence estimates for p50

pregnant women from included studies

**Supplementary Table S12**. Risk of bias assessment and characteristics of the studies in pregnant p52

women included in the HBV and HCV prevalence analysis

**Supplementary Table S13** Data used for HBsAg and anti-HCV prevalence estimate for the EU/EAA p53

**Supplementary Figure S1.** Literature search strategies

The geographical search strategy was developed by ECDC and underwent one testing round with the following results: NPV of 98∙4% and a PPV of 66∙4% in Embase^©^ and NPV of 97∙6% and a PPV of 65∙2% in PubMed®. The geographical search strategy was not applied to the Cochrane Library.

## Pubmed search

Date of the search: 16/03/2015

Language limit: no limitis

Dates limits: from 2005 to 2015

No of results: 4541

| Search | Query | Items found |
| --- | --- | --- |
| [#4](http://www.ncbi.nlm.nih.gov/pubmed/advanced) | Search **#1 AND #2 AND #3** | [10461](http://www.ncbi.nlm.nih.gov/pubmed/?cmd=HistorySearch&querykey=4) |
| [#5](http://www.ncbi.nlm.nih.gov/pubmed/advanced) | Search **#1 AND #2 AND #3** Filters: **Publication date from 2005/01/01** | [4541](http://www.ncbi.nlm.nih.gov/pubmed/?cmd=HistorySearch&querykey=5) |
| [#3](http://www.ncbi.nlm.nih.gov/pubmed/advanced) | Search **"hepatitis B"[MeSH Terms] OR "hepatitis c"[MeSH Terms] OR "Hepatitis B virus"[Mesh] OR "hepacivirus"[Mesh] OR "hepatitis b"[TIAB] OR "hepatitis c"[TIAB] OR hepaciviru*[TIAB] OR "hbv"[TIAB] OR "hcv"[TIAB] OR "hbsag"[TIAB] OR "hbs ag"[TIAB] OR "hepatitis b surface antigens"[MeSH Terms] OR “Australia Antigen”[TIAB] OR “Australia Antigens”[TIAB] OR "hepatitis c antibodies"[MeSH Terms] OR "Hepatitis C Antigens"[Mesh] OR "Hepatitis B Antibodies"[Mesh]** | [135899](http://www.ncbi.nlm.nih.gov/pubmed/?cmd=HistorySearch&querykey=3) |
| [#2](http://www.ncbi.nlm.nih.gov/pubmed/advanced) | Search **"Prevalence"[Mesh] OR Prevalence*[TIAB] OR "Population Surveillance"[Mesh] OR "Seroepidemiologic Studies"[Mesh:NoExp] OR seroprevalence*[TIAB] OR seroepidemiolog*[TIAB] OR "sero epidemiologic"[TIAB] OR "sero epidemiological"[TIAB] OR "sero epidemiology"[TIAB] OR serosurvey*[TIAB] OR serolog*[TI] OR epidemiolog*[TI] OR surveillance[TI]** | [643490](http://www.ncbi.nlm.nih.gov/pubmed/?cmd=HistorySearch&querykey=2) |
| [#1](http://www.ncbi.nlm.nih.gov/pubmed/advanced) | Search **(((("United kingdom"[ad] OR Britain[ad] OR British[ad] OR (England[ad] NOT ("New England"[ad])) OR English[ad] OR Scotland[ad] OR Scottish[ad] OR Wales[ad] OR Welsh[ad] OR "Northen Ireland"[ad] OR London[ad] OR "East midlands"[ad] OR "West midlands"[ad] OR Yorkshire[ad] OR "East Anglia"[ad] OR Bedfordshire[ad] OR Hertfordshire[ad] OR Essex[ad] OR Peterborough[ad] OR Cambridgeshire[ad] OR Norfolk[ad] OR Suffolk[ad] OR Luton[ad] OR Bedford[ad] OR "Southend on sea"[ad] OR Thurrock[ad] OR Derbyshire[ad] OR Nottinghamshire[ad] OR Leicestershire[ad] OR Rutland[ad] OR Northamptonshire[ad] OR Lincolnshire[ad] OR Derby[ad] OR Leicester[ad] OR Northamptonshire[ad] OR Nottingham[ad] OR Northumberland[ad] OR "Tyne and Wear"[ad] OR "Tees Valley"[ad] OR "Durham"[ad] OR Darlington[ad] OR Hartlepool[ad] OR "Stockton on tees"[ad] OR Northumberland[ad] OR Teesside[ad] OR Sunderland[ad] OR Tyneside[ad] OR Cumbria[ad] OR Cheshire[ad] OR Manchester[ad] OR Lancashire[ad] OR Merseyside[ad] OR (Blackburn[ad] AND Darwen[ad]) OR Blackpool[ad] OR Chester[ad] OR Liverpool[ad] OR Sefton[ad] OR Warrington[ad] OR Wirral[ad] OR Berkshire[ad] OR Buckinghamshire[ad] OR Oxfordshire[ad] OR Hampshire[ad] OR "Isle of Wight"[ad] OR Kent[ad] OR Surrey[ad] OR Sussex[ad] OR (Brighton[ad] AND Hove[ad]) OR Medway[ad] OR "Milton keynes"[ad] OR Portsmouth[ad] OR Southampton[ad] OR Devon[ad] OR Dorset[ad] OR Somerset[ad] OR Gloucestershire[ad] OR Wiltshire[ad] OR Bristol[ad] OR Bath[ad] OR Bournemouth[ad] OR Poole[ad] OR Bristol[ad] OR Plymouth[ad] OR Swindon[ad] OR Torbay[ad] OR Herefordshire[ad] OR Worcestershire[ad] OR Warwickshire[ad] OR Shropshire[ad] OR Staffordshire[ad] OR Birmingham[ad] OR Coventry[ad] OR Dudley[ad] OR Sandwell[ad] OR Shropshire[ad] OR Solihull[ad] OR "stoke on trent"[ad] OR Telford[ad] OR Wrekin[ad] OR Walsall[ad] OR Warwickshire[ad] OR Wolverhampton[ad] OR Worcestershire[ad] OR Barnsley[ad] OR Doncaster[ad] OR Rotherham[ad] OR Bradford[ad] OR Calderdale[ad] OR Kirklees[ad] OR Kingston[ad] OR Leeds[ad] OR Sheffield[ad] OR Wakefield[ad] OR (York[ad] NOT ("New York"[ad])) OR Antrim[ad] OR Ards[ad] OR Armagh[ad] OR Ballymena[ad] OR Ballymoney[ad] OR Banbridge[ad] OR Carrickfergus[ad] OR Castlereagh[ad] OR Coleraine[ad] OR Cookstown[ad] OR Craigavon[ad] OR Derry[ad] OR (Down[ad] AND (district[ad] OR council[ad])) OR Fermanagh[ad] OR Dungannon[ad] OR Fermanagh[ad] OR Larne[ad] OR Limavady[ad] OR Lisburn[ad] OR Magherafelt[ad] OR Moyle[ad] OR (Newry[ad] AND Mourne[ad]) OR Newtownabbey[ad] OR Omagh[ad] OR Strabane[ad] OR Londonderry[ad] OR Tyrone[ad] OR Belfast[ad] OR Aberdeen[ad] OR Aberdeenshire[ad] OR Angus[ad] OR Dundee[ad] OR (Argyll[ad] AND bute[ad]) OR Clackmannanshire[ad] OR Fife[ad] OR Ayrshire[ad] OR Dunbartonshire[ad] OR Lothian[ad] OR Renfrewshire[ad] OR Edinburgh[ad] OR Falkirk[ad] OR Glasgow[ad] OR Highland*[ad] OR Inverclyde[ad] OR Midlothian[ad] OR Moray[ad] OR Lanarkshire[ad] OR (Perth[ad] AND Kinross[ad]) OR Stirling[ad] OR "Orkney Islands"[ad] OR "Eileanan Siar"[ad] OR "Shetland Islands"[ad] OR Bridgend[ad] OR "Neath Port Talbot"[ad] OR Cardiff[ad] OR (Vale[ad] AND Glamorgan[ad]) OR "Central Valleys"[ad] OR Conwy[ad] OR Denbighshire[ad] OR Flintshire[ad] OR Wrexham[ad] OR "Gwent Valleys"[ad] OR Gwynedd[ad] OR (Isle[ad] AND Anglesey[ad]) OR "Monmouthshire"[ad] OR "Newport"[ad] OR Powys[ad] OR Swansea[ad] OR Ceredigion[ad] OR Carmarthenshire[ad] OR Pembrokeshire[ad] OR "Merthyr Tydfil"[ad] OR "Rhondda Cynon Taff"[ad] OR "Blaenau Gwent"[ad] OR Caerphilly[ad] OR Torfaen[ad] OR Caithness[ad] OR "Sutherland and Ross"[ad] OR Cromarty[ad] OR Teeside[ad] OR Tyneside[ad] OR Wearside[ad] OR "West Mercia"[ad] OR Avon[ad] OR Ulster[ad] OR Derry[ad] OR Medway[ad] OR "East Riding"[ad] OR "West Riding"[ad] OR "Lake District"[ad] OR "Peak District"[ad] OR Cumberland[ad] OR Dartmoor[ad] OR Exmoor[ad])) OR ("United kingdom"[tw] OR Britain[tw] OR British[tw] OR (England[tw] NOT ("New England"[tw])) OR English[tw] OR Scotland[tw] OR Scottish[tw] OR Wales[tw] OR Welsh[tw] OR "Northen Ireland"[tw] OR London[tw] OR "East midlands"[tw] OR "West midlands"[tw] OR Yorkshire[tw] OR "East Anglia"[tw] OR Bedfordshire[tw] OR Hertfordshire[tw] OR Essex[tw] OR Peterborough[tw] OR Cambridgeshire[tw] OR Norfolk[tw] OR Suffolk[tw] OR Luton[tw] OR Bedford[tw] OR "Southend on sea"[tw] OR Thurrock[tw] OR Derbyshire[tw] OR Nottinghamshire[tw] OR Leicestershire[tw] OR Rutland[tw] OR Northamptonshire[tw] OR Lincolnshire[tw] OR Derby[tw] OR Leicester[tw] OR Northamptonshire[tw] OR Nottingham[tw] OR Northumberland[tw] OR "Tyne and Wear"[tw] OR "Tees Valley"[tw] OR "Durham"[tw] OR Darlington[tw] OR Hartlepool[tw] OR "Stockton on tees"[tw] OR Northumberland[tw] OR Teesside[tw] OR Sunderland[tw] OR Tyneside[tw] OR Cumbria[tw] OR Cheshire[tw] OR Manchester[tw] OR Lancashire[tw] OR Merseyside[tw] OR (Blackburn[tw] AND Darwen[tw]) OR Blackpool[tw] OR Chester[tw] OR Liverpool[tw] OR Sefton[tw] OR Warrington[tw] OR Wirral[tw] OR Berkshire[tw] OR Buckinghamshire[tw] OR Oxfordshire[tw] OR Hampshire[tw] OR "Isle of Wight"[tw] OR Kent[tw] OR Surrey[tw] OR Sussex[tw] OR (Brighton[tw] AND Hove[tw]) OR Medway[tw] OR "Milton keynes"[tw] OR Portsmouth[tw] OR Southampton[tw] OR Devon[tw] OR Dorset[tw] OR Somerset[tw] OR Gloucestershire[tw] OR Wiltshire[tw] OR Bristol[tw] OR Bath[tw] OR Bournemouth[tw] OR Poole[tw] OR Bristol[tw] OR Plymouth[tw] OR Swindon[tw] OR Torbay[tw] OR Herefordshire[tw] OR Worcestershire[tw] OR Warwickshire[tw] OR Shropshire[tw] OR Staffordshire[tw] OR Birmingham[tw] OR Coventry[tw] OR Dudley[tw] OR Sandwell[tw] OR Shropshire[tw] OR Solihull[tw] OR "stoke on trent"[tw] OR Telford[tw] OR Wrekin[tw] OR Walsall[tw] OR Warwickshire[tw] OR Wolverhampton[tw] OR Worcestershire[tw] OR Barnsley[tw] OR Doncaster[tw] OR Rotherham[tw] OR Bradford[tw] OR Calderdale[tw] OR Kirklees[tw] OR Kingston[tw] OR Leeds[tw] OR Sheffield[tw] OR Wakefield[tw] OR (York[tw] NOT ("New York"[tw])) OR Antrim[tw] OR Ards[tw] OR Armagh[tw] OR Ballymena[tw] OR Ballymoney[tw] OR Banbridge[tw] OR Carrickfergus[tw] OR Castlereagh[tw] OR Coleraine[tw] OR Cookstown[tw] OR Craigavon[tw] OR Derry[tw] OR (Down[tw] AND (district[tw] OR council[tw])) OR Fermanagh[tw] OR Dungannon[tw] OR Fermanagh[tw] OR Larne[tw] OR Limavady[tw] OR Lisburn[tw] OR Magherafelt[tw] OR Moyle[tw] OR (Newry[tw] AND Mourne[tw]) OR Newtownabbey[tw] OR Omagh[tw] OR Strabane[tw] OR Londonderry[tw] OR Tyrone[tw] OR Belfast[tw] OR Aberdeen[tw] OR Aberdeenshire[tw] OR Angus[tw] OR Dundee[tw] OR (Argyll[tw] AND bute[tw]) OR Clackmannanshire[tw] OR Fife[tw] OR Ayrshire[tw] OR Dunbartonshire[tw] OR Lothian[tw] OR Renfrewshire[tw] OR Edinburgh[tw] OR Falkirk[tw] OR Glasgow[tw] OR Highland*[tw] OR Inverclyde[tw] OR Midlothian[tw] OR Moray[tw] OR Lanarkshire[tw] OR (Perth[tw] AND Kinross[tw]) OR Stirling[tw] OR "Orkney Islands"[tw] OR "Eileanan Siar"[tw] OR "Shetland Islands"[tw] OR Bridgend[tw] OR "Neath Port Talbot"[tw] OR Cardiff[tw] OR (Vale[tw] AND Glamorgan[tw]) OR "Central Valleys"[tw] OR Conwy[tw] OR Denbighshire[tw] OR Flintshire[tw] OR Wrexham[tw] OR "Gwent Valleys"[tw] OR Gwynedd[tw] OR (Isle[tw] AND Anglesey[tw]) OR "Monmouthshire"[tw] OR "Newport"[tw] OR Powys[tw] OR Swansea[tw] OR Ceredigion[tw] OR Carmarthenshire[tw] OR Pembrokeshire[tw] OR "Merthyr Tydfil"[tw] OR "Rhondda Cynon Taff"[tw] OR "Blaenau Gwent"[tw] OR Caerphilly[tw] OR Torfaen[tw] OR Caithness[tw] OR "Sutherland and Ross"[tw] OR Cromarty[tw] OR Teeside[tw] OR Tyneside[tw] OR Wearside[tw] OR "West Mercia"[tw] OR Avon[tw] OR Ulster[tw] OR Derry[tw] OR Medway[tw] OR "East Riding"[tw] OR "West Riding"[tw] OR "Lake District"[tw] OR "Peak District"[tw] OR Cumberland[tw] OR Dartmoor[tw] OR Exmoor[tw])) OR (((Sweden[ad] OR Sverige[ad] OR Swedish[ad] OR Svenska[ad] OR Stockholm*[ad] OR Norrland[ad] OR Svealand[ad] OR Mellansverige[ad] OR Smaland[ad] OR Sydsverige[ad] OR Vastsverige[ad] OR Orebro[ad] OR Ostergotland*[ad] OR Vastergotland*[ad] OR Skara*[ad] OR Bohus*[ad] OR Dalsland[ad] OR Narke[ad] OR Sodermanland[ad] OR Uppsala[ad] OR Uppland[ad] OR Vastmanland*[ad] OR Jamtland*[ad] OR Harjedalen[ad] OR Vasternorrland*[ad] OR Dalarna[ad] OR Kopparberg[ad] OR Gavleborg*[ad] OR Gastrikland[ad] OR Halsingland[ad] OR Varmland*[ad] OR Gotland*[ad] OR Oland[ad] OR Jonkoping*[ad] OR Kalmar*[ad] OR Kronoberg*[ad] OR Blekinge[ad] OR Skane*[ad] OR Norrbotten*[ad] OR Vasterbotten*[ad] OR Lappland[ad] OR Angermanland[ad] OR Medelpad[ad] OR Halland*[ad] OR Gotaland*[ad] OR Gothenburg[ad] OR Goteborg*[ad] OR Malmo*[ad] OR Vasteras[ad] OR Linkoping[ad] OR Helsingborg[ad] OR Halsingborg[ad] OR Norrkoping[ad] OR Gavle[ad] OR Umea[ad] OR Lulea[ad] OR Karlstad[ad] OR Kalmar[ad] OR Huddinge[ad] OR Solna[ad] OR Ostersjo*[ad] OR Malaren*[ad] OR Malardalen[ad])) OR (Sweden[tw] OR Sverige[tw] OR Swedish[tw] OR Svenska[tw] OR Stockholm*[tw] OR Norrland[tw] OR Svealand[tw] OR Mellansverige[tw] OR Smaland[tw] OR Sydsverige[tw] OR Vastsverige[tw] OR Orebro[tw] OR Ostergotland*[tw] OR Vastergotland*[tw] OR Skara*[tw] OR Bohus*[tw] OR Dalsland[tw] OR Narke[tw] OR Sodermanland[tw] OR Uppsala[tw] OR Uppland[tw] OR Vastmanland*[tw] OR Jamtland*[tw] OR Harjedalen[tw] OR Vasternorrland*[tw] OR Dalarna[tw] OR Kopparberg[tw] OR Gavleborg*[tw] OR Gastrikland[tw] OR Halsingland[tw] OR Varmland*[tw] OR Gotland*[tw] OR Oland[tw] OR Jonkoping*[tw] OR Kalmar*[tw] OR Kronoberg*[tw] OR Blekinge[tw] OR Skane*[tw] OR Norrbotten*[tw] OR Vasterbotten*[tw] OR Lappland[tw] OR Angermanland[tw] OR Medelpad[tw] OR Halland*[tw] OR Gotaland*[tw] OR Gothenburg[tw] OR Goteborg*[tw] OR Malmo*[tw] OR Vasteras[tw] OR Linkoping[tw] OR Helsingborg[tw] OR Halsingborg[tw] OR Norrkoping[tw] OR Gavle[tw] OR Umea[tw] OR Lulea[tw] OR Karlstad[tw] OR Kalmar[tw] OR Huddinge[tw] OR Solna[tw] OR Ostersjo*[tw] OR Malaren*[tw] OR Malardalen[tw])) OR (((Spain[ad] OR Espana[ad] OR Spanish[ad] OR Espanol*[ad] OR Spaniard*[ad] OR Madrid[ad] OR Andalucia[ad] OR Andalusia[ad] OR Aragon[ad] OR Cantabria[ad] OR Canarias[ad] OR "Canary Islands"[ad] OR "Castile and leon"[ad] OR "Castilla y Leon"[ad] OR "Castile La Mancha"[ad] OR "Castilla La Mancha"[ad] OR Cataluna[ad] OR Catalonia[ad] OR Ceuta[ad] OR Melilla[ad] OR Navarra[ad] OR Navarre[ad] OR Valencia[ad] OR Valencian[ad] OR Extremadura[ad] OR Galicia[ad] OR Balears[ad] OR "Balearic Islands"[ad] OR Baleares[ad] OR "La Rioja"[ad] OR "Pais Vasco"[ad] OR "Basque Country"[ad] OR Asturias[ad] OR Murcia[ad] OR Coruna[ad] OR Alava[ad] OR Araba[ad] OR Albacete[ad] OR Alicante[ad] OR Alacant[ad] OR Almeria[ad] OR Asturias[ad] OR Avila[ad] OR Badajoz[ad] OR Badajos[ad] OR Barcelona[ad] OR Burgos[ad] OR Caceres[ad] OR Cadiz[ad] OR Castellon[ad] OR Castello[ad] OR "Ciudad Real"[ad] OR Cordoba[ad] OR Cuenca[ad] OR Eivissa[ad] OR Ibiza[ad] OR Formentera[ad] OR "El Hierro"[ad] OR Fuerteventura[ad] OR Girona[ad] OR Gerona[ad] OR "Gran Canaria"[ad] OR Granada[ad] OR Guadalajara[ad] OR Guipuzcoa[ad] OR Gipuzkoa[ad] OR Huelva[ad] OR Huesca[ad] OR Jaen[ad] OR "La Gomera"[ad] OR "La Palma"[ad] OR Lanzarote[ad] OR Leon[ad] OR Lleida[ad] OR Lerida[ad] OR Lugo[ad] OR Malaga[ad] OR Mallorca[ad] OR Majorca[ad] OR Menorca[ad] OR Minorca[ad] OR Murcia[ad] OR Ourense[ad] OR Orense[ad] OR Palencia[ad] OR Pontevedra[ad] OR Salamanca[ad] OR Segovia[ad] OR Sevilla[ad] OR Seville[ad] OR Soria[ad] OR Tarragona[ad] OR Tenerife[ad] OR Teruel[ad] OR Toledo[ad] OR Valencia[ad] OR Valladolid[ad] OR Vizcaya[ad] OR Biscay[ad] OR Zamora[ad] OR Zaragoza[ad] OR Saragossa[ad] OR Bilbao[ad] OR Bilbo[ad] OR Compostela[ad] OR "San Sebastian"[ad] OR Donostia[ad] OR Vitoria[ad] OR Oviedo[ad] OR Pamplona[ad] OR Logrono[ad] OR Gasteiz[ad])) OR (Spain[tw] OR Espana[tw] OR Spanish[tw] OR Espanol*[tw] OR Spaniard*[tw] OR Madrid[tw] OR Andalucia[tw] OR Andalusia[tw] OR Aragon[tw] OR Cantabria[tw] OR Canarias[tw] OR "Canary Islands"[tw] OR "Castile and leon"[tw] OR "Castilla y Leon"[tw] OR "Castile La Mancha"[tw] OR "Castilla La Mancha"[tw] OR Cataluna[tw] OR Catalonia[tw] OR Ceuta[tw] OR Melilla[tw] OR Navarra[tw] OR Navarre[tw] OR Valencia[tw] OR Valencian[tw] OR Extremadura[tw] OR Galicia[tw] OR Balears[tw] OR "Balearic Islands"[tw] OR Baleares[tw] OR "La Rioja"[tw] OR "Pais Vasco"[tw] OR "Basque Country"[tw] OR Asturias[tw] OR Murcia[tw] OR Coruna[tw] OR Alava[tw] OR Araba[tw] OR Albacete[tw] OR Alicante[tw] OR Alacant[tw] OR Almeria[tw] OR Asturias[tw] OR Avila[tw] OR Badajoz[tw] OR Badajos[tw] OR Barcelona[tw] OR Burgos[tw] OR Caceres[tw] OR Cadiz[tw] OR Castellon[tw] OR Castello[tw] OR "Ciudad Real"[tw] OR (Cordoba[tw] NOT Argent*[tw]) OR Cuenca[tw] OR Eivissa[tw] OR Ibiza[tw] OR Formentera[tw] OR "El Hierro"[tw] OR Fuerteventura[tw] OR Girona[tw] OR Gerona[tw] OR "Gran Canaria"[tw] OR Granada[tw] OR (Guadalajara[tw] NOT Mexic*[tw]) OR Guipuzcoa[tw] OR Gipuzkoa[tw] OR Huelva[tw] OR Huesca[tw] OR Jaen[tw] OR "La Gomera"[tw] OR "La Palma"[tw] OR Lanzarote[tw] OR Leon[tw] OR Lleida[tw] OR Lerida[tw] OR Lugo[tw] OR Malaga[tw] OR Mallorca[tw] OR Majorca[tw] OR Menorca[tw] OR Minorca[tw] OR Murcia[tw] OR Ourense[tw] OR Orense[tw] OR Palencia[tw] OR Pontevedra[tw] OR Salamanca[tw] OR Segovia[tw] OR Sevilla[tw] OR Seville[tw] OR Soria[tw] OR Tarragona[tw] OR Tenerife[tw] OR Teruel[tw] OR Toledo[tw] OR Valencia[tw] OR Valladolid[tw] OR Vizcaya[tw] OR Biscay[tw] OR Zamora[tw] OR Zaragoza[tw] OR Saragossa[tw] OR Bilbao[tw] OR Bilbo[tw] OR Compostela[tw] OR "San Sebastian"[tw] OR Donostia[tw] OR Vitoria[tw] OR Oviedo[tw] OR Pamplona[tw] OR Logrono[tw] OR Gasteiz[tw])) OR (((Slovenia*[ad] OR Slovenija[ad] OR slovensk*[ad] OR Ljubljana[ad] OR Gorenjska[ad] OR Carniola[ad] OR Goriska[ad] OR Gorizia[ad] OR Koroska[ad] OR Carinthia[ad] OR "Notranjsko kraska"[ad] OR "Obalno kraska"[ad] OR "Coastal krast"[ad] OR Podravska[ad] OR Pomurska[ad] OR Savinjska[ad] OR Spodnjeposavska[ad] OR Zasavska[ad] OR Osrednjeslovenska[ad] OR Maribor[ad] OR Celje[ad] OR Kranj[ad] OR Velenje[ad] OR Koper[ad] OR Capodistria[ad] OR "Novo mesto"[ad] OR Ptuj[ad] OR Trbovlje[ad] OR Kamnik[ad] OR Murska[ad] OR Sobota[ad] OR "Nova Gorica"[ad])) OR (Slovenia*[tw] OR Slovenija[tw] OR slovensk*[tw] OR Ljubljana[tw] OR Gorenjska[tw] OR Carniola[tw] OR Goriska[tw] OR Gorizia[tw] OR Koroska[tw] OR Carinthia[tw] OR "Notranjsko kraska"[tw] OR "Obalno kraska"[tw] OR "Coastal krast"[tw] OR Podravska[tw] OR Pomurska[tw] OR Savinjska[tw] OR Spodnjeposavska[tw] OR Zasavska[tw] OR Osrednjeslovenska[tw] OR Maribor[tw] OR Celje[tw] OR Kranj[tw] OR Velenje[tw] OR Koper[tw] OR Capodistria[tw] OR "Novo mesto"[tw] OR Ptuj[tw] OR Trbovlje[tw] OR Kamnik[tw] OR Murska[tw] OR Sobota[tw] OR "Nova Gorica"[tw])) OR ((Slovakia[tw] OR Slovensk*[tw] OR Slovak*[tw] OR Bratislav*[tw] OR Trnav*[tw] OR Trnava[tw] OR Nitrian*[tw] OR Nitra[tw] OR Trencian*[tw] OR Trencin[tw] OR Banskobystri*[tw] OR "Banska Bystrica"[tw] OR Zilina[tw] OR Zilin*[tw] OR Trnava[tw] OR Trnav*[tw] OR Presov[tw] OR Presov*[tw] OR Kosic*[tw] OR (Martin[tw] AND (city[tw] OR Svaty[tw])) OR Poprad[tw])) OR (Slovakia[ad] OR Slovensk*[ad] OR Slovak*[ad] OR Bratislav*[ad] OR Trnav*[ad] OR Nitrian*[ad] OR Nitra[ad] OR Trencian*[ad] OR Trencin[ad] OR Banskobystri*[ad] OR "Banska Bystrica"[ad] OR Zilina[ad] OR Zilin*[ad] OR Trnava[ad] OR Trnav*[ad] OR Presov*[ad] OR Kosic*[ad] OR (Martin[ad] AND (city[ad] OR Svaty[ad])) OR Poprad[ad])) OR ((((Romania[tw] OR Rumania[tw] OR Roumania[tw] OR Romanian[tw] OR Roman[tw] OR Bucharest[tw] OR Bucuresti[tw] OR Alba[tw] OR Brasov[tw] OR Covasna[tw] OR Harghita[tw] OR Mures[tw] OR Sibiu[tw] OR Bacau[tw] OR Botosani[tw] OR Iasi[tw] OR Neamt[tw] OR Suceava[tw] OR Vaslui[tw] OR Bihor[tw] OR "Bistrita Nasaud"[tw] OR Cluj[tw] OR Maramures[tw] OR Salaj[tw] OR "Satu Mare"[tw] OR Arges[tw] OR Calarasi[tw] OR Dambovita[tw] OR Giurgiu[tw] OR Ialomita[tw] OR Prahova[tw] OR Teleorman[tw] OR Braila[tw] OR Buzau[tw] OR Constanta[tw] OR Galati[tw] OR Tulcea[tw] OR Vrancea[tw] OR Dolj[tw] OR Gorj[tw] OR Mehedinti[tw] OR (Olt[tw] AND (river[tw] OR county[tw] OR region[tw] OR judetul[tw] OR Raul[tw])) OR Valcea[tw] OR Vilcea[tw] OR Arad[tw] OR "Caras-Severin"[tw] OR Hunedoara[tw] OR Timis[tw] OR Ilfov[tw] OR Timisoara[tw] OR Constanta[tw] OR Craiova[tw] OR Ploiesti[tw] OR Oradea[tw] OR "Cluj-Napoca"[tw] OR Deva[tw])) OR (Romania[ad] OR Rumania[ad] OR Roumania[ad] OR Romanian[ad] OR Roman[ad] OR Bucharest[ad] OR Bucuresti[ad] OR Alba[ad] OR Brasov[ad] OR Covasna[ad] OR Harghita[ad] OR Mures[ad] OR Sibiu[ad] OR Bacau[ad] OR Botosani[ad] OR Iasi[ad] OR Neamt[ad] OR Suceava[ad] OR Vaslui[ad] OR Bihor[ad] OR "Bistrita Nasaud"[ad] OR Cluj[ad] OR Maramures[ad] OR Salaj[ad] OR "Satu Mare"[ad] OR Arges[ad] OR Calarasi[ad] OR Dambovita[ad] OR Giurgiu[ad] OR Ialomita[ad] OR Prahova[ad] OR Teleorman[ad] OR Braila[ad] OR Buzau[ad] OR Constanta[ad] OR Galati[ad] OR Tulcea[ad] OR Vrancea[ad] OR Dolj[ad] OR Gorj[ad] OR Mehedinti[ad] OR (Olt[ad] AND (river[ad] OR county[ad] OR region[ad] OR judetul[ad] OR Raul[ad])) OR Valcea[ad] OR Vilcea[ad] OR Arad[ad] OR "Caras-Severin"[ad] OR Hunedoara[ad] OR Timis[ad] OR Ilfov[ad] OR Timisoara[ad] OR Constanta[ad] OR Craiova[ad] OR Ploiesti[ad] OR Oradea[ad] OR "Cluj-Napoca"[ad] OR Deva[ad])) OR (((Portugal[tw] OR Portugues*[tw] OR Lisboa[tw] OR Lisbon[tw] OR Leira[tw] OR Santarem[tw] OR Beja[tw] OR Faro[tw] OR Evora[tw] OR Portalegre[tw] OR "Castelo Branco"[tw] OR Guarda[tw] OR Aveiro[tw] OR Viseu[tw] OR Braganca[tw] OR "Vila real"[tw] OR "Viana do Castelo"[tw] OR Alentejo[tw] OR Azores[tw] OR Acores[tw] OR Madeira[tw] OR "Os Montes"[tw] OR (Ave[tw] AND (community[tw] OR intermunicipal[tw] OR comunidade[tw])) OR Mondego[tw] OR Vouga[tw] OR Beira[tw] OR Cavado[tw] OR Lafoes[tw] OR Douro[tw] OR Porto[tw] OR Tejo[tw] OR Minho[tw] OR Setubal[tw] OR Pinhal[tw] OR "Serra da Estrela"[tw] OR Tamega[tw] OR Algarve[tw] OR Gaia[tw] OR Amadora[tw] OR Braga[tw] OR (Agualva[tw] AND Cacem[tw]) OR Funchal[tw] OR Coimbra[tw] OR Almada[tw])) OR (Portugal[ad] OR Portugues*[ad] OR Lisboa[ad] OR Lisbon[ad] OR Leira[ad] OR Santarem[ad] OR Beja[ad] OR Faro[ad] OR Evora[ad] OR Portalegre[ad] OR "Castelo Branco"[ad] OR Guarda[ad] OR Aveiro[ad] OR Viseu[ad] OR Braganca[ad] OR "Vila real"[ad] OR "Viana do Castelo"[ad] OR Alentejo[ad] OR Azores[ad] OR Acores[ad] OR Madeira[ad] OR "Os Montes"[ad] OR (Ave[ad] AND (community[ad] OR intermunicipal[ad] OR comunidade[ad])) OR Mondego[ad] OR Vouga[ad] OR Beira[ad] OR Cavado[ad] OR Lafoes[ad] OR Douro[ad] OR Porto[ad] OR Tejo[ad] OR Minho[ad] OR Setubal[ad] OR Pinhal[ad] OR "Serra da Estrela"[ad] OR Tamega[ad] OR Algarve[ad] OR Gaia[ad] OR Amadora[ad] OR Braga[ad] OR (Agualva[ad] AND Cacem[ad]) OR Funchal[ad] OR Coimbra[ad] OR Almada[ad])) OR (((Poland[ad] OR Polska[ad] OR Polish[ad] OR Polski[ad] OR Pole[ad] OR Poles[ad] OR Polak[ad] OR Polka[ad] OR Polacy[ad] OR Polacy[ad] OR Warsaw[ad] OR Warszawa[ad] OR Wielkopolskie[ad] OR Pomerania*[ad] OR Pomorskie[ad] OR Kuyavian[ad] OR Kujawsko[ad] OR Malopolskie[ad] OR Lodz[ad] OR Lodzkie[ad] OR Silesia*[ad] OR Slask[ad] OR Dolnoslaskie[ad] OR Lublin[ad] OR Lubelskie[ad] OR Lubus[ad] OR Lubusz[ad] OR Lubuskie[ad] OR Masovia[ad] OR Mazowske[ad] OR Masovian[ad] OR Mazowieckie[ad] OR Opole[ad] OR Opolskie[ad] OR Podlaskie[ad] OR Podlachia[ad] OR Podlasie[ad] OR Subcarpathian*[ad] OR Carpathian*[ad] OR Podkarpackie[ad] OR Swietokrzyskie[ad] OR Slaskie[ad] OR Slask[ad] OR "Varmia Mazuria"[ad] OR "Varmian Mazurian"[ad] OR "Varmia Masuria"[ad] OR "Varmian Masurian"[ad] OR "Warmia Mazury"[ad] OR "Warminsko Mazurskie"[ad] OR Zachodniopomorskie[ad] OR Krakow[ad] OR Cracow[ad] OR Wroclaw[ad] OR Poznan[ad] OR Gdansk[ad] OR Szczecin[ad] OR Bydgoszcz[ad] OR Katowice[ad] OR Bialystok[ad] OR Olsztyn[ad] OR Kielce[ad] OR "Zielona Gora"[ad] OR Torun[ad] OR "Gorzow Wielkopolski"[ad])) OR (Poland[tw] OR Polska[tw] OR Polish[tw] OR Polski[tw] OR Pole[tw] OR Poles[tw] OR Polak[tw] OR Polka[tw] OR Polacy[tw] OR Polacy[tw] OR Warsaw[tw] OR Warszawa[tw] OR Wielkopolskie[tw] OR Pomerania*[tw] OR Pomorskie[tw] OR Kuyavian[tw] OR Kujawsko[tw] OR Malopolskie[tw] OR Lodz[tw] OR Lodzkie[tw] OR Silesia*[tw] OR Slask[tw] OR Dolnoslaskie[tw] OR Lublin[tw] OR Lubelskie[tw] OR Lubus[tw] OR Lubusz[tw] OR Lubuskie[tw] OR Masovia[tw] OR Mazowske[tw] OR Masovian[tw] OR Mazowieckie[tw] OR Opole[tw] OR Opolskie[tw] OR Podlaskie[tw] OR Podlachia[tw] OR Podlasie[tw] OR Subcarpathian*[tw] OR Carpathian*[tw] OR Podkarpackie[tw] OR Swietokrzyskie[tw] OR Slaskie[tw] OR Slask[tw] OR "Varmia Mazuria"[tw] OR "Varmian Mazurian"[tw] OR "Varmia Masuria"[tw] OR "Varmian Masurian"[tw] OR "Warmia Mazury"[tw] OR "Warminsko Mazurskie"[tw] OR Zachodniopomorskie[tw] OR Krakow[tw] OR Cracow[tw] OR Wroclaw[tw] OR Poznan[tw] OR Gdansk[tw] OR Szczecin[tw] OR Bydgoszcz[tw] OR Katowice[tw] OR Bialystok[tw] OR Olsztyn[tw] OR Kielce[tw] OR "Zielona Gora"[tw] OR Torun[tw] OR "Gorzow Wielkopolski"[tw])) OR (((Netherlands[ad] OR Nederland*[ad] OR Dutch*[ad] OR Amsterdam[ad] OR Drenthe[ad] OR Flevoland[ad] OR Friesland[ad] OR Fryslan[ad] OR Gelderland[ad] OR Guelders[ad] OR Groningen[ad] OR Limburg[ad] OR "North Brabant"[ad] OR "Noord Brabant"[ad] OR Holland[ad] OR Overijssel[ad] OR Overissel[ad] OR Utrecht[ad] OR Zeeland[ad] OR Rotterdam[ad] OR Hague[ad] OR Eindhoven[ad] OR Tilburg[ad] OR Almere[ad] OR Breda[ad] OR Nijmegen[ad] OR Nimeguen[ad])) OR (Netherlands[tw] OR Nederland*[tw] OR Dutch*[tw] OR Amsterdam[tw] OR Drenthe[tw] OR Flevoland[tw] OR Friesland[tw] OR Fryslan[tw] OR Gelderland[tw] OR Guelders[tw] OR Groningen[tw] OR Limburg[tw] OR "North Brabant"[tw] OR "Noord Brabant"[tw] OR Holland[tw] OR Overijssel[tw] OR Overissel[tw] OR Utrecht[tw] OR Zeeland[tw] OR Rotterdam[tw] OR Hague[tw] OR Eindhoven[tw] OR Tilburg[tw] OR Almere[tw] OR Breda[tw] OR Nijmegen[tw] OR Nimeguen[tw])) OR (Malta[tw] OR Maltese[tw] OR Valletta[tw] OR Gozo[tw] OR Ghawdex[tw] OR Malta[ad] OR Maltese[ad] OR Valletta[ad] OR Gozo[ad] OR Ghawdex[ad]) OR (Luxembourg*[tw] OR Luxemburg[tw] OR Letzebuerg[tw] OR Diekirch[tw] OR Grevenmacher[tw] OR Luxembourg*[ad] OR Luxemburg[ad] OR Letzebuerg[ad] OR Diekirch[ad] OR Grevenmacher[ad]) OR (((Lithuania*[tw] OR "Lietuvos Respublika"[tw] OR Lietuva[tw] OR lietuviu[tw] OR Vilnius[tw] OR Vilniaus[tw] OR Kaunas[tw] OR Kauno[tw] OR Klaipeda[tw] OR Klaipedos[tw] OR Panevezys[tw] OR Panevezio[tw] OR Siauliai[tw] OR Siauliu[tw] OR Alytus[tw] OR Alytaus[tw] OR Taurages[tw] OR Taurage[tw] OR Marijampoles[tw] OR Marijampole[tw] OR Telsiu[tw] OR Telsiai[tw] OR Utenos[tw] OR Utena[tw] OR Mazeikiai[tw] OR Jonava[tw] OR Mazeikiu[tw] OR Jonavos[tw])) OR (Lithuania*[ad] OR "Lietuvos Respublika"[ad] OR Lietuva[ad] OR lietuviu[ad] OR Vilnius[ad] OR Vilniaus[ad] OR Kaunas[ad] OR Kauno[ad] OR Klaipeda[ad] OR Klaipedos[ad] OR Panevezys[ad] OR Panevezio[ad] OR Siauliai[ad] OR Siauliu[ad] OR Alytus[ad] OR Alytaus[ad] OR Taurages[ad] OR Taurage[ad] OR Marijampoles[ad] OR Marijampole[ad] OR Telsiu[ad] OR Telsiai[ad] OR Utenos[ad] OR Utena[ad] OR Mazeikiai[ad] OR Jonava[ad] OR Mazeikiu[ad] OR Jonavos[ad])) OR (((Latvi*[tw] OR Latvija*[tw] OR Riga[tw] OR Courland[tw] OR Kurzeme[tw] OR Kurland[tw] OR Latgale[tw] OR Lettgallia[tw] OR Latgola[tw] OR Vidzeme[tw] OR Vidumo[tw] OR Semigallia[tw] OR Semigalia[tw] OR Zemgale[tw] OR Pieriga[tw] OR Daugavpils[tw] OR Dinaburg[tw] OR Liepaja[tw] OR Libau[tw] OR Jelgava[tw] OR Jurmala[tw] OR Jekabpils[tw] OR Jakobstadt[tw] OR Rezekne[tw] OR Rezne[tw] OR Rositten[tw] OR Valmiera[tw] OR Wolmar[tw] OR Ventspils[tw] OR Windau[tw])) OR (Latvi*[ad] OR Latvija*[ad] OR Riga[ad] OR Courland[ad] OR Kurzeme[ad] OR Kurland[ad] OR Latgale[ad] OR Lettgallia[ad] OR Latgola[ad] OR Vidzeme[ad] OR Vidumo[ad] OR Semigallia[ad] OR Semigalia[ad] OR Zemgale[ad] OR Pieriga[ad] OR Daugavpils[ad] OR Dinaburg[ad] OR Liepaja[ad] OR Libau[ad] OR Jelgava[ad] OR Jurmala[ad] OR Jekabpils[ad] OR Jakobstadt[ad] OR Rezekne[ad] OR Rezne[ad] OR Rositten[ad] OR Valmiera[ad] OR Wolmar[ad] OR Ventspils[ad] OR Windau[ad]))) OR ((((Italy[tw] OR Italia*[tw] OR Rome[tw] OR Roma[tw] OR Abruzzo[tw] OR Abruzzi[tw] OR Basilicata[tw] OR Lucania[tw] OR Calabria[tw] OR Campania[tw] OR "Emilia Romagna"[tw] OR "friuli venezia giulia"[tw] OR Lazio[tw] OR Latium[tw] OR Liguria*[tw] OR Lombardy[tw] OR Lombardia[tw] OR Marche[tw] OR Marches[tw] OR Molisano[tw] OR Molise[tw] OR Piedmont*[tw] OR Piemonte[tw] OR Sardinia[tw] OR Sardegna[tw] OR Sicily[tw] OR Sicilia[tw] OR Toscana[tw] OR Tuscany[tw] OR Trentino[tw] OR Trento[tw] OR Umbria[tw] OR Veneto[tw] OR Triveneto[tw] OR Puglia[tw] OR Apulia[tw] OR Bolzano[tw] OR Bozen[tw] OR Milan[tw] OR Milano[tw] OR Naples[tw] OR Napoli[tw] OR Turin[tw] OR Torino[tw] OR Palermo[tw] OR Genoa[tw] OR Genova[tw] OR Bologna[tw] OR Florence[tw] OR Firenze[tw] OR Bari[tw] OR Catania[tw] OR Venezia[tw] OR Venice[tw] OR Padova[tw] OR Padua[tw] OR Siena[tw] OR Bologna[tw] OR Trieste[tw] OR Urbino[tw] OR Aosta[tw] OR Aoste[tw] OR Perugia[tw] OR Brescia[tw] OR Cagliari[tw] OR Catanzaro[tw] OR "L Aquila"[tw] OR Ancona[tw])) OR (Italy[ad] OR Italia*[ad] OR Rome[ad] OR Roma[ad] OR Abruzzo[ad] OR Abruzzi[ad] OR Basilicata[ad] OR Lucania[ad] OR Calabria[ad] OR Campania[ad] OR "Emilia Romagna"[ad] OR "friuli venezia giulia"[ad] OR Lazio[ad] OR Latium[ad] OR Liguria*[ad] OR Lombardy[ad] OR Lombardia[ad] OR Marche[ad] OR Marches[ad] OR Molisano[ad] OR Molise[ad] OR Piedmont*[ad] OR Piemonte[ad] OR Sardinia[ad] OR Sardegna[ad] OR Sicily[ad] OR Sicilia[ad] OR Toscana[ad] OR Tuscany[ad] OR Trentino[ad] OR Trento[ad] OR Umbria[ad] OR Veneto[ad] OR Triveneto[ad] OR Puglia[ad] OR Apulia[ad] OR Bolzano[ad] OR Bozen[ad] OR Milan[ad] OR Milano[ad] OR Naples[ad] OR Napoli[ad] OR Turin[ad] OR Torino[ad] OR Palermo[ad] OR Genoa[ad] OR Genova[ad] OR Bologna[ad] OR Florence[ad] OR Firenze[ad] OR Bari[ad] OR Catania[ad] OR Venezia[ad] OR Venice[ad] OR Padova[ad] OR Padua[ad] OR Siena[ad] OR Bologna[ad] OR Trieste[ad] OR Urbino[ad] OR Aosta[ad] OR Aoste[ad] OR Perugia[ad] OR Brescia[ad] OR Cagliari[ad] OR Catanzaro[ad] OR "L Aquila"[ad] OR Ancona[ad])) OR (((Ireland[tw] OR Eire[tw] OR Irish*[tw] OR Dublin[tw] OR Fingal[tw] OR "Dun Laoghaire"[tw] OR Wicklow[tw] OR Wexford[tw] OR Carlow[tw] OR Kildare[tw] OR Meath[tw] OR Louth[tw] OR Monaghan[tw] OR Cavan[tw] OR Longford[tw] OR Westmeath[tw] OR Offaly[tw] OR Laois[tw] OR Kilkenny[tw] OR Waterford[tw] OR Cork[tw] OR Kerry[tw] OR Limerick[tw] OR Tipperary[tw] OR Clare[tw] OR Galway[tw] OR Mayo[tw] OR Roscommon[tw] OR Sligo[tw] OR Leitrim[tw] OR Donegal[tw] OR Drogheda[tw] OR Dundalk[tw] OR Swords[tw] OR Bray[tw] OR Navan[tw] OR Munster[tw] OR Leinster[tw] OR Connacht[tw])) OR (Ireland[ad] OR Eire[ad] OR Irish*[ad] OR Dublin[ad] OR Fingal[ad] OR "Dun Laoghaire"[ad] OR Wicklow[ad] OR Wexford[ad] OR Carlow[ad] OR Kildare[ad] OR Meath[ad] OR Louth[ad] OR Monaghan[ad] OR Cavan[ad] OR Longford[ad] OR Westmeath[ad] OR Offaly[ad] OR Laois[ad] OR Kilkenny[ad] OR Waterford[ad] OR Cork[ad] OR Kerry[ad] OR Limerick[ad] OR Tipperary[ad] OR Clare[ad] OR Galway[ad] OR Mayo[ad] OR Roscommon[ad] OR Sligo[ad] OR Leitrim[ad] OR Donegal[ad] OR Drogheda[ad] OR Dundalk[ad] OR Swords[ad] OR Bray[ad] OR Navan[ad] OR Munster[ad] OR Leinster[ad] OR Connacht[ad])) OR (((Hungar*[tw] OR Budapest[tw] OR Transdanubia[tw] OR Magyarorszag[tw] OR magyar[tw] OR Dunantuli[tw] OR Dunantul[tw] OR "Great Plain"[tw] OR "Alfold es eszak"[tw] OR "Eszak Alfold"[tw] OR "Del Alfold"[tw] OR Bacs[tw] OR Kiskun[tw] OR "Northen Alfold"[tw] OR "Sourthen Alfold"[tw] OR Baranya[tw] OR Bekes[tw] OR borsod[tw] OR Abauj[tw] OR Zemplen[tw] OR Fovaros[tw] OR Csongrad[tw] OR Fejer[tw] OR gyor[tw] OR moson[tw] OR sopron[tw] OR hajdu[tw] OR bihar[tw] OR Heves[tw] OR "jasz nagykun szolnok"[tw] OR komarom[tw] OR esztergom[tw] OR Nograd[tw] OR Pest[tw] OR Somogy[tw] OR szabolcs[tw] OR szatmar[tw] OR bereg[tw] OR Tolna[tw] OR Vas[tw] OR Veszprem[tw] OR Zala[tw] OR Zalaegerszeg[tw] OR Debrecen[tw] OR Miskolc[tw] OR Szeged[tw] OR Pecs[tw] OR Gyor[tw] OR Nyiregyhaza[tw] OR Kecskemet[tw] OR Szekesfehervar[tw] OR Szombathely[tw] OR Bekescsaba[tw] OR Eger[tw] OR Tatabanya[tw] OR Salgotarjan[tw] OR Kaposvar[tw] OR Szekszard[tw])) OR (Hungar*[ad] OR Budapest[ad] OR Transdanubia[ad] OR Magyarorszag[ad] OR magyar[ad] OR Dunantuli[ad] OR Dunantul[ad] OR "Great Plain"[ad] OR "Alfold es eszak"[ad] OR "Eszak Alfold"[ad] OR "Del Alfold"[ad] OR Bacs[ad] OR Kiskun[ad] OR "Northen Alfold"[ad] OR "Sourthen Alfold"[ad] OR Baranya[ad] OR Bekes[ad] OR borsod[ad] OR Abauj[ad] OR Zemplen[ad] OR Fovaros[ad] OR Csongrad[ad] OR Fejer[ad] OR gyor[ad] OR moson[ad] OR sopron[ad] OR hajdu[ad] OR bihar[ad] OR Heves[ad] OR "jasz nagykun szolnok"[ad] OR komarom[ad] OR esztergom[ad] OR Nograd[ad] OR Pest[ad] OR Somogy[ad] OR szabolcs[ad] OR szatmar[ad] OR bereg[ad] OR Tolna[ad] OR Vas[ad] OR Veszprem[ad] OR Zala[ad] OR Zalaegerszeg[ad] OR Debrecen[ad] OR Miskolc[ad] OR Szeged[ad] OR Pecs[ad] OR Gyor[ad] OR Nyiregyhaza[ad] OR Kecskemet[ad] OR Szekesfehervar[ad] OR Szombathely[ad] OR Bekescsaba[ad] OR Eger[ad] OR Tatabanya[ad] OR Salgotarjan[ad] OR Kaposvar[ad] OR Szekszard[ad])) OR (((Greece[ad] OR "Hellenic republic"[ad] OR Greek*[ad] OR Ellada[ad] OR "Elliniki Dimokratia"[ad] OR Hellas[ad] OR Hellenes[ad] OR Attica[ad] OR Attiki[ad] OR Makedonia[ad] OR Macedonia[ad] OR Thraki[ad] OR Thrace[ad] OR Crete[ad] OR Kriti[ad] OR Epirus[ad] OR Ipeiros[ad] OR "Ionia Nisia"[ad] OR "Ionion neson"[ad] OR "Ionian islands"[ad] OR "North aegean"[ad] OR "Aegean islands"[ad] OR "Nisoi Agaiou"[ad] OR "Notio Aigaio"[ad] OR Peloponnese[ad] OR Peloponnisos[ad] OR "Voreio Aigaio"[ad] OR "South aegean"[ad] OR Thessaly[ad] OR Thessalia[ad] OR Cycklades[ad] OR Kiklades[ad] OR Dodecanese[ad] OR Dodekanisa[ad] OR "Mount athos"[ad] OR "Omicronros Alphathos"[ad] OR Athens[ad] OR Athina[ad] OR Thessaloniki[ad] OR Thessalonica[ad] OR Patras[ad] OR Patra[ad] OR Heraklion[ad] OR Heraclion[ad] OR Iraklion[ad] OR Irakleion[ad] OR Iraklio[ad] OR Larissa[ad] OR Larisa[ad] OR Volos[ad] OR Rhodes[ad] OR Rodos[ad] OR Ioannina[ad] OR Janina[ad] OR Yannena[ad] OR Chania[ad] OR Chalcis[ad] OR Chalkida[ad] OR Alexandroupoli[ad])) OR (Greece[tw] OR "Hellenic republic"[tw] OR Greek*[tw] OR Ellada[tw] OR "Elliniki Dimokratia"[tw] OR Hellas[tw] OR Hellenes[tw] OR Attica[tw] OR Attiki[tw] OR Makedonia[tw] OR Macedonia[tw] OR Thraki[tw] OR Thrace[tw] OR Crete[tw] OR Kriti[tw] OR Epirus[tw] OR Ipeiros[tw] OR "Ionia Nisia"[tw] OR "Ionion neson"[tw] OR "Ionian islands"[tw] OR "North aegean"[tw] OR "Aegean islands"[tw] OR "Nisoi Agaiou"[tw] OR "Notio Aigaio"[tw] OR Peloponnese[tw] OR Peloponnisos[tw] OR "Voreio Aigaio"[tw] OR "South aegean"[tw] OR Thessaly[tw] OR Thessalia[tw] OR Cycklades[tw] OR Kiklades[tw] OR Dodecanese[tw] OR Dodekanisa[tw] OR "Mount athos"[tw] OR "Omicronros Alphathos"[tw] OR Athens[tw] OR Athina[tw] OR Thessaloniki[tw] OR Thessalonica[tw] OR Patras[tw] OR Patra[tw] OR Heraklion[tw] OR Heraclion[tw] OR Iraklion[tw] OR Irakleion[tw] OR Iraklio[tw] OR Larissa[tw] OR Larisa[tw] OR Volos[tw] OR Rhodes[tw] OR Rodos[tw] OR Ioannina[tw] OR Janina[tw] OR Yannena[tw] OR Chania[tw] OR Chalcis[tw] OR Chalkida[tw] OR Alexandroupoli[tw])) OR (((German*[ad] OR Deutschland[ad] OR Deutsch*[ad] OR Bundesrepublik[ad] OR Westdeutschland[ad] OR Ostdeutschland[ad] OR Baden[ad] OR Wuerttemberg[ad] OR Wurttemberg[ad] OR Bayern[ad] OR Bavaria[ad] OR Berlin[ad] OR Brandenburg[ad] OR Bremen[ad] OR Hamburg[ad] OR Hessen[ad] OR Hesse[ad] OR Hessia[ad] OR Mecklenburg[ad] OR Vorpommern[ad] OR Pomerania[ad] OR Niedersachsen[ad] OR Neddersassen[ad] OR Saxony[ad] OR Niederbayern[ad] OR "North Rhine"[ad] OR Westphalia[ad] OR Westfalen[ad] OR "Rhineland Palatinate"[ad] OR "Rheinland Pfalz"[ad] OR Saarland[ad] OR Sachsen[ad] OR "Schleswig Holstein"[ad] OR Thuringia[ad] OR Thuringen[ad] OR Thueringen[ad] OR Freiburg[ad] OR Karlsruhe[ad] OR Calsruhe[ad] OR Stuttgart[ad] OR Tubingen[ad] OR Oberbayern[ad] OR "Upper palatinate"[ad] OR Oberpfalz[ad] OR Franken[ad] OR Franconia[ad] OR Oberfranken[ad] OR Mittelfranken[ad] OR Schwaben[ad] OR Unterfranken[ad] OR Swabia[ad] OR Darmstadt[ad] OR Giessen[ad] OR Giessen[ad] OR Kassel[ad] OR Arnsberg[ad] OR Cologne[ad] OR Koln[ad] OR Koeln[ad] OR Detmold[ad] OR Dusseldorf[ad] OR Duesseldorf[ad] OR Munster[ad] OR Muenster[ad] OR Munich[ad] OR Munchen[ad] OR Muenchen[ad] OR Frankfurt[ad] OR Dortmund[ad] OR Essen[ad] OR Nurnberg[ad] OR Nuernberg[ad] OR Nuremberg[ad] OR Hanover[ad] OR Hannover[ad] OR Leipzig[ad] OR Dresden[ad] OR Ruhrgebiet[ad] OR Revier[ad] OR Ruhrpott[ad] OR Pott[ad] OR Ruhr[ad])) OR (German*[tw] OR Deutschland[tw] OR Deutsch*[tw] OR Bundesrepublik[tw] OR Westdeutschland[tw] OR Ostdeutschland[tw] OR Baden[tw] OR Wuerttemberg[tw] OR Wurttemberg[tw] OR Bayern[tw] OR Bavaria[tw] OR Berlin[tw] OR Brandenburg[tw] OR Bremen[tw] OR Hamburg[tw] OR Hessen[tw] OR Hesse[tw] OR Hessia[tw] OR Mecklenburg[tw] OR Vorpommern[tw] OR Pomerania[tw] OR Niedersachsen[tw] OR Neddersassen[tw] OR Saxony[tw] OR Niederbayern[tw] OR "North Rhine"[tw] OR Westphalia[tw] OR Westfalen[tw] OR "Rhineland Palatinate"[tw] OR "Rheinland Pfalz"[tw] OR Saarland[tw] OR Sachsen[tw] OR "Schleswig Holstein"[tw] OR Thuringia[tw] OR Thuringen[tw] OR Thueringen[tw] OR Freiburg[tw] OR Karlsruhe[tw] OR Calsruhe[tw] OR Stuttgart[tw] OR Tubingen[tw] OR Oberbayern[tw] OR "Upper palatinate"[tw] OR Oberpfalz[tw] OR Franken[tw] OR Franconia[tw] OR Oberfranken[tw] OR Mittelfranken[tw] OR Schwaben[tw] OR Unterfranken[tw] OR Swabia[tw] OR Darmstadt[tw] OR Giessen[tw] OR Giessen[tw] OR Kassel[tw] OR Arnsberg[tw] OR Cologne[tw] OR Koln[tw] OR Koeln[tw] OR Detmold[tw] OR Dusseldorf[tw] OR Duesseldorf[tw] OR Munster[tw] OR Muenster[tw] OR Munich[tw] OR Munchen[tw] OR Muenchen[tw] OR Frankfurt[tw] OR Dortmund[tw] OR Essen[tw] OR Nurnberg[tw] OR Nuernberg[tw] OR Nuremberg[tw] OR Hanover[tw] OR Hannover[tw] OR Leipzig[tw] OR Dresden[tw] OR Ruhrgebiet[tw] OR Revier[tw] OR Ruhrpott[tw] OR Pott[tw] OR Ruhr[tw])) OR (((France[ad] OR French*[ad] OR Francais[ad] OR Alsace[ad] OR Aquitaine[ad] OR Auvergne[ad] OR Brittany[ad] OR Bretagne[ad] OR Bourgogne[ad] OR Burgundy[ad] OR "Champagne Ardenne"[ad] OR "Franche Comte"[ad] OR "Ile de France"[ad] OR "Languedoc Roussillon"[ad] OR Limousin[ad] OR Lorraine[ad] OR Normandie[ad] OR Normandy[ad] OR "Midi Pyrenees"[ad] OR "Nord Pas de Calais"[ad] OR Loire[ad] OR Picardie[ad] OR Picardy[ad] OR "Poitou Charentes"[ad] OR Provence[ad] OR "Rhone Alpes"[ad] OR Corse[ad] OR Corsica[ad] OR Guiana[ad] OR Guyane[ad] OR Guadeloupe[ad] OR Martinique[ad] OR Reunion[ad] OR Mayotte[ad] OR Ain[ad] OR Aisne[ad] OR Allier[ad] OR "Alpes de Haute Provence"[ad] OR "Haute Alpes"[ad] OR "Alpes Maritimes"[ad] OR Ardeche[ad] OR Ardennes[ad] OR Ariege[ad] OR Aube[ad] OR Aude[ad] OR Aveyron[ad] OR "Bas Rhin"[ad] OR "Bouches du Rhone"[ad] OR Calvados[ad] OR Cantal[ad] OR Charente[ad] OR Cher[ad] OR Correze[ad] OR "Corse du Sud"[ad] OR "Cote d Or"[ad] OR "Cotes d Armor"[ad] OR "Cote d Azur"[ad] OR Creuse[ad] OR "Deux Sevres"[ad] OR Dordogne[ad] OR Doubs[ad] OR Drome[ad] OR Essonne[ad] OR Eure[ad] OR Finistere[ad] OR Gard[ad] OR Gers[ad] OR Gironde[ad] OR "Haute Corse"[ad] OR "Haute Garonne"[ad] OR "Haute Marne"[ad] OR "Hautes Alpes"[ad] OR "Haute Saone"[ad] OR "Haute Savoie"[ad] OR "Hautes Pyrenees"[ad] OR "Haute Vienne"[ad] OR "Haut Rhin"[ad] OR "Hauts de Seine"[ad] OR Herault[ad] OR "Ille et Vilaine"[ad] OR Indre[ad] OR Isere[ad] OR Jura[ad] OR Landes[ad] OR Loire[ad] OR Loiret[ad] OR (Lot[ad] AND (departement[ad] OR department[ad])) OR "Lot et Garonne"[ad] OR "Loir et Cher"[ad] OR Lozere[ad] OR Manche[ad] OR Marne[ad] OR Mayenne[ad] OR "Meurthe et Moselle"[ad] OR Meuse[ad] OR Morbihan[ad] OR Moselle[ad] OR (Nord[ad] AND (department[ad] OR departement[ad])) OR Nievre[ad] OR Oise[ad] OR Orne[ad] OR "Pas de calais"[ad] OR Paris[ad] OR "Puy de dome"[ad] OR "Pyrenees Atlantiques"[ad] OR "Pyrenees Orientales"[ad] OR Rhone[ad] OR Sarthe[ad] OR Savoie[ad] OR "Seine et Marne"[ad] OR "Seine Maritime"[ad] OR Somme[ad] OR Tarn[ad] OR "Territoire de Belfort"[ad] OR "Val de Marne"[ad] OR "Val d Oise"[ad] OR Var[ad] OR Vaucluse[ad] OR Vendee[ad] OR Vienne[ad] OR Vosges[ad] OR Yonne[ad] OR Yvelines[ad] OR Marseille[ad] OR Lyon[ad] OR Nice[ad] OR Nantes[ad] OR Strasbourg[ad] OR Montpellier[ad] OR Bordeaux[ad] OR Lille[ad] OR Toulouse[ad])) OR (France[tw] OR French*[tw] OR Francais[tw] OR Alsace[tw] OR Aquitaine[tw] OR Auvergne[tw] OR Brittany[tw] OR Bretagne[tw] OR Bourgogne[tw] OR Burgundy[tw] OR "Champagne Ardenne"[tw] OR "Franche Comte"[tw] OR "Ile de France"[tw] OR "Languedoc Roussillon"[tw] OR Limousin[tw] OR Lorraine[tw] OR Normandie[tw] OR Normandy[tw] OR "Midi Pyrenees"[tw] OR "Nord Pas de Calais"[tw] OR Loire[tw] OR Picardie[tw] OR Picardy[tw] OR "Poitou Charentes"[tw] OR Provence[tw] OR "Rhone Alpes"[tw] OR Corse[tw] OR Corsica[tw] OR Guiana[tw] OR Guyane[tw] OR Guadeloupe[tw] OR Martinique[tw] OR Reunion[tw] OR Mayotte[tw] OR Ain[tw] OR Aisne[tw] OR Allier[tw] OR "Alpes de Haute Provence"[tw] OR "Haute Alpes"[tw] OR "Alpes Maritimes"[tw] OR Ardeche[tw] OR Ardennes[tw] OR Ariege[tw] OR Aube[tw] OR Aude[tw] OR Aveyron[tw] OR "Bas Rhin"[tw] OR "Bouches du Rhone"[tw] OR Calvados[tw] OR Cantal[tw] OR Charente[tw] OR Cher[tw] OR Correze[tw] OR "Corse du Sud"[tw] OR "Cote d Or"[tw] OR "Cotes d Armor"[tw] OR "Cote d Azur"[tw] OR Creuse[tw] OR "Deux Sevres"[tw] OR Dordogne[tw] OR Doubs[tw] OR Drome[tw] OR Essonne[tw] OR Eure[tw] OR Finistere[tw] OR Gard[tw] OR Gers[tw] OR Gironde[tw] OR "Haute Corse"[tw] OR "Haute Garonne"[tw] OR "Haute Marne"[tw] OR "Hautes Alpes"[tw] OR "Haute Saone"[tw] OR "Haute Savoie"[tw] OR "Hautes Pyrenees"[tw] OR "Haute Vienne"[tw] OR "Haut Rhin"[tw] OR "Hauts de Seine"[tw] OR Herault[tw] OR "Ille et Vilaine"[tw] OR Indre[tw] OR Isere[tw] OR Jura[tw] OR Landes[tw] OR Loire[tw] OR Loiret[tw] OR (Lot[tw] AND (departement[tw] OR department[tw])) OR "Lot et Garonne"[tw] OR "Loir et Cher"[tw] OR Lozere[tw] OR Manche[tw] OR Marne[tw] OR Mayenne[tw] OR "Meurthe et Moselle"[tw] OR Meuse[tw] OR Morbihan[tw] OR Moselle[tw] OR (Nord[tw] AND (department[tw] OR departement[tw])) OR Nievre[tw] OR Oise[tw] OR Orne[tw] OR "Pas de calais"[tw] OR Paris[tw] OR "Puy de dome"[tw] OR "Pyrenees Atlantiques"[tw] OR "Pyrenees Orientales"[tw] OR Rhone[tw] OR Sarthe[tw] OR Savoie[tw] OR "Seine et Marne"[tw] OR "Seine Maritime"[tw] OR Somme[tw] OR Tarn[tw] OR "Territoire de Belfort"[tw] OR "Val de Marne"[tw] OR "Val d Oise"[tw] OR Var[tw] OR Vaucluse[tw] OR Vendee[tw] OR Vienne[tw] OR Vosges[tw] OR Yonne[tw] OR Yvelines[tw] OR Marseille[tw] OR Lyon[tw] OR Nice[tw] OR Nantes[tw] OR Strasbourg[tw] OR Montpellier[tw] OR Bordeaux[tw] OR Lille[tw] OR Toulouse[tw])) OR (((Finland[tw] OR Finnish*[tw] OR Suomi*[tw] OR Lapland[tw] OR Lappi[tw] OR Lappland[tw] OR Ostrobothnia[tw] OR Pohjanmaa[tw] OR Osterbotten[tw] OR Kainuu[tw] OR Kajanaland*[tw] OR Karelia[tw] OR Karjala[tw] OR Karelen[tw] OR Savonia[tw] OR Savo[tw] OR Savolax[tw] OR Pirkanmaa[tw] OR Birkaland[tw] OR Satakunta[tw] OR Satakunda[tw] OR Tavastia[tw] OR Tavastland[tw] OR "Paijat Hame"[tw] OR "Kanta Hame"[tw] OR Uusimaa[tw] OR Nyland[tw] OR Kymenlaakso[tw] OR Kymmenedalen[tw] OR Aland[tw] OR Ahvenanmaa[tw] OR Helsinki[tw] OR Helsingfors[tw] OR Espoo[tw] OR Esbo[tw] OR Tampere[tw] OR Tammerfors[tw] OR Vantaa[tw] OR Vanda[tw] OR Oulu[tw] OR Uleaborg[tw] OR Turku[tw] OR Abo[tw] OR Jyvaskyla[tw] OR Kuopio[tw] OR Lathi[tw] OR Lahtis[tw] OR Kouvola[tw])) OR (Finland[ad] OR Finnish*[ad] OR Suomi*[ad] OR Lapland[ad] OR Lappi[ad] OR Lappland[ad] OR Ostrobothnia[ad] OR Pohjanmaa[ad] OR Osterbotten[ad] OR Kainuu[ad] OR Kajanaland*[ad] OR Karelia[ad] OR Karjala[ad] OR Karelen[ad] OR Savonia[ad] OR Savo[ad] OR Savolax[ad] OR Pirkanmaa[ad] OR Birkaland[ad] OR Satakunta[ad] OR Satakunda[ad] OR Tavastia[ad] OR Tavastland[ad] OR "Paijat Hame"[ad] OR "Kanta Hame"[ad] OR Uusimaa[ad] OR Nyland[ad] OR Kymenlaakso[ad] OR Kymmenedalen[ad] OR Aland[ad] OR Ahvenanmaa[ad] OR Helsinki[ad] OR Helsingfors[ad] OR Espoo[ad] OR Esbo[ad] OR Tampere[ad] OR Tammerfors[ad] OR Vantaa[ad] OR Vanda[ad] OR Oulu[ad] OR Uleaborg[ad] OR Turku[ad] OR Abo[ad] OR Jyvaskyla[ad] OR Kuopio[ad] OR Lathi[ad] OR Lahtis[ad] OR Kouvola[ad])) OR (((Estonia*[tw] OR Eesti[tw] OR Esti[tw] OR Tallinn[tw] OR Harju[tw] OR Harjumaa[tw] OR Hiiu[tw] OR Hiiumaa[tw] OR "Ida Viru"[tw] OR "Ida Virumaa"[tw] OR Jarvamaa[tw] OR Jarva[tw] OR Jogevamaa[tw] OR Jogeva[tw] OR Laanemma[tw] OR Laane[tw] OR Parnu[tw] OR Parnumaa[tw] OR Polva[tw] OR Polvamaa[tw] OR Rapla[tw] OR Raplamaa[tw] OR Saare[tw] OR Saaremaa[tw] OR Tartu[tw] OR Tartumaa[tw] OR Valga[tw] OR Valgamaa[tw] OR Valgamaakond[tw] OR Viljandimaa[tw] OR Viljandi[tw] OR Voru[tw] OR Vorumaa[tw] OR Narva[tw] OR Parnu[tw] OR "Kohtla Jarve"[tw] OR Viljandi[tw] OR Rakvere[tw] OR Maardu[tw] OR Sillamae[tw] OR Kuressaare[tw])) OR (Estonia*[ad] OR Eesti[ad] OR Esti[ad] OR Tallinn[ad] OR Harju[ad] OR Harjumaa[ad] OR Hiiu[ad] OR Hiiumaa[ad] OR "Ida Viru"[ad] OR "Ida Virumaa"[ad] OR Jarvamaa[ad] OR Jarva[ad] OR Jogevamaa[ad] OR Jogeva[ad] OR Laanemma[ad] OR Laane[ad] OR Parnu[ad] OR Parnumaa[ad] OR Polva[ad] OR Polvamaa[ad] OR Rapla[ad] OR Raplamaa[ad] OR Saare[ad] OR Saaremaa[ad] OR Tartu[ad] OR Tartumaa[ad] OR Valga[ad] OR Valgamaa[ad] OR Valgamaakond[ad] OR Viljandimaa[ad] OR Viljandi[ad] OR Voru[ad] OR Vorumaa[ad] OR Narva[ad] OR Parnu[ad] OR "Kohtla Jarve"[ad] OR Viljandi[ad] OR Rakvere[ad] OR Maardu[ad] OR Sillamae[ad] OR Kuressaare[ad]))) OR ((((Denmark[tw] OR Danish*[tw] OR Danmark[tw] OR dansk*[tw] OR Hovedstaden[tw] OR Midtjylland[tw] OR Nordjylland[tw] OR Sjaelland[tw] OR Sealand[tw] OR Syddanmark[tw] OR Jutland[tw] OR Jylland[tw] OR Nordjylland[tw] OR Sonderjyllands[tw] OR "Zealand region"[tw] OR "region Zealand"[tw] OR Hillerod[tw] OR Viborg[tw] OR Aalborg[tw] OR Alborg[tw] OR Soro[tw] OR Vejle[tw] OR Copenhagen[tw] OR Kobenhavn[tw] OR Arhus[tw] OR Aarhus[tw] OR Roskilde[tw] OR Odense[tw] OR Frederiksberg[tw] OR Esbjerg[tw] OR Gentofte[tw] OR Gladsaxe[tw] OR Randers[tw] OR Kolding[tw])) OR (Denmark[ad] OR Danish*[ad] OR Danmark[ad] OR dansk*[ad] OR Hovedstaden[ad] OR Midtjylland[ad] OR Nordjylland[ad] OR Sjaelland[ad] OR Sealand[ad] OR Syddanmark[ad] OR Jutland[ad] OR Jylland[ad] OR Nordjylland[ad] OR Sonderjyllands[ad] OR "Zealand region"[ad] OR "region Zealand"[ad] OR Hillerod[ad] OR Viborg[ad] OR Aalborg[ad] OR Alborg[ad] OR Soro[ad] OR Vejle[ad] OR Copenhagen[ad] OR Kobenhavn[ad] OR Arhus[ad] OR Aarhus[ad] OR Roskilde[ad] OR Odense[ad] OR Frederiksberg[ad] OR Esbjerg[ad] OR Gentofte[ad] OR Gladsaxe[ad] OR Randers[ad] OR Kolding[ad])) OR ((Czech*[tw] OR Cesk*[tw] OR Stredoces*[tw] OR Jihoces*[tw] OR Bohemia[tw] OR "Bohemian region"[tw] OR Kralovehradec*[tw] OR "Hradec Kralove"[tw] OR Karlovars*[tw] OR "Karlovy Vary"[tw] OR Liberec*[tw] OR Moravskoslezs*[tw] OR "Moravian Silesian"[tw] OR Olomouc*[tw] OR Pardubic*[tw] OR Plzen*[tw] OR Pilsen[tw] OR Prage[tw] OR Praha[tw] OR Prag[tw] OR Jihomorav*[tw] OR Moravia[tw] OR Moravian[tw] OR Morava[tw] OR Usteck*[tw] OR Usti[tw] OR Vysocina[tw] OR Zlin[tw] OR Zlinsk*[tw] OR "Ceske Budejovice"[tw] OR Budweis[tw] OR Brno[tw] OR Ostrava[tw])) OR (Czech*[ad] OR Cesk*[ad] OR Stredoces*[ad] OR Jihoce*[ad] OR Bohemia[ad] OR "Bohemian region"[ad] OR Kralovehradec*[ad] OR "Hradec Kralove"[ad] OR Karlovars*[ad] OR "Karlovy Vary"[ad] OR Liberec*[ad] OR Moravskoslezsk*[ad] OR "Moravian Silesian"[ad] OR Olomouc*[ad] OR Pardubic*[ad] OR Pardubice[ad] OR Plzen*[ad] OR Pilsen[ad] OR Prage[ad] OR Praha[ad] OR Prag[ad] OR Jihomorav*[ad] OR Moravia[ad] OR Moravian[ad] OR Morava[ad] OR Usteck*[ad] OR Usti[ad] OR Vysocina[ad] OR Zlin[ad] OR Zlinsk*[ad] OR "Ceske Budejovice"[ad] OR Budweis[ad] OR Brno[ad] OR Ostrava[ad]) OR (((Cyprus[tw] OR Cypriot*[tw] OR Kypros[tw] OR Kibris[tw] OR kypriaki*[tw] OR Nicosia[tw] OR Lefkosa[tw] OR Lefkosia[tw] OR Famagusta[tw] OR Magusa[tw] OR Ammochostos[tw] OR Gazimagusa[tw] OR Kyrenia[tw] OR Girne[tw] OR Keryneia[tw] OR Larnaca[tw] OR Larnaka[tw] OR Limassol[tw] OR Lemesos[tw] OR Limasol[tw] OR Leymosun[tw] OR Paphos[tw] OR Pafos[tw] OR Baf[tw] OR Gazibaf[tw] OR Protaras[tw] OR Pergamos[tw] OR Beyarmudu[tw] OR Morfou[tw] OR Guzelyurt[tw] OR Omorfo[tw] OR Morphou[tw] OR Aradippou[tw])) OR (Cyprus[ad] OR Cypriot*[ad] OR Kypros[ad] OR Kibris[ad] OR kypriaki*[ad] OR Nicosia[ad] OR Lefkosa[ad] OR Lefkosia[ad] OR Famagusta[ad] OR Magusa[ad] OR Ammochostos[ad] OR Gazimagusa[ad] OR Kyrenia[ad] OR Girne[ad] OR Keryneia[ad] OR Larnaca[ad] OR Larnaka[ad] OR Limassol[ad] OR Lemesos[ad] OR Limasol[ad] OR Leymosun[ad] OR Paphos[ad] OR Pafos[ad] OR Baf[ad] OR Gazibaf[ad] OR Protaras[ad] OR Pergamos[ad] OR Beyarmudu[ad] OR Morfou[ad] OR Guzelyurt[ad] OR Omorfo[ad] OR Morphou[ad] OR Aradippou[ad])) OR (((Croatia*[tw] OR Hrvatsk*[tw] OR hrvat[tw] OR Bjelovar[tw] OR "Bjelovarsko bilogorska"[tw] OR "Brod Posavina"[tw] OR "Brodsko posavska"[tw] OR "Dubrovnik Neretva"[tw] OR "dubrovacko neretvanska"[tw] OR Istria[tw] OR Istarska[tw] OR Karlovacka[tw] OR Karlovac[tw] OR "Koprivnicko krizevacka"[tw] OR Koprivnica[tw] OR Krizevci[tw] OR "Krapina Zagorje"[tw] OR "Krapinsko zagorska"[tw] OR "Lika Senj"[tw] OR "Licko senjska"[tw] OR Medimurska[tw] OR Medimurje[tw] OR Osijek[tw] OR Osjecko[tw] OR Baranja[tw] OR "Osjecko baranjska"[tw] OR "Pozega Slavonia"[tw] OR "Pozesko slavonska"[tw] OR "Primorje Gorski Kotar"[tw] OR "Primorsko goranska"[tw] OR "Sibensko kninska"[tw] OR "Sibensko kninske"[tw] OR Sibenik[tw] OR Knin[tw] OR Sisak[tw] OR "Sisacko moslavacka"[tw] OR Moslavina[tw] OR "Splitsko dalmatinska"[tw] OR Split[tw] OR Dalmatia[tw] OR Varazdin[tw] OR Varazdinska[tw] OR "Viroviticko-podravska"[tw] OR Virovitica[tw] OR Podravina[tw] OR "Vukovarsko srijemska"[tw] OR Vukovar[tw] OR Srijem[tw] OR Zadar[tw] OR Zadarska[tw] OR Zagreb[tw] OR Zagrebacka[tw] OR Rijeka[tw] OR "Velika gorica"[tw] OR "Slavonski brod"[tw] OR Pula[tw])) OR (Croatia*[ad] OR Hrvatsk*[ad] OR hrvat[ad] OR Bjelovar[ad] OR "Bjelovarsko bilogorska"[ad] OR "Brod Posavina"[ad] OR "Brodsko posavska"[ad] OR "Dubrovnik Neretva"[ad] OR "dubrovacko neretvanska"[ad] OR Istria[ad] OR Istarska[ad] OR Karlovacka[ad] OR Karlovac[ad] OR "Koprivnicko krizevacka"[ad] OR Koprivnica[ad] OR Krizevci[ad] OR "Krapina Zagorje"[ad] OR "Krapinsko zagorska"[ad] OR "Lika Senj"[ad] OR "Licko senjska"[ad] OR Medimurska[ad] OR Medimurje[ad] OR Osijek[ad] OR Osjecko[ad] OR Baranja[ad] OR "Osjecko baranjska"[ad] OR "Pozega Slavonia"[ad] OR "Pozesko slavonska"[ad] OR "Primorje Gorski Kotar"[ad] OR "Primorsko goranska"[ad] OR "Sibensko kninska"[ad] OR "Sibensko kninske"[ad] OR Sibenik[ad] OR Knin[ad] OR Sisak[ad] OR "Sisacko moslavacka"[ad] OR Moslavina[ad] OR "Splitsko dalmatinska"[ad] OR Split[ad] OR Dalmatia[ad] OR Varazdin[ad] OR Varazdinska[ad] OR "Viroviticko-podravska"[ad] OR Virovitica[ad] OR Podravina[ad] OR "Vukovarsko srijemska"[ad] OR Vukovar[ad] OR Srijem[ad] OR Zadar[ad] OR Zadarska[ad] OR Zagreb[ad] OR Zagrebacka[ad] OR Rijeka[ad] OR "Velika gorica"[ad] OR "Slavonski brod"[ad] OR Pula[ad])) OR (((Bulgaria*[tw] OR Sofia[tw] OR Gabrovo[tw] OR Blagoevgrad[tw] OR "Pirin Macedonia"[tw] OR Burgas[tw] OR Dobrich[tw] OR Haskovo[tw] OR Kardzhali[tw] OR Kurdzhali[tw] OR Kyustendil[tw] OR Lovech[tw] OR Montana[tw] OR Pazardzhik[tw] OR Pernik[tw] OR Pleven[tw] OR Plovdiv[tw] OR Razgrad[tw] OR Rousse[tw] OR Ruse[tw] OR Shumen[tw] OR Sliven[tw] OR Silistra[tw] OR Smolyan[tw] OR "Stara Zagora"[tw] OR Targovishte[tw] OR Varna[tw] OR Tarnovo[tw] OR Vidin[tw] OR Vratsa[tw] OR Vratza[tw] OR Yambol[tw])) OR (Bulgaria*[ad] OR Sofia[ad] OR Gabrovo[ad] OR Blagoevgrad[ad] OR "Pirin Macedonia"[ad] OR Burgas[ad] OR Dobrich[ad] OR Haskovo[ad] OR Kardzhali[ad] OR Kurdzhali[ad] OR Kyustendil[ad] OR Lovech[ad] OR Montana[ad] OR Pazardzhik[ad] OR Pernik[ad] OR Pleven[ad] OR Plovdiv[ad] OR Razgrad[ad] OR Rousse[ad] OR Ruse[ad] OR Shumen[ad] OR Sliven[ad] OR Silistra[ad] OR Smolyan[ad] OR "Stara Zagora"[ad] OR Targovishte[ad] OR Varna[ad] OR Tarnovo[ad] OR Vidin[ad] OR Vratsa[ad] OR Vratza[ad] OR Yambol[ad])) OR (((Belgi*[tw] OR Belge[tw] OR Belgisch[tw] OR Brussel*[tw] OR Bruxelles[tw] OR Bruxelloise[tw] OR Flemish[tw] OR Flamand[tw] OR Flemisch[tw] OR Flanders[tw] OR Flandern[tw] OR Flandre[tw] OR Vlaanderen[tw] OR Vlaams[tw] OR Flamande[tw] OR Waals[tw] OR Walloon*[tw] OR Wallon*[tw] OR Antwerp*[tw] OR Anvers[tw] OR Ostflandern[tw] OR "Vlaams Brabant"[tw] OR Limbourg[tw] OR Limburg[tw] OR Hainault[tw] OR Hainaut[tw] OR Henegouwen[tw] OR Hennegau[tw] OR Liege[tw] OR Luik[tw] OR Luttich[tw] OR Namur[tw] OR Namen[tw] OR Westflandern[tw] OR "Waals Brabant"[tw] OR Ghent[tw] OR Gent[tw] OR Gand[tw] OR Charleroi[tw] OR Bruges[tw] OR Brugge[tw] OR Schaerbeek[tw] OR Schaarbeek[tw] OR Anderlecht[tw] OR Leuven[tw] OR Louvain[tw] OR Hasselt[tw] OR Mons[tw] OR Wavre[tw] OR Waver[tw])) OR (Belgi*[ad] OR Belge[ad] OR Belgisch[ad] OR Brussel*[ad] OR Bruxelles[ad] OR Bruxelloise[ad] OR Flemish[ad] OR Flamand[ad] OR Flemisch[ad] OR Flanders[ad] OR Flandern[ad] OR Flandre[ad] OR Vlaanderen[ad] OR Vlaams[ad] OR Flamande[ad] OR Waals[ad] OR Walloon*[ad] OR Wallon*[ad] OR Antwerp*[ad] OR Anvers[ad] OR Ostflandern[ad] OR "Vlaams Brabant"[ad] OR Limbourg[ad] OR Limburg[ad] OR Hainault[ad] OR Hainaut[ad] OR Henegouwen[ad] OR Hennegau[ad] OR Liege[ad] OR Luik[ad] OR Luttich[ad] OR Namur[ad] OR Namen[ad] OR Westflandern[ad] OR "Waals Brabant"[ad] OR Ghent[ad] OR Gent[ad] OR Gand[ad] OR Charleroi[ad] OR Bruges[ad] OR Brugge[ad] OR Schaerbeek[ad] OR Schaarbeek[ad] OR Anderlecht[ad] OR Leuven[ad] OR Louvain[ad] OR Hasselt[ad] OR Mons[ad] OR Wavre[ad] OR Waver[ad])) OR (((Austria*[tw] OR Vienna[tw] OR Wien[tw] OR Osterreich*[tw] OR Sudosterreich[tw] OR Westosterreich[tw] OR Niederosterreich[tw] OR Burgenland[tw] OR Carinthia[tw] OR Karinthia[tw] OR Karnten[tw] OR Oberosterreich[tw] OR Styria[tw] OR Steiermark[tw] OR Salzburg[tw] OR Saizburg[tw] OR Tyrol[tw] OR Tirol[tw] OR Becs[tw] OR Vorarlberg[tw] OR Bregenz[tw] OR Linz[tw] OR Eisenstadt[tw] OR Innsbruck[tw] OR Graz[tw] OR Klagenfurt[tw] OR Polten[tw] OR Villach[tw] OR Wels[tw] OR Dornbirn[tw] OR Feldkirch[tw] OR Steyr[tw])) OR (Austria*[ad] OR Vienna[ad] OR Wien[ad] OR Osterreich*[ad] OR Sudosterreich[ad] OR Westosterreich[ad] OR Niederosterreich[ad] OR Burgenland[ad] OR Carinthia[ad] OR Karnten[ad] OR Oberosterreich[ad] OR Styria[ad] OR Steiermark[ad] OR Salzburg[ad] OR Saizburg[ad] OR Tyrol[ad] OR Tirol[ad] OR Becs[ad] OR Vorarlberg[ad] OR Bregenz[ad] OR Linz[ad] OR Eisenstadt[ad] OR Innsbruck[ad] OR Graz[ad] OR Klagenfurt[ad] OR Polten[ad] OR Villach[ad] OR Wels[ad] OR Dornbirn[ad] OR Feldkirch[ad] OR Steyr[ad]))) OR (((((((((Iceland[tw] OR Icelandic*[tw] OR islenska*[tw] OR Icelander*[tw] OR islendinga*[tw] OR Reykjavik[tw] OR Reykjavikurborg[tw] OR Hofudborgarsvaedid[tw] OR Sudurnes[tw] OR Vesturland[tw] OR Vestfirdir[tw] OR Westfjords[tw] OR Nordurland[tw] OR Austurland[tw] OR Sudurland[tw] OR Kopavogur[tw] OR Hafnarfjordur[tw])) OR (Iceland[ad] OR Icelandic*[ad] OR islenska*[ad] OR Icelander*[ad] OR islendinga*[ad] OR Reykjavik[ad] OR Reykjavikurborg[ad] OR Hofudborgarsvaedid[ad] OR Sudurnes[ad] OR Vesturland[ad] OR Vestfirdir[ad] OR Westfjords[ad] OR Nordurland[ad] OR Austurland[ad] OR Sudurland[ad] OR Kopavogur[ad] OR Hafnarfjordur[ad])) OR (Switzerland[tw] OR Schweiz[tw] OR Schweizerische[tw] OR Swiss[tw] OR Suisse[tw] OR Aargau[tw] OR Argovia[tw] OR Ausserrhoden[tw] OR "Outer Rhodes"[tw] OR Innerrhoden[tw] OR "Inner Rhodes"[tw] OR Basel[tw] OR Bern[tw] OR Berne[tw] OR Fribourg[tw] OR Freiburg[tw] OR Geneva[tw] OR Geneve[tw] OR Glarus[tw] OR Graubunden[tw] OR Grisons[tw] OR Grigioni[tw] OR jura[tw] OR Lucerne[tw] OR Luzern[tw] OR Neuchatel[tw] OR Zurich[tw] OR (Uri[tw] AND (canton[tw] OR Kanton[tw])) OR Schwyz[tw] OR Obwalden[tw] OR Nidwalden[tw] OR Zug[tw] OR Solothurn[tw] OR Schaffhausen[tw] OR Thurgau[tw] OR Thurgovia[tw] OR Ticino[tw] OR Tessin[tw] OR Vaud[tw] OR Valais[tw] OR Wallis[tw] OR "St Gallen"[tw] OR Lausanne[tw] OR Winterthur[tw] OR Winterthour[tw] OR Lugano[tw] OR Biel[tw] OR Bienne[tw])) OR (Switzerland[ad] OR Schweiz[ad] OR Schweizerische[ad] OR Swiss[ad] OR Suisse[ad] OR Aargau[ad] OR Argovia[ad] OR Ausserrhoden[ad] OR "Outer Rhodes"[ad] OR Innerrhoden[ad] OR "Inner Rhodes"[ad] OR Basel[ad] OR Bern[ad] OR Berne[ad] OR Fribourg[ad] OR Freiburg[ad] OR Geneva[ad] OR Geneve[ad] OR Glarus[ad] OR Graubunden[ad] OR Grisons[ad] OR Grigioni[ad] OR jura[ad] OR Lucerne[ad] OR Luzern[ad] OR Neuchatel[ad] OR Zurich[ad] OR (Uri[ad] AND (canton[ad] OR Kanton[ad])) OR Schwyz[ad] OR Obwalden[ad] OR Nidwalden[ad] OR Zug[ad] OR Solothurn[ad] OR Schaffhausen[ad] OR Thurgau[ad] OR Thurgovia[ad] OR Ticino[ad] OR Tessin[ad] OR Vaud[ad] OR Valais[ad] OR Wallis[ad] OR "St Gallen"[ad] OR Lausanne[ad] OR Winterthur[ad] OR Winterthour[ad] OR Lugano[ad] OR Biel[ad] OR Bienne[ad])) OR (Norway[tw] OR Norwegian*[tw] OR Norge[tw] OR Noreg[tw] OR Norgga[tw] OR Ostfold[tw] OR Akershus[tw] OR Oslo[tw] OR Hedmark[tw] OR Oppland[tw] OR Buskerud[tw] OR Vestfold[tw] OR Telemark[tw] OR "Aust Agder"[tw] OR "Vest Agder"[tw] OR Rogaland[tw] OR Hordaland[tw] OR "Sogn og fjordane"[tw] OR "Sogn and fjordane"[tw] OR "sogn fjordane"[tw] OR "More og Romsdal"[tw] OR "More and Romsdal"[tw] OR "More Romsdal"[tw] OR Trondelag[tw] OR Nordland[tw] OR Troms[tw] OR Finnmark[tw] OR Bergen[tw] OR Stavanger[tw] OR Sandnes[tw] OR Trondheim[tw] OR Kristiansand[tw] OR Drammen[tw] OR Fredrikstad[tw] OR Sarpsborg[tw] OR Porsgrunn[tw] OR Skien[tw] OR Tonsberg[tw] OR Alesund[tw])) OR (Norway[ad] OR Norwegian*[ad] OR Norge[ad] OR Noreg[ad] OR Norgga[ad] OR Ostfold[ad] OR Akershus[ad] OR Oslo[ad] OR Hedmark[ad] OR Oppland[ad] OR Buskerud[ad] OR Vestfold[ad] OR Telemark[ad] OR "Aust Agder"[ad] OR "Vest Agder"[ad] OR Rogaland[ad] OR Hordaland[ad] OR "Sogn og fjordane"[ad] OR "Sogn and fjordane"[ad] OR "sogn fjordane"[ad] OR "More og Romsdal"[ad] OR "More and Romsdal"[ad] OR "More Romsdal"[ad] OR Trondelag[ad] OR Nordland[ad] OR Troms[ad] OR Finnmark[ad] OR Bergen[ad] OR Stavanger[ad] OR Sandnes[ad] OR Trondheim[ad] OR Kristiansand[ad] OR Drammen[ad] OR Fredrikstad[ad] OR Sarpsborg[ad] OR Porsgrunn[ad] OR Skien[ad] OR Tonsberg[ad] OR Alesund[ad])) OR (Liechtenstein[tw] OR Vaduz[tw] OR Triesenberg[tw] OR Triesen[tw] OR Schellenberg[tw] OR Schaan[tw] OR Ruggell[tw] OR Planken[tw] OR Mauren[tw] OR Gamprin[tw] OR Eschen[tw] OR Balzers[tw])) OR (Liechtenstein[ad] OR Vaduz[ad] OR Triesenberg[ad] OR Triesen[ad] OR Schellenberg[ad] OR Schaan[ad] OR Ruggell[ad] OR Planken[ad] OR Mauren[ad] OR Gamprin[ad] OR Eschen[ad] OR Balzers[ad])) OR ("European Union"[Mesh] OR "Europe"[MeSH] OR Europa[tw] OR Europe*[tw] OR Scandinavia*[tw] OR Scandinavia*[ad] OR Mediterranean[tw] OR "EEA countries"[tw] OR "EU country"[tw] OR "EU countries"[tw] OR Mediterranean[ad] OR Europe*[ad] OR Baltic[tw] OR Baltic[ad] OR Yugoslavia[tw] OR Jugoslavija[tw] OR Jugoslavija[AD] OR Yugoslavia[ad] OR "EU country"[tw] OR "Eu countries"[tw] OR global*[tw] OR world[tw] OR worldwide[tw])** |  |

## Embase search (Embase.com)

Date of the search: 16/03/2015

Language limit: no limits

Date limits: from 2005 to 2015

No of results: 7801

| No. | Query | Results |
| --- | --- | --- |
| #9 | **#1** AND **#7** AND [2005-2015]/py | **7801** |
| #8 | **#1** AND **#7** | **12837** |
| #7 | **#4** OR **#5** OR **#6** | **24163** |
| #6 | (**epidemiolog*** NEAR/5 (**'hepatitis b'** OR **'hepatitis c'** OR **hepaciviru*** OR **'hbv'** OR **'hcv'** OR **hbsag** OR **'hbs ag'** OR **'australia antigen'** OR **'australia antigens'**)):ti | **1146** |
| #5 | (**prevalence** NEAR/5 (**'hepatitis b'** OR **'hepatitis c'** OR **hepaciviru*** OR **'hbv'** OR **'hcv'** OR **hbsag** OR **'hbs ag'** OR **'australia antigen'** OR **'australia antigens'**)):ab,ti | **10785** |
| #4 | **#2** AND **#3** | **20368** |
| #3 | **'hepatitis b'**/exp OR **'hepatitis c'**/exp OR **'hepatitis b virus'**/exp OR **'hepatitis c virus'**/exp OR **'hepatitis b'**:ab,ti OR **'hepatitis c'**:ab,ti OR **hepaciviru***:ab,ti OR **'hbv'**:ab,ti OR **'hcv'**:ab,ti OR **hbsag**:ab,ti OR **'hbs ag'**:ab,ti OR **'hepatitis b antibody'**/exp OR **'hepatitis b surface antigen'**/exp OR **'australia antigen'**:ab,ti OR **'australia antigens'**:ab,ti OR **'hepatitis c antigen'**/exp OR **'hepatitis c antibody'**/exp | **201216** |
| #2 | **'prevalence'**/exp OR **'seroepidemiology'**/exp OR **'disease surveillance'**/exp OR **'sero epidemiology'**:ab,ti OR **'sero epidemiological'**:ab,ti OR **'sero epidemiologic'**:ab,ti OR **seroepidemiolog***:ab,ti OR **surveillance**:ti OR **serolog***:ti OR **serosurvey***:ab,ti OR **seroprevalence***:ab,ti OR **'population surveillance'**:ab,ti | **513612** |
| #1 |  | **11070462** |
|  | \| #1.8 \| **#1.1** OR **#1.3** OR **#1.4** OR **#1.5** OR **#1.6** OR **#1.7** \| **11070462** \| \| --- \| --- \| --- \| \| #1.7 \| **'united kingdom'**:ad OR **britain**:ad OR **british**:ad OR (**england**:ad NOT **'new england'**:ad) OR **english**:ad OR **scotland**:ad OR **scottish**:ad OR **wales**:ad OR **welsh**:ad OR **'northen ireland'**:ad OR **london**:ad OR **'east midlands'**:ad OR **'west midlands'**:ad OR **yorkshire**:ad OR **'east anglia'**:ad OR **bedfordshire**:ad OR **hertfordshire**:ad OR **essex**:ad OR **peterborough**:ad OR **cambridgeshire**:ad OR **norfolk**:ad OR **suffolk**:ad OR **luton**:ad OR **bedford**:ad OR **'southend on sea'**:ad OR **thurrock**:ad OR **derbyshire**:ad OR **nottinghamshire**:ad OR **leicestershire**:ad OR **rutland**:ad OR **lincolnshire**:ad OR **derby**:ad OR **leicester**:ad OR **northamptonshire**:ad OR **nottingham**:ad OR **'tyne and wear'**:ad OR **'tees valley'**:ad OR **'durham'**:ad OR **darlington**:ad OR **hartlepool**:ad OR **'stockton on tees'**:ad OR **northumberland**:ad OR **teesside**:ad OR **sunderland**:ad OR **cumbria**:ad OR **cheshire**:ad OR **manchester**:ad OR **lancashire**:ad OR **merseyside**:ad OR (**blackburn**:ad AND **darwen**:ad) OR **blackpool**:ad OR **chester**:ad OR **liverpool**:ad OR **sefton**:ad OR **warrington**:ad OR **wirral**:ad OR **berkshire**:ad OR **buckinghamshire**:ad OR **oxfordshire**:ad OR **hampshire**:ad OR **'isle of wight'**:ad OR **kent**:ad OR **surrey**:ad OR **sussex**:ad OR (**brighton**:ad AND **hove**:ad) OR **'milton keynes'**:ad OR **portsmouth**:ad OR **southampton**:ad OR **devon**:ad OR **dorset**:ad OR **somerset**:ad OR **gloucestershire**:ad OR **wiltshire**:ad OR **bath**:ad OR **bournemouth**:ad OR **poole**:ad OR **bristol**:ad OR **plymouth**:ad OR **swindon**:ad OR **torbay**:ad OR **herefordshire**:ad OR **staffordshire**:ad OR **birmingham**:ad OR **coventry**:ad OR **dudley**:ad OR **sandwell**:ad OR **shropshire**:ad OR **solihull**:ad OR **'stoke on trent'**:ad OR **telford**:ad OR **wrekin**:ad OR **walsall**:ad OR **warwickshire**:ad OR **wolverhampton**:ad OR **worcestershire**:ad OR **barnsley**:ad OR **doncaster**:ad OR **rotherham**:ad OR **bradford**:ad OR **calderdale**:ad OR **kirklees**:ad OR **kingston**:ad OR **leeds**:ad OR **sheffield**:ad OR **wakefield**:ad OR (**york**:ad NOT **'new york'**:ad) OR **antrim**:ad OR **ards**:ad OR **armagh**:ad OR **ballymena**:ad OR **ballymoney**:ad OR **banbridge**:ad OR **carrickfergus**:ad OR **castlereagh**:ad OR **coleraine**:ad OR **cookstown**:ad OR **craigavon**:ad OR (**down**:ad AND (**district**:ad OR **council**:ad)) OR **dungannon**:ad OR **fermanagh**:ad OR **larne**:ad OR **limavady**:ad OR **lisburn**:ad OR **magherafelt**:ad OR **moyle**:ad OR (**newry**:ad AND **mourne**:ad) OR **newtownabbey**:ad OR **omagh**:ad OR **strabane**:ad OR **londonderry**:ad OR **tyrone**:ad OR **belfast**:ad OR **aberdeen**:ad OR **aberdeenshire**:ad OR **angus**:ad OR **dundee**:ad OR (**argyll**:ad AND **bute**:ad) OR **clackmannanshire**:ad OR **fife**:ad OR **ayrshire**:ad OR **dunbartonshire**:ad OR **lothian**:ad OR **renfrewshire**:ad OR **edinburgh**:ad OR **falkirk**:ad OR **glasgow**:ad OR **highland***:ad OR **inverclyde**:ad OR **midlothian**:ad OR **moray**:ad OR **lanarkshire**:ad OR (**perth**:ad AND **kinross**:ad) OR **stirling**:ad OR **'orkney islands'**:ad OR **'eileanan siar'**:ad OR **'shetland islands'**:ad OR **bridgend**:ad OR **'neath port talbot'**:ad OR **cardiff**:ad OR (**vale**:ad AND **glamorgan**:ad) OR **'central valleys'**:ad OR **conwy**:ad OR **denbighshire**:ad OR **flintshire**:ad OR **wrexham**:ad OR **'gwent valleys'**:ad OR **gwynedd**:ad OR (**isle**:ad AND **anglesey**:ad) OR **'monmouthshire'**:ad OR **'newport'**:ad OR **powys**:ad OR **swansea**:ad OR **ceredigion**:ad OR **carmarthenshire**:ad OR **pembrokeshire**:ad OR **'merthyr tydfil'**:ad OR **'rhondda cynon taff'**:ad OR **'blaenau gwent'**:ad OR **caerphilly**:ad OR **torfaen**:ad OR **caithness**:ad OR **'sutherland and ross'**:ad OR **cromarty**:ad OR **teeside**:ad OR **tyneside**:ad OR **wearside**:ad OR **'west mercia'**:ad OR **avon**:ad OR **ulster**:ad OR **derry**:ad OR **medway**:ad OR **'east riding'**:ad OR **'west riding'**:ad OR **'lake district'**:ad OR **'peak district'**:ad OR **cumberland**:ad OR **dartmoor**:ad OR **exmoor**:ad OR **'united kingdom'**:ab,ti OR **britain**:ab,ti OR **british**:ab,ti OR (**england**:ab,ti NOT **'new england'**:ab,ti) OR **english**:ab,ti OR **scotland**:ab,ti OR **scottish**:ab,ti OR **wales**:ab,ti OR **welsh**:ab,ti OR **'northen ireland'**:ab,ti OR **london**:ab,ti OR **'east midlands'**:ab,ti OR **'west midlands'**:ab,ti OR **yorkshire**:ab,ti OR **'east anglia'**:ab,ti OR **bedfordshire**:ab,ti OR **hertfordshire**:ab,ti OR **essex**:ab,ti OR **peterborough**:ab,ti OR **cambridgeshire**:ab,ti OR **norfolk**:ab,ti OR **suffolk**:ab,ti OR **luton**:ab,ti OR **bedford**:ab,ti OR **'southend on sea'**:ab,ti OR **thurrock**:ab,ti OR **derbyshire**:ab,ti OR **nottinghamshire**:ab,ti OR **leicestershire**:ab,ti OR **rutland**:ab,ti OR **lincolnshire**:ab,ti OR **derby**:ab,ti OR **leicester**:ab,ti OR **northamptonshire**:ab,ti OR **nottingham**:ab,ti OR **'tyne and wear'**:ab,ti OR **'tees valley'**:ab,ti OR **'durham'**:ab,ti OR **darlington**:ab,ti OR **hartlepool**:ab,ti OR **'stockton on tees'**:ab,ti OR **northumberland**:ab,ti OR **teesside**:ab,ti OR **sunderland**:ab,ti OR **cumbria**:ab,ti OR **cheshire**:ab,ti OR **manchester**:ab,ti OR **lancashire**:ab,ti OR **merseyside**:ab,ti OR (**blackburn**:ab,ti AND **darwen**:ab,ti) OR **blackpool**:ab,ti OR **chester**:ab,ti OR **liverpool**:ab,ti OR **sefton**:ab,ti OR **warrington**:ab,ti OR **wirral**:ab,ti OR **berkshire**:ab,ti OR **buckinghamshire**:ab,ti OR **oxfordshire**:ab,ti OR **hampshire**:ab,ti OR **'isle of wight'**:ab,ti OR **kent**:ab,ti OR **surrey**:ab,ti OR **sussex**:ab,ti OR (**brighton**:ab,ti AND **hove**:ab,ti) OR **'milton keynes'**:ab,ti OR **portsmouth**:ab,ti OR **southampton**:ab,ti OR **devon**:ab,ti OR **dorset**:ab,ti OR **somerset**:ab,ti OR **gloucestershire**:ab,ti OR **wiltshire**:ab,ti OR **bath**:ab,ti OR **bournemouth**:ab,ti OR **poole**:ab,ti OR **bristol**:ab,ti OR **plymouth**:ab,ti OR **swindon**:ab,ti OR **torbay**:ab,ti OR **herefordshire**:ab,ti OR **staffordshire**:ab,ti OR **birmingham**:ab,ti OR **coventry**:ab,ti OR **dudley**:ab,ti OR **sandwell**:ab,ti OR **shropshire**:ab,ti OR **solihull**:ab,ti OR **'stoke on trent'**:ab,ti OR **telford**:ab,ti OR **wrekin**:ab,ti OR **walsall**:ab,ti OR **warwickshire**:ab,ti OR **wolverhampton**:ab,ti OR **worcestershire**:ab,ti OR **barnsley**:ab,ti OR **doncaster**:ab,ti OR **rotherham**:ab,ti OR **bradford**:ab,ti OR **calderdale**:ab,ti OR **kirklees**:ab,ti OR **kingston**:ab,ti OR **leeds**:ab,ti OR **sheffield**:ab,ti OR **wakefield**:ab,ti OR (**york**:ab,ti NOT **'new york'**:ab,ti) OR **antrim**:ab,ti OR **ards**:ab,ti OR **armagh**:ab,ti OR **ballymena**:ab,ti OR **ballymoney**:ab,ti OR **banbridge**:ab,ti OR **carrickfergus**:ab,ti OR **castlereagh**:ab,ti OR **coleraine**:ab,ti OR **cookstown**:ab,ti OR **craigavon**:ab,ti OR (**down**:ab,ti AND (**district**:ab,ti OR **council**:ab,ti)) OR **dungannon**:ab,ti OR **fermanagh**:ab,ti OR **larne**:ab,ti OR **limavady**:ab,ti OR **lisburn**:ab,ti OR **magherafelt**:ab,ti OR **moyle**:ab,ti OR (**newry**:ab,ti AND **mourne**:ab,ti) OR **newtownabbey**:ab,ti OR **omagh**:ab,ti OR **strabane**:ab,ti OR **londonderry**:ab,ti OR **tyrone**:ab,ti OR **belfast**:ab,ti OR **aberdeen**:ab,ti OR **aberdeenshire**:ab,ti OR **angus**:ab,ti OR **dundee**:ab,ti OR (**argyll**:ab,ti AND **bute**:ab,ti) OR **clackmannanshire**:ab,ti OR **fife**:ab,ti OR **ayrshire**:ab,ti OR **dunbartonshire**:ab,ti OR **lothian**:ab,ti OR **renfrewshire**:ab,ti OR **edinburgh**:ab,ti OR **falkirk**:ab,ti OR **glasgow**:ab,ti OR **highland***:ab,ti OR **inverclyde**:ab,ti OR **midlothian**:ab,ti OR **moray**:ab,ti OR **lanarkshire**:ab,ti OR (**perth**:ab,ti AND **kinross**:ab,ti) OR **stirling**:ab,ti OR **'orkney islands'**:ab,ti OR **'eileanan siar'**:ab,ti OR **'shetland islands'**:ab,ti OR **bridgend**:ab,ti OR **'neath port talbot'**:ab,ti OR **cardiff**:ab,ti OR (**vale**:ab,ti AND **glamorgan**:ab,ti) OR **'central valleys'**:ab,ti OR **conwy**:ab,ti OR **denbighshire**:ab,ti OR **flintshire**:ab,ti OR **wrexham**:ab,ti OR **'gwent valleys'**:ab,ti OR **gwynedd**:ab,ti OR (**isle**:ab,ti AND **anglesey**:ab,ti) OR **'monmouthshire'**:ab,ti OR **'newport'**:ab,ti OR **powys**:ab,ti OR **swansea**:ab,ti OR **ceredigion**:ab,ti OR **carmarthenshire**:ab,ti OR **pembrokeshire**:ab,ti OR **'merthyr tydfil'**:ab,ti OR **'rhondda cynon taff'**:ab,ti OR **'blaenau gwent'**:ab,ti OR **caerphilly**:ab,ti OR **torfaen**:ab,ti OR **caithness**:ab,ti OR **'sutherland and ross'**:ab,ti OR **cromarty**:ab,ti OR **teeside**:ab,ti OR **tyneside**:ab,ti OR **wearside**:ab,ti OR **'west mercia'**:ab,ti OR **avon**:ab,ti OR **ulster**:ab,ti OR **derry**:ab,ti OR **medway**:ab,ti OR **'east riding'**:ab,ti OR **'west riding'**:ab,ti OR **'lake district'**:ab,ti OR **'peak district'**:ab,ti OR **cumberland**:ab,ti OR **dartmoor**:ab,ti OR **exmoor**:ab,ti OR **sweden**:ad OR **sverige**:ad OR **swedish**:ad OR **svenska**:ad OR **stockholm***:ad OR **norrland**:ad OR **svealand**:ad OR **mellansverige**:ad OR **smaland**:ad OR **sydsverige**:ad OR **vastsverige**:ad OR **orebro**:ad OR **ostergotland***:ad OR **vastergotland***:ad OR **skara***:ad OR **bohus***:ad OR **dalsland**:ad OR **narke**:ad OR **sodermanland**:ad OR **uppsala**:ad OR **uppland**:ad OR **vastmanland***:ad OR **jamtland***:ad OR **harjedalen**:ad OR **vasternorrland***:ad OR **dalarna**:ad OR **kopparberg**:ad OR **gavleborg***:ad OR **gastrikland**:ad OR **halsingland**:ad OR **varmland***:ad OR **gotland***:ad OR **oland**:ad OR **jonkoping***:ad OR **kalmar***:ad OR **kronoberg***:ad OR **blekinge**:ad OR **skane***:ad OR **norrbotten***:ad OR **vasterbotten***:ad OR **lappland**:ad OR **angermanland**:ad OR **medelpad**:ad OR **halland***:ad OR **gotaland***:ad OR **gothenburg**:ad OR **goteborg***:ad OR **malmo***:ad OR **vasteras**:ad OR **linkoping**:ad OR **helsingborg**:ad OR **halsingborg**:ad OR **norrkoping**:ad OR **gavle**:ad OR **umea**:ad OR **lulea**:ad OR **karlstad**:ad OR **kalmar**:ad OR **huddinge**:ad OR **solna**:ad OR **ostersjo***:ad OR **malaren***:ad OR **malardalen**:ad OR **sweden**:ab,ti OR **sverige**:ab,ti OR **swedish**:ab,ti OR **svenska**:ab,ti OR **stockholm***:ab,ti OR **norrland**:ab,ti OR **svealand**:ab,ti OR **mellansverige**:ab,ti OR **smaland**:ab,ti OR **sydsverige**:ab,ti OR **vastsverige**:ab,ti OR **orebro**:ab,ti OR **ostergotland***:ab,ti OR **vastergotland***:ab,ti OR **skara***:ab,ti OR **bohus***:ab,ti OR **dalsland**:ab,ti OR **narke**:ab,ti OR **sodermanland**:ab,ti OR **uppsala**:ab,ti OR **uppland**:ab,ti OR **vastmanland***:ab,ti OR **jamtland***:ab,ti OR **harjedalen**:ab,ti OR **vasternorrland***:ab,ti OR **dalarna**:ab,ti OR **kopparberg**:ab,ti OR **gavleborg***:ab,ti OR **gastrikland**:ab,ti OR **halsingland**:ab,ti OR **varmland***:ab,ti OR **gotland***:ab,ti OR **oland**:ab,ti OR **jonkoping***:ab,ti OR **kalmar***:ab,ti OR **kronoberg***:ab,ti OR **blekinge**:ab,ti OR **skane***:ab,ti OR **norrbotten***:ab,ti OR **vasterbotten***:ab,ti OR **lappland**:ab,ti OR **angermanland**:ab,ti OR **medelpad**:ab,ti OR **halland***:ab,ti OR **gotaland***:ab,ti OR **gothenburg**:ab,ti OR **goteborg***:ab,ti OR **malmo***:ab,ti OR **vasteras**:ab,ti OR **linkoping**:ab,ti OR **helsingborg**:ab,ti OR **halsingborg**:ab,ti OR **norrkoping**:ab,ti OR **gavle**:ab,ti OR **umea**:ab,ti OR **lulea**:ab,ti OR **karlstad**:ab,ti OR **kalmar**:ab,ti OR **huddinge**:ab,ti OR **solna**:ab,ti OR **ostersjo***:ab,ti OR **malaren***:ab,ti OR **malardalen**:ab,ti OR **spain**:ad OR **espana**:ad OR **spanish**:ad OR **espanol***:ad OR **spaniard***:ad OR **madrid**:ad OR **andalucia**:ad OR **andalusia**:ad OR **aragon**:ad OR **cantabria**:ad OR **canarias**:ad OR **'canary islands'**:ad OR **'castile and leon'**:ad OR **'castilla y leon'**:ad OR **'castile la mancha'**:ad OR **'castilla la mancha'**:ad OR **cataluna**:ad OR **catalonia**:ad OR **ceuta**:ad OR **melilla**:ad OR **navarra**:ad OR **navarre**:ad OR **valencian**:ad OR **extremadura**:ad OR **galicia**:ad OR **balears**:ad OR **'balearic islands'**:ad OR **baleares**:ad OR **'la rioja'**:ad OR **'pais vasco'**:ad OR **'basque country'**:ad OR **coruna**:ad OR **alava**:ad OR **araba**:ad OR **albacete**:ad OR **alicante**:ad OR **alacant**:ad OR **almeria**:ad OR **asturias**:ad OR **avila**:ad OR **badajoz**:ad OR **badajos**:ad OR **barcelona**:ad OR **burgos**:ad OR **caceres**:ad OR **cadiz**:ad OR **castellon**:ad OR **castello**:ad OR **'ciudad real'**:ad OR **cordoba**:ad OR **cuenca**:ad OR **eivissa**:ad OR **ibiza**:ad OR **formentera**:ad OR **'el hierro'**:ad OR **fuerteventura**:ad OR **girona**:ad OR **gerona**:ad OR **'gran canaria'**:ad OR **granada**:ad OR **guadalajara**:ad OR **guipuzcoa**:ad OR **gipuzkoa**:ad OR **huelva**:ad OR **huesca**:ad OR **jaen**:ad OR **'la gomera'**:ad OR **'la palma'**:ad OR **lanzarote**:ad OR **leon**:ad OR **lleida**:ad OR **lerida**:ad OR **lugo**:ad OR **malaga**:ad OR **mallorca**:ad OR **majorca**:ad OR **menorca**:ad OR **minorca**:ad OR **murcia**:ad OR **ourense**:ad OR **orense**:ad OR **palencia**:ad OR **pontevedra**:ad OR **salamanca**:ad OR **segovia**:ad OR **sevilla**:ad OR **seville**:ad OR **soria**:ad OR **tarragona**:ad OR **tenerife**:ad OR **teruel**:ad OR **toledo**:ad OR **valencia**:ad OR **valladolid**:ad OR **vizcaya**:ad OR **biscay**:ad OR **zamora**:ad OR **zaragoza**:ad OR **saragossa**:ad OR **bilbao**:ad OR **bilbo**:ad OR **compostela**:ad OR **'san sebastian'**:ad OR **donostia**:ad OR **vitoria**:ad OR **oviedo**:ad OR **pamplona**:ad OR **logrono**:ad OR **gasteiz**:ad OR **spain**:ab,ti OR **espana**:ab,ti OR **spanish**:ab,ti OR **espanol***:ab,ti OR **spaniard***:ab,ti OR **madrid**:ab,ti OR **andalucia**:ab,ti OR **andalusia**:ab,ti OR **aragon**:ab,ti OR **cantabria**:ab,ti OR **canarias**:ab,ti OR **'canary islands'**:ab,ti OR **'castile and leon'**:ab,ti OR **'castilla y leon'**:ab,ti OR **'castile la mancha'**:ab,ti OR **'castilla la mancha'**:ab,ti OR **cataluna**:ab,ti OR **catalonia**:ab,ti OR **ceuta**:ab,ti OR **melilla**:ab,ti OR **navarra**:ab,ti OR **navarre**:ab,ti OR **valencian**:ab,ti OR **extremadura**:ab,ti OR **galicia**:ab,ti OR **balears**:ab,ti OR **'balearic islands'**:ab,ti OR **baleares**:ab,ti OR **'la rioja'**:ab,ti OR **'pais vasco'**:ab,ti OR **'basque country'**:ab,ti OR **coruna**:ab,ti OR **alava**:ab,ti OR **araba**:ab,ti OR **albacete**:ab,ti OR **alicante**:ab,ti OR **alacant**:ab,ti OR **almeria**:ab,ti OR **asturias**:ab,ti OR **avila**:ab,ti OR **badajoz**:ab,ti OR **badajos**:ab,ti OR **barcelona**:ab,ti OR **burgos**:ab,ti OR **caceres**:ab,ti OR **cadiz**:ab,ti OR **castellon**:ab,ti OR **castello**:ab,ti OR **'ciudad real'**:ab,ti OR (**cordoba**:ab,ti NOT **argent***:ab,ti) OR **cuenca**:ab,ti OR **eivissa**:ab,ti OR **ibiza**:ab,ti OR **formentera**:ab,ti OR **'el hierro'**:ab,ti OR **fuerteventura**:ab,ti OR **girona**:ab,ti OR **gerona**:ab,ti OR **'gran canaria'**:ab,ti OR **granada**:ab,ti OR (**guadalajara**:ab,ti NOT **mexic***:ab,ti) OR **guipuzcoa**:ab,ti OR **gipuzkoa**:ab,ti OR **huelva**:ab,ti OR **huesca**:ab,ti OR **jaen**:ab,ti OR **'la gomera'**:ab,ti OR **'la palma'**:ab,ti OR **lanzarote**:ab,ti OR **leon**:ab,ti OR **lleida**:ab,ti OR **lerida**:ab,ti OR **lugo**:ab,ti OR **malaga**:ab,ti OR **mallorca**:ab,ti OR **majorca**:ab,ti OR **menorca**:ab,ti OR **minorca**:ab,ti OR **murcia**:ab,ti OR **ourense**:ab,ti OR **orense**:ab,ti OR **palencia**:ab,ti OR **pontevedra**:ab,ti OR **salamanca**:ab,ti OR **segovia**:ab,ti OR **sevilla**:ab,ti OR **seville**:ab,ti OR **soria**:ab,ti OR **tarragona**:ab,ti OR **tenerife**:ab,ti OR **teruel**:ab,ti OR **toledo**:ab,ti OR **valencia**:ab,ti OR **valladolid**:ab,ti OR **vizcaya**:ab,ti OR **biscay**:ab,ti OR **zamora**:ab,ti OR **zaragoza**:ab,ti OR **saragossa**:ab,ti OR **bilbao**:ab,ti OR **bilbo**:ab,ti OR **compostela**:ab,ti OR **'san sebastian'**:ab,ti OR **donostia**:ab,ti OR **vitoria**:ab,ti OR **oviedo**:ab,ti OR **pamplona**:ab,ti OR **logrono**:ab,ti OR **gasteiz**:ab,ti OR **slovenia***:ad OR **slovenija**:ad OR **ljubljana**:ad OR **gorenjska**:ad OR **carniola**:ad OR **goriska**:ad OR **gorizia**:ad OR **koroska**:ad OR **carinthia**:ad OR **'notranjsko kraska'**:ad OR **'obalno kraska'**:ad OR **'coastal krast'**:ad OR **podravska**:ad OR **pomurska**:ad OR **savinjska**:ad OR **spodnjeposavska**:ad OR **zasavska**:ad OR **osrednjeslovenska**:ad OR **maribor**:ad OR **celje**:ad OR **kranj**:ad OR **velenje**:ad OR **koper**:ad OR **capodistria**:ad OR **'novo mesto'**:ad OR **ptuj**:ad OR **trbovlje**:ad OR **kamnik**:ad OR **murska**:ad OR **sobota**:ad OR **'nova gorica'**:ad OR **slovenia***:ab,ti OR **slovenija**:ab,ti OR **ljubljana**:ab,ti OR **gorenjska**:ab,ti OR **carniola**:ab,ti OR **goriska**:ab,ti OR **gorizia**:ab,ti OR **koroska**:ab,ti OR **carinthia**:ab,ti OR **'notranjsko kraska'**:ab,ti OR **'obalno kraska'**:ab,ti OR **'coastal krast'**:ab,ti OR **podravska**:ab,ti OR **pomurska**:ab,ti OR **savinjska**:ab,ti OR **spodnjeposavska**:ab,ti OR **zasavska**:ab,ti OR **osrednjeslovenska**:ab,ti OR **maribor**:ab,ti OR **celje**:ab,ti OR **kranj**:ab,ti OR **velenje**:ab,ti OR **koper**:ab,ti OR **capodistria**:ab,ti OR **'novo mesto'**:ab,ti OR **ptuj**:ab,ti OR **trbovlje**:ab,ti OR **kamnik**:ab,ti OR **murska**:ab,ti OR **sobota**:ab,ti OR **'nova gorica'**:ab,ti OR **slovakia**:ab,ti OR **slovensk***:ab,ti OR **slovak***:ab,ti OR **bratislav***:ab,ti OR **nitrian***:ab,ti OR **nitra**:ab,ti OR **trencian***:ab,ti OR **trencin**:ab,ti OR **banskobystri***:ab,ti OR **'banska bystrica'**:ab,ti OR **zilina**:ab,ti OR **zilin***:ab,ti OR **trnava**:ab,ti OR **trnav***:ab,ti OR **presov**:ab,ti OR **presov***:ab,ti OR **kosic***:ab,ti OR (**martin**:ab,ti AND (**city**:ab,ti OR **svaty**:ab,ti)) OR **poprad**:ab,ti OR **slovakia**:ad OR **slovensk***:ad OR **slovak***:ad OR **bratislav***:ad OR **nitrian***:ad OR **nitra**:ad OR **trencian***:ad OR **trencin**:ad OR **banskobystri***:ad OR **'banska bystrica'**:ad OR **zilina**:ad OR **zilin***:ad OR **trnava**:ad OR **trnav***:ad OR **presov***:ad OR **kosic***:ad OR (**martin**:ad AND (**city**:ad OR **svaty**:ad)) OR **poprad**:ad \|  \| \| #1.6 \| **italy**:ab,ti OR **italia***:ab,ti OR **rome**:ab,ti OR **roma**:ab,ti OR **abruzzo**:ab,ti OR **abruzzi**:ab,ti OR **basilicata**:ab,ti OR **lucania**:ab,ti OR **calabria**:ab,ti OR **campania**:ab,ti OR **'emilia romagna'**:ab,ti OR **'friuli venezia giulia'**:ab,ti OR **lazio**:ab,ti OR **latium**:ab,ti OR **liguria***:ab,ti OR **lombardy**:ab,ti OR **lombardia**:ab,ti OR **marche**:ab,ti OR **marches**:ab,ti OR **molisano**:ab,ti OR **molise**:ab,ti OR **piedmont***:ab,ti OR **piemonte**:ab,ti OR **sardinia**:ab,ti OR **sardegna**:ab,ti OR **sicily**:ab,ti OR **sicilia**:ab,ti OR **toscana**:ab,ti OR **tuscany**:ab,ti OR **trentino**:ab,ti OR **trento**:ab,ti OR **umbria**:ab,ti OR **veneto**:ab,ti OR **triveneto**:ab,ti OR **puglia**:ab,ti OR **apulia**:ab,ti OR **bolzano**:ab,ti OR **bozen**:ab,ti OR **milan**:ab,ti OR **milano**:ab,ti OR **naples**:ab,ti OR **napoli**:ab,ti OR **turin**:ab,ti OR **torino**:ab,ti OR **palermo**:ab,ti OR **genoa**:ab,ti OR **genova**:ab,ti OR **florence**:ab,ti OR **firenze**:ab,ti OR **bari**:ab,ti OR **catania**:ab,ti OR **venezia**:ab,ti OR **venice**:ab,ti OR **padova**:ab,ti OR **padua**:ab,ti OR **siena**:ab,ti OR **bologna**:ab,ti OR **trieste**:ab,ti OR **urbino**:ab,ti OR **aosta**:ab,ti OR **aoste**:ab,ti OR **perugia**:ab,ti OR **brescia**:ab,ti OR **cagliari**:ab,ti OR **catanzaro**:ab,ti OR **'l aquila'**:ab,ti OR **ancona**:ab,ti OR **italy**:ad OR **italia***:ad OR **rome**:ad OR **roma**:ad OR **abruzzo**:ad OR **abruzzi**:ad OR **basilicata**:ad OR **lucania**:ad OR **calabria**:ad OR **campania**:ad OR **'emilia romagna'**:ad OR **'friuli venezia giulia'**:ad OR **lazio**:ad OR **latium**:ad OR **liguria***:ad OR **lombardy**:ad OR **lombardia**:ad OR **marche**:ad OR **marches**:ad OR **molisano**:ad OR **molise**:ad OR **piedmont***:ad OR **piemonte**:ad OR **sardinia**:ad OR **sardegna**:ad OR **sicily**:ad OR **sicilia**:ad OR **toscana**:ad OR **tuscany**:ad OR **trentino**:ad OR **trento**:ad OR **umbria**:ad OR **veneto**:ad OR **triveneto**:ad OR **puglia**:ad OR **apulia**:ad OR **bolzano**:ad OR **bozen**:ad OR **milan**:ad OR **milano**:ad OR **naples**:ad OR **napoli**:ad OR **turin**:ad OR **torino**:ad OR **palermo**:ad OR **genoa**:ad OR **genova**:ad OR **florence**:ad OR **firenze**:ad OR **bari**:ad OR **catania**:ad OR **venezia**:ad OR **venice**:ad OR **padova**:ad OR **padua**:ad OR **siena**:ad OR **bologna**:ad OR **trieste**:ad OR **urbino**:ad OR **aosta**:ad OR **aoste**:ad OR **perugia**:ad OR **brescia**:ad OR **cagliari**:ad OR **catanzaro**:ad OR **'l aquila'**:ad OR **ancona**:ad OR **ireland**:ab,ti OR **eire**:ab,ti OR **irish***:ab,ti OR **dublin**:ab,ti OR **fingal**:ab,ti OR **'dun laoghaire'**:ab,ti OR **wicklow**:ab,ti OR **wexford**:ab,ti OR **carlow**:ab,ti OR **kildare**:ab,ti OR **meath**:ab,ti OR **louth**:ab,ti OR **monaghan**:ab,ti OR **cavan**:ab,ti OR **longford**:ab,ti OR **westmeath**:ab,ti OR **offaly**:ab,ti OR **laois**:ab,ti OR **kilkenny**:ab,ti OR **waterford**:ab,ti OR **cork**:ab,ti OR **kerry**:ab,ti OR **limerick**:ab,ti OR **tipperary**:ab,ti OR **clare**:ab,ti OR **galway**:ab,ti OR **mayo**:ab,ti OR **roscommon**:ab,ti OR **sligo**:ab,ti OR **leitrim**:ab,ti OR **donegal**:ab,ti OR **drogheda**:ab,ti OR **dundalk**:ab,ti OR **swords**:ab,ti OR **bray**:ab,ti OR **navan**:ab,ti OR **leinster**:ab,ti OR **connacht**:ab,ti OR **ireland**:ad OR **eire**:ad OR **irish***:ad OR **dublin**:ad OR **fingal**:ad OR **'dun laoghaire'**:ad OR **wicklow**:ad OR **wexford**:ad OR **carlow**:ad OR **kildare**:ad OR **meath**:ad OR **louth**:ad OR **monaghan**:ad OR **cavan**:ad OR **longford**:ad OR **westmeath**:ad OR **offaly**:ad OR **laois**:ad OR **kilkenny**:ad OR **waterford**:ad OR **cork**:ad OR **kerry**:ad OR **limerick**:ad OR **tipperary**:ad OR **clare**:ad OR **galway**:ad OR **mayo**:ad OR **roscommon**:ad OR **sligo**:ad OR **leitrim**:ad OR **donegal**:ad OR **drogheda**:ad OR **dundalk**:ad OR **swords**:ad OR **bray**:ad OR **navan**:ad OR **leinster**:ad OR **connacht**:ad OR **hungar***:ab,ti OR **budapest**:ab,ti OR **transdanubia**:ab,ti OR **magyarorszag**:ab,ti OR **magyar**:ab,ti OR **dunantuli**:ab,ti OR **dunantul**:ab,ti OR **'great plain'**:ab,ti OR **'alfold es eszak'**:ab,ti OR **'eszak alfold'**:ab,ti OR **'del alfold'**:ab,ti OR **bacs**:ab,ti OR **kiskun**:ab,ti OR **'northen alfold'**:ab,ti OR **'sourthen alfold'**:ab,ti OR **baranya**:ab,ti OR **bekes**:ab,ti OR **borsod**:ab,ti OR **abauj**:ab,ti OR **zemplen**:ab,ti OR **fovaros**:ab,ti OR **csongrad**:ab,ti OR **fejer**:ab,ti OR **moson**:ab,ti OR **sopron**:ab,ti OR **hajdu**:ab,ti OR **bihar**:ab,ti OR **heves**:ab,ti OR **'jasz nagykun szolnok'**:ab,ti OR **komarom**:ab,ti OR **esztergom**:ab,ti OR **nograd**:ab,ti OR **pest**:ab,ti OR **somogy**:ab,ti OR **szabolcs**:ab,ti OR **szatmar**:ab,ti OR **bereg**:ab,ti OR **tolna**:ab,ti OR **vas**:ab,ti OR **veszprem**:ab,ti OR **zala**:ab,ti OR **zalaegerszeg**:ab,ti OR **debrecen**:ab,ti OR **miskolc**:ab,ti OR **szeged**:ab,ti OR **pecs**:ab,ti OR **gyor**:ab,ti OR **nyiregyhaza**:ab,ti OR **kecskemet**:ab,ti OR **szekesfehervar**:ab,ti OR **szombathely**:ab,ti OR **bekescsaba**:ab,ti OR **eger**:ab,ti OR **tatabanya**:ab,ti OR **salgotarjan**:ab,ti OR **kaposvar**:ab,ti OR **szekszard**:ab,ti OR **hungar***:ad OR **budapest**:ad OR **transdanubia**:ad OR **magyarorszag**:ad OR **magyar**:ad OR **dunantuli**:ad OR **dunantul**:ad OR **'great plain'**:ad OR **'alfold es eszak'**:ad OR **'eszak alfold'**:ad OR **'del alfold'**:ad OR **bacs**:ad OR **kiskun**:ad OR **'northen alfold'**:ad OR **'sourthen alfold'**:ad OR **baranya**:ad OR **bekes**:ad OR **borsod**:ad OR **abauj**:ad OR **zemplen**:ad OR **fovaros**:ad OR **csongrad**:ad OR **fejer**:ad OR **moson**:ad OR **sopron**:ad OR **hajdu**:ad OR **bihar**:ad OR **heves**:ad OR **'jasz nagykun szolnok'**:ad OR **komarom**:ad OR **esztergom**:ad OR **nograd**:ad OR **pest**:ad OR **somogy**:ad OR **szabolcs**:ad OR **szatmar**:ad OR **bereg**:ad OR **tolna**:ad OR **vas**:ad OR **veszprem**:ad OR **zala**:ad OR **zalaegerszeg**:ad OR **debrecen**:ad OR **miskolc**:ad OR **szeged**:ad OR **pecs**:ad OR **gyor**:ad OR **nyiregyhaza**:ad OR **kecskemet**:ad OR **szekesfehervar**:ad OR **szombathely**:ad OR **bekescsaba**:ad OR **eger**:ad OR **tatabanya**:ad OR **salgotarjan**:ad OR **kaposvar**:ad OR **szekszard**:ad OR **greece**:ad OR **'hellenic republic'**:ad OR **greek***:ad OR **ellada**:ad OR **'elliniki dimokratia'**:ad OR **hellas**:ad OR **hellenes**:ad OR **attica**:ad OR **attiki**:ad OR **makedonia**:ad OR **macedonia**:ad OR **thraki**:ad OR **thrace**:ad OR **crete**:ad OR **kriti**:ad OR **epirus**:ad OR **ipeiros**:ad OR **'ionia nisia'**:ad OR **'ionion neson'**:ad OR **'ionian islands'**:ad OR **'north aegean'**:ad OR **'aegean islands'**:ad OR **'nisoi agaiou'**:ad OR **'notio aigaio'**:ad OR **peloponnese**:ad OR **peloponnisos**:ad OR **'voreio aigaio'**:ad OR **'south aegean'**:ad OR **thessaly**:ad OR **thessalia**:ad OR **cycklades**:ad OR **kiklades**:ad OR **dodecanese**:ad OR **dodekanisa**:ad OR **'mount athos'**:ad OR **'omicronros alphathos'**:ad OR **athens**:ad OR **athina**:ad OR **thessaloniki**:ad OR **thessalonica**:ad OR **patras**:ad OR **patra**:ad OR **heraklion**:ad OR **heraclion**:ad OR **iraklion**:ad OR **irakleion**:ad OR **iraklio**:ad OR **larissa**:ad OR **larisa**:ad OR **volos**:ad OR **rhodes**:ad OR **rodos**:ad OR **ioannina**:ad OR **janina**:ad OR **yannena**:ad OR **chania**:ad OR **chalcis**:ad OR **chalkida**:ad OR **alexandroupoli**:ad OR **greece**:ab,ti OR **'hellenic republic'**:ab,ti OR **greek***:ab,ti OR **ellada**:ab,ti OR **'elliniki dimokratia'**:ab,ti OR **hellas**:ab,ti OR **hellenes**:ab,ti OR **attica**:ab,ti OR **attiki**:ab,ti OR **makedonia**:ab,ti OR **macedonia**:ab,ti OR **thraki**:ab,ti OR **thrace**:ab,ti OR **crete**:ab,ti OR **kriti**:ab,ti OR **epirus**:ab,ti OR **ipeiros**:ab,ti OR **'ionia nisia'**:ab,ti OR **'ionion neson'**:ab,ti OR **'ionian islands'**:ab,ti OR **'north aegean'**:ab,ti OR **'aegean islands'**:ab,ti OR **'nisoi agaiou'**:ab,ti OR **'notio aigaio'**:ab,ti OR **peloponnese**:ab,ti OR **peloponnisos**:ab,ti OR **'voreio aigaio'**:ab,ti OR **'south aegean'**:ab,ti OR **thessaly**:ab,ti OR **thessalia**:ab,ti OR **cycklades**:ab,ti OR **kiklades**:ab,ti OR **dodecanese**:ab,ti OR **dodekanisa**:ab,ti OR **'mount athos'**:ab,ti OR **'omicronros alphathos'**:ab,ti OR **athens**:ab,ti OR **athina**:ab,ti OR **thessaloniki**:ab,ti OR **thessalonica**:ab,ti OR **patras**:ab,ti OR **patra**:ab,ti OR **heraklion**:ab,ti OR **heraclion**:ab,ti OR **iraklion**:ab,ti OR **irakleion**:ab,ti OR **iraklio**:ab,ti OR **larissa**:ab,ti OR **larisa**:ab,ti OR **volos**:ab,ti OR **rhodes**:ab,ti OR **rodos**:ab,ti OR **ioannina**:ab,ti OR **janina**:ab,ti OR **yannena**:ab,ti OR **chania**:ab,ti OR **chalcis**:ab,ti OR **chalkida**:ab,ti OR **alexandroupoli**:ab,ti OR **german***:ad OR **deutschland**:ad OR **deutsch***:ad OR **bundesrepublik**:ad OR **westdeutschland**:ad OR **ostdeutschland**:ad OR **baden**:ad OR **wuerttemberg**:ad OR **wurttemberg**:ad OR **bayern**:ad OR **bavaria**:ad OR **berlin**:ad OR **brandenburg**:ad OR **bremen**:ad OR **hamburg**:ad OR **hessen**:ad OR **hesse**:ad OR **hessia**:ad OR **mecklenburg**:ad OR **vorpommern**:ad OR **pomerania**:ad OR **niedersachsen**:ad OR **neddersassen**:ad OR **saxony**:ad OR **niederbayern**:ad OR **'north rhine'**:ad OR **westphalia**:ad OR **westfalen**:ad OR **'rhineland palatinate'**:ad OR **'rheinland pfalz'**:ad OR **saarland**:ad OR **sachsen**:ad OR **'schleswig holstein'**:ad OR **thuringia**:ad OR **thuringen**:ad OR **thueringen**:ad OR **freiburg**:ad OR **karlsruhe**:ad OR **calsruhe**:ad OR **stuttgart**:ad OR **tubingen**:ad OR **oberbayern**:ad OR **'upper palatinate'**:ad OR **oberpfalz**:ad OR **franken**:ad OR **franconia**:ad OR **oberfranken**:ad OR **mittelfranken**:ad OR **schwaben**:ad OR **unterfranken**:ad OR **swabia**:ad OR **darmstadt**:ad OR **giessen**:ad OR **kassel**:ad OR **arnsberg**:ad OR **cologne**:ad OR **koln**:ad OR **koeln**:ad OR **detmold**:ad OR **dusseldorf**:ad OR **duesseldorf**:ad OR **munster**:ad OR **muenster**:ad OR **munich**:ad OR **munchen**:ad OR **muenchen**:ad OR **frankfurt**:ad OR **dortmund**:ad OR **essen**:ad OR **nurnberg**:ad OR **nuernberg**:ad OR **nuremberg**:ad OR **hanover**:ad OR **hannover**:ad OR **leipzig**:ad OR **dresden**:ad OR **ruhrgebiet**:ad OR **revier**:ad OR **ruhrpott**:ad OR **pott**:ad OR **ruhr**:ad OR **german***:ab,ti OR **deutschland**:ab,ti OR **deutsch***:ab,ti OR **bundesrepublik**:ab,ti OR **westdeutschland**:ab,ti OR **ostdeutschland**:ab,ti OR **baden**:ab,ti OR **wuerttemberg**:ab,ti OR **wurttemberg**:ab,ti OR **bayern**:ab,ti OR **bavaria**:ab,ti OR **berlin**:ab,ti OR **brandenburg**:ab,ti OR **bremen**:ab,ti OR **hamburg**:ab,ti OR **hessen**:ab,ti OR **hesse**:ab,ti OR **hessia**:ab,ti OR **mecklenburg**:ab,ti OR **vorpommern**:ab,ti OR **pomerania**:ab,ti OR **niedersachsen**:ab,ti OR **neddersassen**:ab,ti OR **saxony**:ab,ti OR **niederbayern**:ab,ti OR **'north rhine'**:ab,ti OR **westphalia**:ab,ti OR **westfalen**:ab,ti OR **'rhineland palatinate'**:ab,ti OR **'rheinland pfalz'**:ab,ti OR **saarland**:ab,ti OR **sachsen**:ab,ti OR **'schleswig holstein'**:ab,ti OR **thuringia**:ab,ti OR **thuringen**:ab,ti OR **thueringen**:ab,ti OR **freiburg**:ab,ti OR **karlsruhe**:ab,ti OR **calsruhe**:ab,ti OR **stuttgart**:ab,ti OR **tubingen**:ab,ti OR **oberbayern**:ab,ti OR **'upper palatinate'**:ab,ti OR **oberpfalz**:ab,ti OR **franken**:ab,ti OR **franconia**:ab,ti OR **oberfranken**:ab,ti OR **mittelfranken**:ab,ti OR **schwaben**:ab,ti OR **unterfranken**:ab,ti OR **swabia**:ab,ti OR **darmstadt**:ab,ti OR **giessen**:ab,ti OR **kassel**:ab,ti OR **arnsberg**:ab,ti OR **cologne**:ab,ti OR **koln**:ab,ti OR **koeln**:ab,ti OR **detmold**:ab,ti OR **dusseldorf**:ab,ti OR **duesseldorf**:ab,ti OR **munster**:ab,ti OR **muenster**:ab,ti OR **munich**:ab,ti OR **munchen**:ab,ti OR **muenchen**:ab,ti OR **frankfurt**:ab,ti OR **dortmund**:ab,ti OR **essen**:ab,ti OR **nurnberg**:ab,ti OR **nuernberg**:ab,ti OR **nuremberg**:ab,ti OR **hanover**:ab,ti OR **hannover**:ab,ti OR **leipzig**:ab,ti OR **dresden**:ab,ti OR **ruhrgebiet**:ab,ti OR **revier**:ab,ti OR **ruhrpott**:ab,ti OR **pott**:ab,ti OR **ruhr**:ab,ti OR **france**:ad OR **french***:ad OR **francais**:ad OR **alsace**:ad OR **aquitaine**:ad OR **auvergne**:ad OR **brittany**:ad OR **bretagne**:ad OR **bourgogne**:ad OR **burgundy**:ad OR **'champagne ardenne'**:ad OR **'franche comte'**:ad OR **'ile de france'**:ad OR **'languedoc roussillon'**:ad OR **limousin**:ad OR **lorraine**:ad OR **normandie**:ad OR **normandy**:ad OR **'midi pyrenees'**:ad OR **'nord pas de calais'**:ad OR **picardie**:ad OR **picardy**:ad OR **'poitou charentes'**:ad OR **provence**:ad OR **'rhone alpes'**:ad OR **corse**:ad OR **corsica**:ad OR **guiana**:ad OR **guyane**:ad OR **guadeloupe**:ad OR **martinique**:ad OR **reunion**:ad OR **mayotte**:ad OR **ain**:ad OR **aisne**:ad OR **allier**:ad OR **'alpes de haute provence'**:ad OR **'haute alpes'**:ad OR **'alpes maritimes'**:ad OR **ardeche**:ad OR **ardennes**:ad OR **ariege**:ad OR **aube**:ad OR **aude**:ad OR **aveyron**:ad OR **'bas rhin'**:ad OR **'bouches du rhone'**:ad OR **calvados**:ad OR **cantal**:ad OR **charente**:ad OR **cher**:ad OR **correze**:ad OR **'corse du sud'**:ad OR **'cote d or'**:ad OR **'cotes d armor'**:ad OR **'cote d azur'**:ad OR **creuse**:ad OR **'deux sevres'**:ad OR **dordogne**:ad OR **doubs**:ad OR **drome**:ad OR **essonne**:ad OR **eure**:ad OR **finistere**:ad OR **gard**:ad OR **gers**:ad OR **gironde**:ad OR **'haute corse'**:ad OR **'haute garonne'**:ad OR **'haute marne'**:ad OR **'hautes alpes'**:ad OR **'haute saone'**:ad OR **'haute savoie'**:ad OR **'hautes pyrenees'**:ad OR **'haute vienne'**:ad OR **'haut rhin'**:ad OR **'hauts de seine'**:ad OR **herault**:ad OR **'ille et vilaine'**:ad OR **indre**:ad OR **isere**:ad OR **jura**:ad OR **landes**:ad OR **loire**:ad OR **loiret**:ad OR (**lot**:ad AND (**departement**:ad OR **department**:ad)) OR **'lot et garonne'**:ad OR **'loir et cher'**:ad OR **lozere**:ad OR **manche**:ad OR **marne**:ad OR **mayenne**:ad OR **'meurthe et moselle'**:ad OR **meuse**:ad OR **morbihan**:ad OR **moselle**:ad OR (**nord**:ad AND (**department**:ad OR **departement**:ad)) OR **nievre**:ad OR **oise**:ad OR **orne**:ad OR **'pas de calais'**:ad OR **paris**:ad OR **'puy de dome'**:ad OR **'pyrenees atlantiques'**:ad OR **'pyrenees orientales'**:ad OR **rhone**:ad OR **sarthe**:ad OR **savoie**:ad OR **'seine et marne'**:ad OR **'seine maritime'**:ad OR **somme**:ad OR **tarn**:ad OR **'territoire de belfort'**:ad OR **'val de marne'**:ad OR **'val d oise'**:ad OR **var**:ad OR **vaucluse**:ad OR **vendee**:ad OR **vienne**:ad OR **vosges**:ad OR **yonne**:ad OR **yvelines**:ad OR **marseille**:ad OR **lyon**:ad OR **nice**:ad OR **nantes**:ad OR **strasbourg**:ad OR **montpellier**:ad OR **bordeaux**:ad OR **lille**:ad OR **toulouse**:ad OR **france**:ab,ti OR **french***:ab,ti OR **francais**:ab,ti OR **alsace**:ab,ti OR **aquitaine**:ab,ti OR **auvergne**:ab,ti OR **brittany**:ab,ti OR **bretagne**:ab,ti OR **bourgogne**:ab,ti OR **burgundy**:ab,ti OR **'champagne ardenne'**:ab,ti OR **'franche comte'**:ab,ti OR **'ile de france'**:ab,ti OR **'languedoc roussillon'**:ab,ti OR **limousin**:ab,ti OR **lorraine**:ab,ti OR **normandie**:ab,ti OR **normandy**:ab,ti OR **'midi pyrenees'**:ab,ti OR **'nord pas de calais'**:ab,ti OR **picardie**:ab,ti OR **picardy**:ab,ti OR **'poitou charentes'**:ab,ti OR **provence**:ab,ti OR **'rhone alpes'**:ab,ti OR **corse**:ab,ti OR **corsica**:ab,ti OR **guiana**:ab,ti OR **guyane**:ab,ti OR **guadeloupe**:ab,ti OR **martinique**:ab,ti OR **reunion**:ab,ti OR **mayotte**:ab,ti OR **ain**:ab,ti OR **aisne**:ab,ti OR **allier**:ab,ti OR **'alpes de haute provence'**:ab,ti OR **'haute alpes'**:ab,ti OR **'alpes maritimes'**:ab,ti OR **ardeche**:ab,ti OR **ardennes**:ab,ti OR **ariege**:ab,ti OR **aube**:ab,ti OR **aude**:ab,ti OR **aveyron**:ab,ti OR **'bas rhin'**:ab,ti OR **'bouches du rhone'**:ab,ti OR **calvados**:ab,ti OR **cantal**:ab,ti OR **charente**:ab,ti OR **cher**:ab,ti OR **correze**:ab,ti OR **'corse du sud'**:ab,ti OR **'cote d or'**:ab,ti OR **'cotes d armor'**:ab,ti OR **'cote d azur'**:ab,ti OR **creuse**:ab,ti OR **'deux sevres'**:ab,ti OR **dordogne**:ab,ti OR **doubs**:ab,ti OR **drome**:ab,ti OR **essonne**:ab,ti OR **eure**:ab,ti OR **finistere**:ab,ti OR **gard**:ab,ti OR **gers**:ab,ti OR **gironde**:ab,ti OR **'haute corse'**:ab,ti OR **'haute garonne'**:ab,ti OR **'haute marne'**:ab,ti OR **'hautes alpes'**:ab,ti OR **'haute saone'**:ab,ti OR **'haute savoie'**:ab,ti OR **'hautes pyrenees'**:ab,ti OR **'haute vienne'**:ab,ti OR **'haut rhin'**:ab,ti OR **'hauts de seine'**:ab,ti OR **herault**:ab,ti OR **'ille et vilaine'**:ab,ti OR **indre**:ab,ti OR **isere**:ab,ti OR **jura**:ab,ti OR **landes**:ab,ti OR **loire**:ab,ti OR **loiret**:ab,ti OR (**lot**:ab,ti AND (**departement**:ab,ti OR **department**:ab,ti)) OR **'lot et garonne'**:ab,ti OR **'loir et cher'**:ab,ti OR **lozere**:ab,ti OR **manche**:ab,ti OR **marne**:ab,ti OR **mayenne**:ab,ti OR **'meurthe et moselle'**:ab,ti OR **meuse**:ab,ti OR **morbihan**:ab,ti OR **moselle**:ab,ti OR (**nord**:ab,ti AND (**department**:ab,ti OR **departement**:ab,ti)) OR **nievre**:ab,ti OR **oise**:ab,ti OR **orne**:ab,ti OR **'pas de calais'**:ab,ti OR **paris**:ab,ti OR **'puy de dome'**:ab,ti OR **'pyrenees atlantiques'**:ab,ti OR **'pyrenees orientales'**:ab,ti OR **rhone**:ab,ti OR **sarthe**:ab,ti OR **savoie**:ab,ti OR **'seine et marne'**:ab,ti OR **'seine maritime'**:ab,ti OR **somme**:ab,ti OR **tarn**:ab,ti OR **'territoire de belfort'**:ab,ti OR **'val de marne'**:ab,ti OR **'val d oise'**:ab,ti OR **var**:ab,ti OR **vaucluse**:ab,ti OR **vendee**:ab,ti OR **vienne**:ab,ti OR **vosges**:ab,ti OR **yonne**:ab,ti OR **yvelines**:ab,ti OR **marseille**:ab,ti OR **lyon**:ab,ti OR **nice**:ab,ti OR **nantes**:ab,ti OR **strasbourg**:ab,ti OR **montpellier**:ab,ti OR **bordeaux**:ab,ti OR **lille**:ab,ti OR **toulouse**:ab,ti OR **finland**:ab,ti OR **finnish***:ab,ti OR **suomi***:ab,ti OR **lapland**:ab,ti OR **lappi**:ab,ti OR **lappland**:ab,ti OR **ostrobothnia**:ab,ti OR **pohjanmaa**:ab,ti OR **osterbotten**:ab,ti OR **kainuu**:ab,ti OR **kajanaland***:ab,ti OR **karelia**:ab,ti OR **karjala**:ab,ti OR **karelen**:ab,ti OR **savonia**:ab,ti OR **savo**:ab,ti OR **savolax**:ab,ti OR **pirkanmaa**:ab,ti OR **birkaland**:ab,ti OR **satakunta**:ab,ti OR **satakunda**:ab,ti OR **tavastia**:ab,ti OR **tavastland**:ab,ti OR **'paijat hame'**:ab,ti OR **'kanta hame'**:ab,ti OR **uusimaa**:ab,ti OR **nyland**:ab,ti OR **kymenlaakso**:ab,ti OR **kymmenedalen**:ab,ti OR **aland**:ab,ti OR **ahvenanmaa**:ab,ti OR **helsinki**:ab,ti OR **helsingfors**:ab,ti OR **espoo**:ab,ti OR **esbo**:ab,ti OR **tampere**:ab,ti OR **tammerfors**:ab,ti OR **vantaa**:ab,ti OR **vanda**:ab,ti OR **oulu**:ab,ti OR **uleaborg**:ab,ti OR **turku**:ab,ti OR **abo**:ab,ti OR **jyvaskyla**:ab,ti OR **kuopio**:ab,ti OR **lathi**:ab,ti OR **lahtis**:ab,ti OR **kouvola**:ab,ti OR **finland**:ad OR **finnish***:ad OR **suomi***:ad OR **lapland**:ad OR **lappi**:ad OR **lappland**:ad OR **ostrobothnia**:ad OR **pohjanmaa**:ad OR **osterbotten**:ad OR **kainuu**:ad OR **kajanaland***:ad OR **karelia**:ad OR **karjala**:ad OR **karelen**:ad OR **savonia**:ad OR **savo**:ad OR **savolax**:ad OR **pirkanmaa**:ad OR **birkaland**:ad OR **satakunta**:ad OR **satakunda**:ad OR **tavastia**:ad OR **tavastland**:ad OR **'paijat hame'**:ad OR **'kanta hame'**:ad OR **uusimaa**:ad OR **nyland**:ad OR **kymenlaakso**:ad OR **kymmenedalen**:ad OR **aland**:ad OR **ahvenanmaa**:ad OR **helsinki**:ad OR **helsingfors**:ad OR **espoo**:ad OR **esbo**:ad OR **tampere**:ad OR **tammerfors**:ad OR **vantaa**:ad OR **vanda**:ad OR **oulu**:ad OR **uleaborg**:ad OR **turku**:ad OR **abo**:ad OR **jyvaskyla**:ad OR **kuopio**:ad OR **lathi**:ad OR **lahtis**:ad OR **kouvola**:ad OR **estonia***:ab,ti OR **eesti**:ab,ti OR **esti**:ab,ti OR **tallinn**:ab,ti OR **harju**:ab,ti OR **harjumaa**:ab,ti OR **hiiu**:ab,ti OR **hiiumaa**:ab,ti OR **'ida viru'**:ab,ti OR **'ida virumaa'**:ab,ti OR **jarvamaa**:ab,ti OR **jarva**:ab,ti OR **jogevamaa**:ab,ti OR **jogeva**:ab,ti OR **laanemma**:ab,ti OR **laane**:ab,ti OR **parnumaa**:ab,ti OR **polva**:ab,ti OR **polvamaa**:ab,ti OR **rapla**:ab,ti OR **raplamaa**:ab,ti OR **saare**:ab,ti OR **saaremaa**:ab,ti OR **tartu**:ab,ti OR **tartumaa**:ab,ti OR **valga**:ab,ti OR **valgamaa**:ab,ti OR **valgamaakond**:ab,ti OR **viljandimaa**:ab,ti OR **voru**:ab,ti OR **vorumaa**:ab,ti OR **narva**:ab,ti OR **parnu**:ab,ti OR **'kohtla jarve'**:ab,ti OR **viljandi**:ab,ti OR **rakvere**:ab,ti OR **maardu**:ab,ti OR **sillamae**:ab,ti OR **kuressaare**:ab,ti OR **estonia***:ad OR **eesti**:ad OR **esti**:ad OR **tallinn**:ad OR **harju**:ad OR **harjumaa**:ad OR **hiiu**:ad OR **hiiumaa**:ad OR **'ida viru'**:ad OR **'ida virumaa'**:ad OR **jarvamaa**:ad OR **jarva**:ad OR **jogevamaa**:ad OR **jogeva**:ad OR **laanemma**:ad OR **laane**:ad OR **parnumaa**:ad OR **polva**:ad OR **polvamaa**:ad OR **rapla**:ad OR **raplamaa**:ad OR **saare**:ad OR **saaremaa**:ad OR **tartu**:ad OR **tartumaa**:ad OR **valga**:ad OR **valgamaa**:ad OR **valgamaakond**:ad OR **viljandimaa**:ad OR **voru**:ad OR **vorumaa**:ad OR **narva**:ad OR **parnu**:ad OR **'kohtla jarve'**:ad OR **viljandi**:ad OR **rakvere**:ad OR **maardu**:ad OR **sillamae**:ad OR **kuressaare**:ad \|  \| \| #1.5 \| **romania**:ab,ti OR **rumania**:ab,ti OR **roumania**:ab,ti OR **romanian**:ab,ti OR **roman**:ab,ti OR **bucharest**:ab,ti OR **bucuresti**:ab,ti OR **alba**:ab,ti OR **brasov**:ab,ti OR **covasna**:ab,ti OR **harghita**:ab,ti OR **mures**:ab,ti OR **sibiu**:ab,ti OR **bacau**:ab,ti OR **botosani**:ab,ti OR **iasi**:ab,ti OR **neamt**:ab,ti OR **suceava**:ab,ti OR **vaslui**:ab,ti OR **bihor**:ab,ti OR **'bistrita nasaud'**:ab,ti OR **cluj**:ab,ti OR **maramures**:ab,ti OR **salaj**:ab,ti OR **'satu mare'**:ab,ti OR **arges**:ab,ti OR **calarasi**:ab,ti OR **dambovita**:ab,ti OR **giurgiu**:ab,ti OR **ialomita**:ab,ti OR **prahova**:ab,ti OR **teleorman**:ab,ti OR **braila**:ab,ti OR **buzau**:ab,ti OR **galati**:ab,ti OR **tulcea**:ab,ti OR **vrancea**:ab,ti OR **dolj**:ab,ti OR **gorj**:ab,ti OR **mehedinti**:ab,ti OR (**olt**:ab,ti AND (**river**:ab,ti OR **county**:ab,ti OR **region**:ab,ti OR **judetul**:ab,ti OR **raul**:ab,ti)) OR **valcea**:ab,ti OR **vilcea**:ab,ti OR **arad**:ab,ti OR **'caras-severin'**:ab,ti OR **hunedoara**:ab,ti OR **timis**:ab,ti OR **ilfov**:ab,ti OR **timisoara**:ab,ti OR **constanta**:ab,ti OR **craiova**:ab,ti OR **ploiesti**:ab,ti OR **oradea**:ab,ti OR **'cluj-napoca'**:ab,ti OR **deva**:ab,ti OR **romania**:ad OR **rumania**:ad OR **roumania**:ad OR **romanian**:ad OR **roman**:ad OR **bucharest**:ad OR **bucuresti**:ad OR **alba**:ad OR **brasov**:ad OR **covasna**:ad OR **harghita**:ad OR **mures**:ad OR **sibiu**:ad OR **bacau**:ad OR **botosani**:ad OR **iasi**:ad OR **neamt**:ad OR **suceava**:ad OR **vaslui**:ad OR **bihor**:ad OR **'bistrita nasaud'**:ad OR **cluj**:ad OR **maramures**:ad OR **salaj**:ad OR **'satu mare'**:ad OR **arges**:ad OR **calarasi**:ad OR **dambovita**:ad OR **giurgiu**:ad OR **ialomita**:ad OR **prahova**:ad OR **teleorman**:ad OR **braila**:ad OR **buzau**:ad OR **galati**:ad OR **tulcea**:ad OR **vrancea**:ad OR **dolj**:ad OR **gorj**:ad OR **mehedinti**:ad OR (**olt**:ad AND (**river**:ad OR **county**:ad OR **region**:ad OR **judetul**:ad OR **raul**:ad)) OR **valcea**:ad OR **vilcea**:ad OR **arad**:ad OR **'caras-severin'**:ad OR **hunedoara**:ad OR **timis**:ad OR **ilfov**:ad OR **timisoara**:ad OR **constanta**:ad OR **craiova**:ad OR **ploiesti**:ad OR **oradea**:ad OR **'cluj-napoca'**:ad OR **deva**:ad OR **portugal**:ab,ti OR **portugues***:ab,ti OR **lisboa**:ab,ti OR **lisbon**:ab,ti OR **leira**:ab,ti OR **santarem**:ab,ti OR **beja**:ab,ti OR **faro**:ab,ti OR **evora**:ab,ti OR **portalegre**:ab,ti OR **'castelo branco'**:ab,ti OR **guarda**:ab,ti OR **aveiro**:ab,ti OR **viseu**:ab,ti OR **braganca**:ab,ti OR **'vila real'**:ab,ti OR **'viana do castelo'**:ab,ti OR **alentejo**:ab,ti OR **azores**:ab,ti OR **acores**:ab,ti OR **madeira**:ab,ti OR **'os montes'**:ab,ti OR (**ave**:ab,ti AND (**community**:ab,ti OR **intermunicipal**:ab,ti OR **comunidade**:ab,ti)) OR **mondego**:ab,ti OR **vouga**:ab,ti OR **beira**:ab,ti OR **cavado**:ab,ti OR **lafoes**:ab,ti OR **douro**:ab,ti OR **porto**:ab,ti OR **tejo**:ab,ti OR **minho**:ab,ti OR **setubal**:ab,ti OR **pinhal**:ab,ti OR **'serra da estrela'**:ab,ti OR **tamega**:ab,ti OR **algarve**:ab,ti OR **gaia**:ab,ti OR **amadora**:ab,ti OR **braga**:ab,ti OR (**agualva**:ab,ti AND **cacem**:ab,ti) OR **funchal**:ab,ti OR **coimbra**:ab,ti OR **almada**:ab,ti OR **portugal**:ad OR **portugues***:ad OR **lisboa**:ad OR **lisbon**:ad OR **leira**:ad OR **santarem**:ad OR **beja**:ad OR **faro**:ad OR **evora**:ad OR **portalegre**:ad OR **'castelo branco'**:ad OR **guarda**:ad OR **aveiro**:ad OR **viseu**:ad OR **braganca**:ad OR **'vila real'**:ad OR **'viana do castelo'**:ad OR **alentejo**:ad OR **azores**:ad OR **acores**:ad OR **madeira**:ad OR **'os montes'**:ad OR (**ave**:ad AND (**community**:ad OR **intermunicipal**:ad OR **comunidade**:ad)) OR **mondego**:ad OR **vouga**:ad OR **beira**:ad OR **cavado**:ad OR **lafoes**:ad OR **douro**:ad OR **porto**:ad OR **tejo**:ad OR **minho**:ad OR **setubal**:ad OR **pinhal**:ad OR **'serra da estrela'**:ad OR **tamega**:ad OR **algarve**:ad OR **gaia**:ad OR **amadora**:ad OR **braga**:ad OR (**agualva**:ad AND **cacem**:ad) OR **funchal**:ad OR **coimbra**:ad OR **almada**:ad OR **poland**:ad OR **polska**:ad OR **polish**:ad OR **polski**:ad OR **pole**:ad OR **poles**:ad OR **polak**:ad OR **polka**:ad OR **polacy**:ad OR **warsaw**:ad OR **warszawa**:ad OR **wielkopolskie**:ad OR **pomerania***:ad OR **pomorskie**:ad OR **kuyavian**:ad OR **kujawsko**:ad OR **malopolskie**:ad OR **lodz**:ad OR **lodzkie**:ad OR **silesia***:ad OR **dolnoslaskie**:ad OR **lublin**:ad OR **lubelskie**:ad OR **lubus**:ad OR **lubusz**:ad OR **lubuskie**:ad OR **masovia**:ad OR **mazowske**:ad OR **masovian**:ad OR **mazowieckie**:ad OR **opole**:ad OR **opolskie**:ad OR **podlaskie**:ad OR **podlachia**:ad OR **podlasie**:ad OR **subcarpathian***:ad OR **carpathian***:ad OR **podkarpackie**:ad OR **swietokrzyskie**:ad OR **slaskie**:ad OR **slask**:ad OR **'varmia mazuria'**:ad OR **'varmian mazurian'**:ad OR **'varmia masuria'**:ad OR **'varmian masurian'**:ad OR **'warmia mazury'**:ad OR **'warminsko mazurskie'**:ad OR **zachodniopomorskie**:ad OR **krakow**:ad OR **cracow**:ad OR **wroclaw**:ad OR **poznan**:ad OR **gdansk**:ad OR **szczecin**:ad OR **bydgoszcz**:ad OR **katowice**:ad OR **bialystok**:ad OR **olsztyn**:ad OR **kielce**:ad OR **'zielona gora'**:ad OR **torun**:ad OR **'gorzow wielkopolski'**:ad OR **poland**:ab,ti OR **polska**:ab,ti OR **polish**:ab,ti OR **polski**:ab,ti OR **pole**:ab,ti OR **poles**:ab,ti OR **polak**:ab,ti OR **polka**:ab,ti OR **polacy**:ab,ti OR **warsaw**:ab,ti OR **warszawa**:ab,ti OR **wielkopolskie**:ab,ti OR **pomerania***:ab,ti OR **pomorskie**:ab,ti OR **kuyavian**:ab,ti OR **kujawsko**:ab,ti OR **malopolskie**:ab,ti OR **lodz**:ab,ti OR **lodzkie**:ab,ti OR **silesia***:ab,ti OR **dolnoslaskie**:ab,ti OR **lublin**:ab,ti OR **lubelskie**:ab,ti OR **lubus**:ab,ti OR **lubusz**:ab,ti OR **lubuskie**:ab,ti OR **masovia**:ab,ti OR **mazowske**:ab,ti OR **masovian**:ab,ti OR **mazowieckie**:ab,ti OR **opole**:ab,ti OR **opolskie**:ab,ti OR **podlaskie**:ab,ti OR **podlachia**:ab,ti OR **podlasie**:ab,ti OR **subcarpathian***:ab,ti OR **carpathian***:ab,ti OR **podkarpackie**:ab,ti OR **swietokrzyskie**:ab,ti OR **slaskie**:ab,ti OR **slask**:ab,ti OR **'varmia mazuria'**:ab,ti OR **'varmian mazurian'**:ab,ti OR **'varmia masuria'**:ab,ti OR **'varmian masurian'**:ab,ti OR **'warmia mazury'**:ab,ti OR **'warminsko mazurskie'**:ab,ti OR **zachodniopomorskie**:ab,ti OR **krakow**:ab,ti OR **cracow**:ab,ti OR **wroclaw**:ab,ti OR **poznan**:ab,ti OR **gdansk**:ab,ti OR **szczecin**:ab,ti OR **bydgoszcz**:ab,ti OR **katowice**:ab,ti OR **bialystok**:ab,ti OR **olsztyn**:ab,ti OR **kielce**:ab,ti OR **'zielona gora'**:ab,ti OR **torun**:ab,ti OR **'gorzow wielkopolski'**:ab,ti OR **netherlands**:ad OR **nederland***:ad OR **dutch***:ad OR **amsterdam**:ad OR **drenthe**:ad OR **flevoland**:ad OR **friesland**:ad OR **fryslan**:ad OR **gelderland**:ad OR **guelders**:ad OR **groningen**:ad OR **limburg**:ad OR **'north brabant'**:ad OR **'noord brabant'**:ad OR **holland**:ad OR **overijssel**:ad OR **overissel**:ad OR **utrecht**:ad OR **zeeland**:ad OR **rotterdam**:ad OR **hague**:ad OR **eindhoven**:ad OR **tilburg**:ad OR **almere**:ad OR **breda**:ad OR **nijmegen**:ad OR **nimeguen**:ad OR **netherlands**:ab,ti OR **nederland***:ab,ti OR **dutch***:ab,ti OR **amsterdam**:ab,ti OR **drenthe**:ab,ti OR **flevoland**:ab,ti OR **friesland**:ab,ti OR **fryslan**:ab,ti OR **gelderland**:ab,ti OR **guelders**:ab,ti OR **groningen**:ab,ti OR **limburg**:ab,ti OR **'north brabant'**:ab,ti OR **'noord brabant'**:ab,ti OR **holland**:ab,ti OR **overijssel**:ab,ti OR **overissel**:ab,ti OR **utrecht**:ab,ti OR **zeeland**:ab,ti OR **rotterdam**:ab,ti OR **hague**:ab,ti OR **eindhoven**:ab,ti OR **tilburg**:ab,ti OR **almere**:ab,ti OR **breda**:ab,ti OR **nijmegen**:ab,ti OR **nimeguen**:ab,ti OR **malta**:ab,ti OR **maltese**:ab,ti OR **valletta**:ab,ti OR **gozo**:ab,ti OR **ghawdex**:ab,ti OR **malta**:ad OR **maltese**:ad OR **valletta**:ad OR **gozo**:ad OR **ghawdex**:ad OR **luxembourg***:ab,ti OR **luxemburg**:ab,ti OR **letzebuerg**:ab,ti OR **diekirch**:ab,ti OR **grevenmacher**:ab,ti OR **luxembourg***:ad OR **luxemburg**:ad OR **letzebuerg**:ad OR **diekirch**:ad OR **grevenmacher**:ad OR **lithuania***:ab,ti OR **'lietuvos respublika'**:ab,ti OR **lietuva**:ab,ti OR **lietuviu**:ab,ti OR **vilnius**:ab,ti OR **vilniaus**:ab,ti OR **kaunas**:ab,ti OR **kauno**:ab,ti OR **klaipeda**:ab,ti OR **klaipedos**:ab,ti OR **panevezys**:ab,ti OR **panevezio**:ab,ti OR **siauliai**:ab,ti OR **siauliu**:ab,ti OR **alytus**:ab,ti OR **alytaus**:ab,ti OR **taurages**:ab,ti OR **taurage**:ab,ti OR **marijampoles**:ab,ti OR **marijampole**:ab,ti OR **telsiu**:ab,ti OR **telsiai**:ab,ti OR **utenos**:ab,ti OR **utena**:ab,ti OR **mazeikiai**:ab,ti OR **jonava**:ab,ti OR **mazeikiu**:ab,ti OR **jonavos**:ab,ti OR **lithuania***:ad OR **'lietuvos respublika'**:ad OR **lietuva**:ad OR **lietuviu**:ad OR **vilnius**:ad OR **vilniaus**:ad OR **kaunas**:ad OR **kauno**:ad OR **klaipeda**:ad OR **klaipedos**:ad OR **panevezys**:ad OR **panevezio**:ad OR **siauliai**:ad OR **siauliu**:ad OR **alytus**:ad OR **alytaus**:ad OR **taurages**:ad OR **taurage**:ad OR **marijampoles**:ad OR **marijampole**:ad OR **telsiu**:ad OR **telsiai**:ad OR **utenos**:ad OR **utena**:ad OR **mazeikiai**:ad OR **jonava**:ad OR **mazeikiu**:ad OR **jonavos**:ad OR **latvi***:ab,ti OR **latvija***:ab,ti OR **riga**:ab,ti OR **courland**:ab,ti OR **kurzeme**:ab,ti OR **kurland**:ab,ti OR **latgale**:ab,ti OR **lettgallia**:ab,ti OR **latgola**:ab,ti OR **vidzeme**:ab,ti OR **vidumo**:ab,ti OR **semigallia**:ab,ti OR **semigalia**:ab,ti OR **zemgale**:ab,ti OR **pieriga**:ab,ti OR **daugavpils**:ab,ti OR **dinaburg**:ab,ti OR **liepaja**:ab,ti OR **libau**:ab,ti OR **jelgava**:ab,ti OR **jurmala**:ab,ti OR **jekabpils**:ab,ti OR **jakobstadt**:ab,ti OR **rezekne**:ab,ti OR **rezne**:ab,ti OR **rositten**:ab,ti OR **valmiera**:ab,ti OR **wolmar**:ab,ti OR **ventspils**:ab,ti OR **windau**:ab,ti OR **latvi***:ad OR **latvija***:ad OR **riga**:ad OR **courland**:ad OR **kurzeme**:ad OR **kurland**:ad OR **latgale**:ad OR **lettgallia**:ad OR **latgola**:ad OR **vidzeme**:ad OR **vidumo**:ad OR **semigallia**:ad OR **semigalia**:ad OR **zemgale**:ad OR **pieriga**:ad OR **daugavpils**:ad OR **dinaburg**:ad OR **liepaja**:ad OR **libau**:ad OR **jelgava**:ad OR **jurmala**:ad OR **jekabpils**:ad OR **jakobstadt**:ad OR **rezekne**:ad OR **rezne**:ad OR **rositten**:ad OR **valmiera**:ad OR **wolmar**:ad OR **ventspils**:ad OR **windau**:ad \|  \| \| #1.4 \| **denmark**:ab,ti OR **danish***:ab,ti OR **danmark**:ab,ti OR **dansk***:ab,ti OR **hovedstaden**:ab,ti OR **midtjylland**:ab,ti OR **sjaelland**:ab,ti OR **sealand**:ab,ti OR **syddanmark**:ab,ti OR **jutland**:ab,ti OR **jylland**:ab,ti OR **nordjylland**:ab,ti OR **sonderjyllands**:ab,ti OR **'zealand region'**:ab,ti OR **'region zealand'**:ab,ti OR **hillerod**:ab,ti OR **viborg**:ab,ti OR **aalborg**:ab,ti OR **alborg**:ab,ti OR **soro**:ab,ti OR **vejle**:ab,ti OR **copenhagen**:ab,ti OR **kobenhavn**:ab,ti OR **arhus**:ab,ti OR **aarhus**:ab,ti OR **roskilde**:ab,ti OR **odense**:ab,ti OR **frederiksberg**:ab,ti OR **esbjerg**:ab,ti OR **gentofte**:ab,ti OR **gladsaxe**:ab,ti OR **randers**:ab,ti OR **kolding**:ab,ti OR **denmark**:ad OR **danish***:ad OR **danmark**:ad OR **dansk***:ad OR **hovedstaden**:ad OR **midtjylland**:ad OR **sjaelland**:ad OR **sealand**:ad OR **syddanmark**:ad OR **jutland**:ad OR **jylland**:ad OR **nordjylland**:ad OR **sonderjyllands**:ad OR **'zealand region'**:ad OR **'region zealand'**:ad OR **hillerod**:ad OR **viborg**:ad OR **aalborg**:ad OR **alborg**:ad OR **soro**:ad OR **vejle**:ad OR **copenhagen**:ad OR **kobenhavn**:ad OR **arhus**:ad OR **aarhus**:ad OR **roskilde**:ad OR **odense**:ad OR **frederiksberg**:ad OR **esbjerg**:ad OR **gentofte**:ad OR **gladsaxe**:ad OR **randers**:ad OR **kolding**:ad OR **czech***:ab,ti OR **cesk***:ab,ti OR **stredoces***:ab,ti OR **jihoces***:ab,ti OR **bohemia**:ab,ti OR **'bohemian region'**:ab,ti OR **kralovehradec***:ab,ti OR **'hradec kralove'**:ab,ti OR **karlovars***:ab,ti OR **'karlovy vary'**:ab,ti OR **liberec***:ab,ti OR **moravskoslezs***:ab,ti OR **'moravian silesian'**:ab,ti OR **olomouc***:ab,ti OR **pardubic***:ab,ti OR **plzen***:ab,ti OR **pilsen**:ab,ti OR **prage**:ab,ti OR **praha**:ab,ti OR **prag**:ab,ti OR **jihomorav***:ab,ti OR **moravia**:ab,ti OR **moravian**:ab,ti OR **morava**:ab,ti OR **usteck***:ab,ti OR **usti**:ab,ti OR **vysocina**:ab,ti OR **zlin**:ab,ti OR **zlinsk***:ab,ti OR **'ceske budejovice'**:ab,ti OR **budweis**:ab,ti OR **brno**:ab,ti OR **ostrava**:ab,ti OR **czech***:ad OR **cesk***:ad OR **stredoces***:ad OR **jihoce***:ad OR **bohemia**:ad OR **'bohemian region'**:ad OR **kralovehradec***:ad OR **'hradec kralove'**:ad OR **karlovars***:ad OR **'karlovy vary'**:ad OR **liberec***:ad OR **moravskoslezsk***:ad OR **'moravian silesian'**:ad OR **olomouc***:ad OR **pardubic***:ad OR **pardubice**:ad OR **plzen***:ad OR **pilsen**:ad OR **prage**:ad OR **praha**:ad OR **prag**:ad OR **jihomorav***:ad OR **moravia**:ad OR **moravian**:ad OR **morava**:ad OR **usteck***:ad OR **usti**:ad OR **vysocina**:ad OR **zlin**:ad OR **zlinsk***:ad OR **'ceske budejovice'**:ad OR **budweis**:ad OR **brno**:ad OR **ostrava**:ad OR **cyprus**:ab,ti OR **cypriot***:ab,ti OR **kypros**:ab,ti OR **kibris**:ab,ti OR **kypriaki***:ab,ti OR **nicosia**:ab,ti OR **lefkosa**:ab,ti OR **lefkosia**:ab,ti OR **famagusta**:ab,ti OR **magusa**:ab,ti OR **ammochostos**:ab,ti OR **gazimagusa**:ab,ti OR **kyrenia**:ab,ti OR **girne**:ab,ti OR **keryneia**:ab,ti OR **larnaca**:ab,ti OR **larnaka**:ab,ti OR **limassol**:ab,ti OR **lemesos**:ab,ti OR **limasol**:ab,ti OR **leymosun**:ab,ti OR **paphos**:ab,ti OR **pafos**:ab,ti OR **baf**:ab,ti OR **gazibaf**:ab,ti OR **protaras**:ab,ti OR **pergamos**:ab,ti OR **beyarmudu**:ab,ti OR **morfou**:ab,ti OR **guzelyurt**:ab,ti OR **omorfo**:ab,ti OR **morphou**:ab,ti OR **aradippou**:ab,ti OR **cyprus**:ad OR **cypriot***:ad OR **kypros**:ad OR **kibris**:ad OR **kypriaki***:ad OR **nicosia**:ad OR **lefkosa**:ad OR **lefkosia**:ad OR **famagusta**:ad OR **magusa**:ad OR **ammochostos**:ad OR **gazimagusa**:ad OR **kyrenia**:ad OR **girne**:ad OR **keryneia**:ad OR **larnaca**:ad OR **larnaka**:ad OR **limassol**:ad OR **lemesos**:ad OR **limasol**:ad OR **leymosun**:ad OR **paphos**:ad OR **pafos**:ad OR **baf**:ad OR **gazibaf**:ad OR **protaras**:ad OR **pergamos**:ad OR **beyarmudu**:ad OR **morfou**:ad OR **guzelyurt**:ad OR **omorfo**:ad OR **morphou**:ad OR **aradippou**:ad OR **croatia***:ab,ti OR **hrvatsk***:ab,ti OR **hrvat**:ab,ti OR **bjelovar**:ab,ti OR **'bjelovarsko bilogorska'**:ab,ti OR **'brod posavina'**:ab,ti OR **'brodsko posavska'**:ab,ti OR **'dubrovnik neretva'**:ab,ti OR **'dubrovacko neretvanska'**:ab,ti OR **istria**:ab,ti OR **istarska**:ab,ti OR **karlovacka**:ab,ti OR **karlovac**:ab,ti OR **'koprivnicko krizevacka'**:ab,ti OR **koprivnica**:ab,ti OR **krizevci**:ab,ti OR **'krapina zagorje'**:ab,ti OR **'krapinsko zagorska'**:ab,ti OR **'lika senj'**:ab,ti OR **'licko senjska'**:ab,ti OR **medimurska**:ab,ti OR **medimurje**:ab,ti OR **osijek**:ab,ti OR **osjecko**:ab,ti OR **baranja**:ab,ti OR **'osjecko baranjska'**:ab,ti OR **'pozega slavonia'**:ab,ti OR **'pozesko slavonska'**:ab,ti OR **'primorje gorski kotar'**:ab,ti OR **'primorsko goranska'**:ab,ti OR **'sibensko kninska'**:ab,ti OR **'sibensko kninske'**:ab,ti OR **sibenik**:ab,ti OR **knin**:ab,ti OR **sisak**:ab,ti OR **'sisacko moslavacka'**:ab,ti OR **moslavina**:ab,ti OR **'splitsko dalmatinska'**:ab,ti OR **split**:ab,ti OR **dalmatia**:ab,ti OR **varazdin**:ab,ti OR **varazdinska**:ab,ti OR **'viroviticko-podravska'**:ab,ti OR **virovitica**:ab,ti OR **podravina**:ab,ti OR **'vukovarsko srijemska'**:ab,ti OR **vukovar**:ab,ti OR **srijem**:ab,ti OR **zadar**:ab,ti OR **zadarska**:ab,ti OR **zagreb**:ab,ti OR **zagrebacka**:ab,ti OR **rijeka**:ab,ti OR **'velika gorica'**:ab,ti OR **'slavonski brod'**:ab,ti OR **pula**:ab,ti OR **croatia***:ad OR **hrvatsk***:ad OR **hrvat**:ad OR **bjelovar**:ad OR **'bjelovarsko bilogorska'**:ad OR **'brod posavina'**:ad OR **'brodsko posavska'**:ad OR **'dubrovnik neretva'**:ad OR **'dubrovacko neretvanska'**:ad OR **istria**:ad OR **istarska**:ad OR **karlovacka**:ad OR **karlovac**:ad OR **'koprivnicko krizevacka'**:ad OR **koprivnica**:ad OR **krizevci**:ad OR **'krapina zagorje'**:ad OR **'krapinsko zagorska'**:ad OR **'lika senj'**:ad OR **'licko senjska'**:ad OR **medimurska**:ad OR **medimurje**:ad OR **osijek**:ad OR **osjecko**:ad OR **baranja**:ad OR **'osjecko baranjska'**:ad OR **'pozega slavonia'**:ad OR **'pozesko slavonska'**:ad OR **'primorje gorski kotar'**:ad OR **'primorsko goranska'**:ad OR **'sibensko kninska'**:ad OR **'sibensko kninske'**:ad OR **sibenik**:ad OR **knin**:ad OR **sisak**:ad OR **'sisacko moslavacka'**:ad OR **moslavina**:ad OR **'splitsko dalmatinska'**:ad OR **split**:ad OR **dalmatia**:ad OR **varazdin**:ad OR **varazdinska**:ad OR **'viroviticko-podravska'**:ad OR **virovitica**:ad OR **podravina**:ad OR **'vukovarsko srijemska'**:ad OR **vukovar**:ad OR **srijem**:ad OR **zadar**:ad OR **zadarska**:ad OR **zagreb**:ad OR **zagrebacka**:ad OR **rijeka**:ad OR **'velika gorica'**:ad OR **'slavonski brod'**:ad OR **pula**:ad OR **bulgaria***:ab,ti OR **sofia**:ab,ti OR **gabrovo**:ab,ti OR **blagoevgrad**:ab,ti OR **'pirin macedonia'**:ab,ti OR **burgas**:ab,ti OR **dobrich**:ab,ti OR **haskovo**:ab,ti OR **kardzhali**:ab,ti OR **kurdzhali**:ab,ti OR **kyustendil**:ab,ti OR **lovech**:ab,ti OR **montana**:ab,ti OR **pazardzhik**:ab,ti OR **pernik**:ab,ti OR **pleven**:ab,ti OR **plovdiv**:ab,ti OR **razgrad**:ab,ti OR **rousse**:ab,ti OR **ruse**:ab,ti OR **shumen**:ab,ti OR **sliven**:ab,ti OR **silistra**:ab,ti OR **smolyan**:ab,ti OR **'stara zagora'**:ab,ti OR **targovishte**:ab,ti OR **varna**:ab,ti OR **tarnovo**:ab,ti OR **vidin**:ab,ti OR **vratsa**:ab,ti OR **vratza**:ab,ti OR **yambol**:ab,ti OR **bulgaria***:ad OR **sofia**:ad OR **gabrovo**:ad OR **blagoevgrad**:ad OR **'pirin macedonia'**:ad OR **burgas**:ad OR **dobrich**:ad OR **haskovo**:ad OR **kardzhali**:ad OR **kurdzhali**:ad OR **kyustendil**:ad OR **lovech**:ad OR **montana**:ad OR **pazardzhik**:ad OR **pernik**:ad OR **pleven**:ad OR **plovdiv**:ad OR **razgrad**:ad OR **rousse**:ad OR **ruse**:ad OR **shumen**:ad OR **sliven**:ad OR **silistra**:ad OR **smolyan**:ad OR **'stara zagora'**:ad OR **targovishte**:ad OR **varna**:ad OR **tarnovo**:ad OR **vidin**:ad OR **vratsa**:ad OR **vratza**:ad OR **yambol**:ad OR **belgi***:ab,ti OR **belge**:ab,ti OR **belgisch**:ab,ti OR **brussel***:ab,ti OR **bruxelles**:ab,ti OR **bruxelloise**:ab,ti OR **flemish**:ab,ti OR **flamand**:ab,ti OR **flemisch**:ab,ti OR **flanders**:ab,ti OR **flandern**:ab,ti OR **flandre**:ab,ti OR **vlaanderen**:ab,ti OR **vlaams**:ab,ti OR **flamande**:ab,ti OR **waals**:ab,ti OR **walloon***:ab,ti OR **wallon***:ab,ti OR **antwerp***:ab,ti OR **anvers**:ab,ti OR **ostflandern**:ab,ti OR **'vlaams brabant'**:ab,ti OR **limbourg**:ab,ti OR **limburg**:ab,ti OR **hainault**:ab,ti OR **hainaut**:ab,ti OR **henegouwen**:ab,ti OR **hennegau**:ab,ti OR **liege**:ab,ti OR **luik**:ab,ti OR **luttich**:ab,ti OR **namur**:ab,ti OR **namen**:ab,ti OR **westflandern**:ab,ti OR **'waals brabant'**:ab,ti OR **ghent**:ab,ti OR **gent**:ab,ti OR **gand**:ab,ti OR **charleroi**:ab,ti OR **bruges**:ab,ti OR **brugge**:ab,ti OR **schaerbeek**:ab,ti OR **schaarbeek**:ab,ti OR **anderlecht**:ab,ti OR **leuven**:ab,ti OR **louvain**:ab,ti OR **hasselt**:ab,ti OR **mons**:ab,ti OR **wavre**:ab,ti OR **waver**:ab,ti OR **belgi***:ad OR **belge**:ad OR **belgisch**:ad OR **brussel***:ad OR **bruxelles**:ad OR **bruxelloise**:ad OR **flemish**:ad OR **flamand**:ad OR **flemisch**:ad OR **flanders**:ad OR **flandern**:ad OR **flandre**:ad OR **vlaanderen**:ad OR **vlaams**:ad OR **flamande**:ad OR **waals**:ad OR **walloon***:ad OR **wallon***:ad OR **antwerp***:ad OR **anvers**:ad OR **ostflandern**:ad OR **'vlaams brabant'**:ad OR **limbourg**:ad OR **limburg**:ad OR **hainault**:ad OR **hainaut**:ad OR **henegouwen**:ad OR **hennegau**:ad OR **liege**:ad OR **luik**:ad OR **luttich**:ad OR **namur**:ad OR **namen**:ad OR **westflandern**:ad OR **'waals brabant'**:ad OR **ghent**:ad OR **gent**:ad OR **gand**:ad OR **charleroi**:ad OR **bruges**:ad OR **brugge**:ad OR **schaerbeek**:ad OR **schaarbeek**:ad OR **anderlecht**:ad OR **leuven**:ad OR **louvain**:ad OR **hasselt**:ad OR **mons**:ad OR **wavre**:ad OR **waver**:ad OR **austria***:ab,ti OR **vienna**:ab,ti OR **wien**:ab,ti OR **osterreich***:ab,ti OR **sudosterreich**:ab,ti OR **westosterreich**:ab,ti OR **niederosterreich**:ab,ti OR **burgenland**:ab,ti OR **carinthia**:ab,ti OR **karinthia**:ab,ti OR **karnten**:ab,ti OR **oberosterreich**:ab,ti OR **styria**:ab,ti OR **steiermark**:ab,ti OR **salzburg**:ab,ti OR **saizburg**:ab,ti OR **tyrol**:ab,ti OR **tirol**:ab,ti OR **becs**:ab,ti OR **vorarlberg**:ab,ti OR **bregenz**:ab,ti OR **linz**:ab,ti OR **eisenstadt**:ab,ti OR **innsbruck**:ab,ti OR **graz**:ab,ti OR **klagenfurt**:ab,ti OR **polten**:ab,ti OR **villach**:ab,ti OR **wels**:ab,ti OR **dornbirn**:ab,ti OR **feldkirch**:ab,ti OR **steyr**:ab,ti OR **austria***:ad OR **vienna**:ad OR **wien**:ad OR **osterreich***:ad OR **sudosterreich**:ad OR **westosterreich**:ad OR **niederosterreich**:ad OR **burgenland**:ad OR **carinthia**:ad OR **karnten**:ad OR **oberosterreich**:ad OR **styria**:ad OR **steiermark**:ad OR **salzburg**:ad OR **saizburg**:ad OR **tyrol**:ad OR **tirol**:ad OR **becs**:ad OR **vorarlberg**:ad OR **bregenz**:ad OR **linz**:ad OR **eisenstadt**:ad OR **innsbruck**:ad OR **graz**:ad OR **klagenfurt**:ad OR **polten**:ad OR **villach**:ad OR **wels**:ad OR **dornbirn**:ad OR **feldkirch**:ad OR **steyr**:ad \|  \| \| #1.3 \| **iceland**:ab,ti OR **icelandic***:ab,ti OR **islenska***:ab,ti OR **icelander***:ab,ti OR **islendinga***:ab,ti OR **reykjavik**:ab,ti OR **reykjavikurborg**:ab,ti OR **hofudborgarsvaedid**:ab,ti OR **sudurnes**:ab,ti OR **vesturland**:ab,ti OR **vestfirdir**:ab,ti OR **westfjords**:ab,ti OR **nordurland**:ab,ti OR **austurland**:ab,ti OR **sudurland**:ab,ti OR **kopavogur**:ab,ti OR **hafnarfjordur**:ab,ti OR **iceland**:ad OR **icelandic***:ad OR **islenska***:ad OR **icelander***:ad OR **islendinga***:ad OR **reykjavik**:ad OR **reykjavikurborg**:ad OR **hofudborgarsvaedid**:ad OR **sudurnes**:ad OR **vesturland**:ad OR **vestfirdir**:ad OR **westfjords**:ad OR **nordurland**:ad OR **austurland**:ad OR **sudurland**:ad OR **kopavogur**:ad OR **hafnarfjordur**:ad OR **switzerland**:ab,ti OR **schweiz**:ab,ti OR **schweizerische**:ab,ti OR **swiss**:ab,ti OR **suisse**:ab,ti OR **aargau**:ab,ti OR **argovia**:ab,ti OR **ausserrhoden**:ab,ti OR **'outer rhodes'**:ab,ti OR **innerrhoden**:ab,ti OR **'inner rhodes'**:ab,ti OR **basel**:ab,ti OR **bern**:ab,ti OR **berne**:ab,ti OR **fribourg**:ab,ti OR **freiburg**:ab,ti OR **geneva**:ab,ti OR **geneve**:ab,ti OR **glarus**:ab,ti OR **graubunden**:ab,ti OR **grisons**:ab,ti OR **grigioni**:ab,ti OR **jura**:ab,ti OR **lucerne**:ab,ti OR **luzern**:ab,ti OR **neuchatel**:ab,ti OR **zurich**:ab,ti OR (**uri**:ab,ti AND (**canton**:ab,ti OR **kanton**:ab,ti)) OR **schwyz**:ab,ti OR **obwalden**:ab,ti OR **nidwalden**:ab,ti OR **zug**:ab,ti OR **solothurn**:ab,ti OR **schaffhausen**:ab,ti OR **thurgau**:ab,ti OR **thurgovia**:ab,ti OR **ticino**:ab,ti OR **tessin**:ab,ti OR **vaud**:ab,ti OR **valais**:ab,ti OR **wallis**:ab,ti OR **'st gallen'**:ab,ti OR **lausanne**:ab,ti OR **winterthur**:ab,ti OR **winterthour**:ab,ti OR **lugano**:ab,ti OR **biel**:ab,ti OR **bienne**:ab,ti OR **switzerland**:ad OR **schweiz**:ad OR **schweizerische**:ad OR **swiss**:ad OR **suisse**:ad OR **aargau**:ad OR **argovia**:ad OR **ausserrhoden**:ad OR **'outer rhodes'**:ad OR **innerrhoden**:ad OR **'inner rhodes'**:ad OR **basel**:ad OR **bern**:ad OR **berne**:ad OR **fribourg**:ad OR **freiburg**:ad OR **geneva**:ad OR **geneve**:ad OR **glarus**:ad OR **graubunden**:ad OR **grisons**:ad OR **grigioni**:ad OR **jura**:ad OR **lucerne**:ad OR **luzern**:ad OR **neuchatel**:ad OR **zurich**:ad OR (**uri**:ad AND (**canton**:ad OR **kanton**:ad)) OR **schwyz**:ad OR **obwalden**:ad OR **nidwalden**:ad OR **zug**:ad OR **solothurn**:ad OR **schaffhausen**:ad OR **thurgau**:ad OR **thurgovia**:ad OR **ticino**:ad OR **tessin**:ad OR **vaud**:ad OR **valais**:ad OR **wallis**:ad OR **'st gallen'**:ad OR **lausanne**:ad OR **winterthur**:ad OR **winterthour**:ad OR **lugano**:ad OR **biel**:ad OR **bienne**:ad OR **norway**:ab,ti OR **norwegian***:ab,ti OR **norge**:ab,ti OR **noreg**:ab,ti OR **norgga**:ab,ti OR **ostfold**:ab,ti OR **akershus**:ab,ti OR **oslo**:ab,ti OR **hedmark**:ab,ti OR **oppland**:ab,ti OR **buskerud**:ab,ti OR **vestfold**:ab,ti OR **telemark**:ab,ti OR **'aust agder'**:ab,ti OR **'vest agder'**:ab,ti OR **rogaland**:ab,ti OR **hordaland**:ab,ti OR **'sogn og fjordane'**:ab,ti OR **'sogn and fjordane'**:ab,ti OR **'sogn fjordane'**:ab,ti OR **'more og romsdal'**:ab,ti OR **'more and romsdal'**:ab,ti OR **'more romsdal'**:ab,ti OR **trondelag**:ab,ti OR **nordland**:ab,ti OR **troms**:ab,ti OR **finnmark**:ab,ti OR **bergen**:ab,ti OR **stavanger**:ab,ti OR **sandnes**:ab,ti OR **trondheim**:ab,ti OR **kristiansand**:ab,ti OR **drammen**:ab,ti OR **fredrikstad**:ab,ti OR **sarpsborg**:ab,ti OR **porsgrunn**:ab,ti OR **skien**:ab,ti OR **tonsberg**:ab,ti OR **alesund**:ab,ti OR **norway**:ad OR **norwegian***:ad OR **norge**:ad OR **noreg**:ad OR **norgga**:ad OR **ostfold**:ad OR **akershus**:ad OR **oslo**:ad OR **hedmark**:ad OR **oppland**:ad OR **buskerud**:ad OR **vestfold**:ad OR **telemark**:ad OR **'aust agder'**:ad OR **'vest agder'**:ad OR **rogaland**:ad OR **hordaland**:ad OR **'sogn og fjordane'**:ad OR **'sogn and fjordane'**:ad OR **'sogn fjordane'**:ad OR **'more og romsdal'**:ad OR **'more and romsdal'**:ad OR **'more romsdal'**:ad OR **trondelag**:ad OR **nordland**:ad OR **troms**:ad OR **finnmark**:ad OR **bergen**:ad OR **stavanger**:ad OR **sandnes**:ad OR **trondheim**:ad OR **kristiansand**:ad OR **drammen**:ad OR **fredrikstad**:ad OR **sarpsborg**:ad OR **porsgrunn**:ad OR **skien**:ad OR **tonsberg**:ad OR **alesund**:ad OR **liechtenstein**:ab,ti OR **vaduz**:ab,ti OR **triesenberg**:ab,ti OR **triesen**:ab,ti OR **schellenberg**:ab,ti OR **schaan**:ab,ti OR **ruggell**:ab,ti OR **planken**:ab,ti OR **mauren**:ab,ti OR **gamprin**:ab,ti OR **eschen**:ab,ti OR **balzers**:ab,ti OR **liechtenstein**:ad OR **vaduz**:ad OR **triesenberg**:ad OR **triesen**:ad OR **schellenberg**:ad OR **schaan**:ad OR **ruggell**:ad OR **planken**:ad OR **mauren**:ad OR **gamprin**:ad OR **eschen**:ad OR **balzers**:ad \|  \| \| #1.2 \| **'turkey (republic)'**/exp OR **turkey**:ab,ti OR **turkiye**:ab,ti OR **turkish**:ab,ti OR **istanbul**:ab,ti OR **marmara**:ab,ti OR **aegean**:ab,ti OR **anatolia**:ab,ti OR **'black sea'**:ab,ti OR **tekirdag**:ab,ti OR **balikesir**:ab,ti OR **izmir**:ab,ti OR **aydin**:ab,ti OR **manisa**:ab,ti OR **bursa**:ab,ti OR **kocaeli**:ab,ti OR **ankara**:ab,ti OR **konya**:ab,ti OR **antalya**:ab,ti OR **adana**:ab,ti OR **hatay**:ab,ti OR **kirikkale**:ab,ti OR **kayseri**:ab,ti OR **zonguldak**:ab,ti OR **kastamonu**:ab,ti OR **samsun**:ab,ti OR **trabzon**:ab,ti OR **erzurum**:ab,ti OR **agri**:ab,ti OR **malatya**:ab,ti OR (**van**:ab,ti AND (**region**:ab,ti OR **subregion**:ab,ti OR **bolgesi**:ab,ti)) OR **gaziantep**:ab,ti OR **sanliurfa**:ab,ti OR **mardin**:ab,ti OR **mersin**:ab,ti OR **turkey**:ad OR **turkiye**:ad OR **turkish**:ad OR **istanbul**:ad OR **marmara**:ad OR **aegean**:ad OR **anatolia**:ad OR **'black sea'**:ad OR **tekirdag**:ad OR **balikesir**:ad OR **izmir**:ad OR **aydin**:ad OR **manisa**:ad OR **bursa**:ad OR **kocaeli**:ad OR **ankara**:ad OR **konya**:ad OR **antalya**:ad OR **adana**:ad OR **hatay**:ad OR **kirikkale**:ad OR **kayseri**:ad OR **zonguldak**:ad OR **kastamonu**:ad OR **samsun**:ad OR **trabzon**:ad OR **erzurum**:ad OR **agri**:ad OR **malatya**:ad OR (**van**:ad AND (**region**:ad OR **subregion**:ad OR **bolgesi**:ad)) OR **gaziantep**:ad OR **sanliurfa**:ad OR **mardin**:ad OR **mersin**:ad \| **292777** \| \| #1.1 \| **'europe'**/exp OR **'european union'**/exp OR **europa**:ab,ti OR **europe***:ab,ti OR **scandinavia***:ab,ti OR **scandinavia***:ad OR **mediterranean**:ab,ti OR **'eea countries'**:ab,ti OR **mediterranean**:ad OR **europe***:ad OR **baltic**:ab,ti OR **baltic**:ad OR **yugoslavia**:ab,ti OR **jugoslavija**:ab,ti OR **jugoslavija**:ad OR **yugoslavia**:ad OR **'eu country'**:ab,ti OR **'eu countries'**:ab,ti OR **global***:ab,ti OR **world**:ab,ti OR **worldwide**:ab,ti \| **2166** \| | |

## Cochrane Library (CDSR, DARE, HTA, EED)

Date of the search: 12/03/2015

Language limits: no limits

Date limits: 2005-2015

Number of results:

- CDSR: 3
- DARE: 7
- HTA: 2
- EED: 25

**ID Search Hits**

#1 MeSH descriptor: [Prevalence] explode all trees 3943

#2 MeSH descriptor: [Population Surveillance] explode all trees 585

#3 MeSH descriptor: [Seroepidemiologic Studies] explode all trees 134

#4 seroprevalence* or seroepidemiolog* or "sero epidemiologic" or "sero epidemiological" or "sero epidemiology" or serosurvey* or serolog* or epidemiolog* or surveillance:ti,ab,kw 15921

#5 #1 or #2 or #3 or #4 19532

#6 MeSH descriptor: [Hepatitis B] explode all trees 1939

#7 MeSH descriptor: [Hepatitis B Antibodies] explode all trees 555

#8 MeSH descriptor: [Hepatitis B Antigens] explode all trees 940

#9 MeSH descriptor: [Hepatitis B virus] explode all trees 671

#10 MeSH descriptor: [Hepatitis C] explode all trees 2287

#11 MeSH descriptor: [Hepatitis C Antigens] explode all trees 15

#12 MeSH descriptor: [Hepatitis C Antibodies] explode all trees 112

#13 MeSH descriptor: [Hepacivirus] explode all trees 1001

#14 "hepatitis b" or "hepatitis c" or hepaciviru* or "hbv" or "hcv" or "hbsag" or "hbs ag" or "Australia Antigen" or "Australia Antigens":ti,ab,kw Publication 6994

#15 #6 or #7 or #8 or #9 or #10 or #11 or #12 or #13 or #14 8380

#16 #15 and #5 517

**Supplementary Table S2.** Study inclusion and exclusion criteria

| **Inclusion criteria** | **Exclusion criteria** |
| --- | --- |
| Articles published in 2005 or later reporting data from population sampled from the year 2000 onwards, including studies with data collection ending after 2000 (irrespective of start date) | Articles falling outside the specified sampling period or publication date range |
| Articles reporting data from one or more EU/EEA MS and/or any of their regions/districts | Articles reporting data on non EU/EEA countries only |
| Articles reporting HBsAg/anti-HCV (and DNA/RNA) prevalence rates in humans | Articles not reporting data on HBsAg/anti-HCV prevalence or DNA/RNA or if virological markers tested for were not specified  Article reporting only self-reported (i.e. unconfirmed) HBsAg/anti-HCV prevalence |
| Articles reporting data from the general population and/or key subgroups | Articles reporting data on specific high-risk groups only i.e. MSM, PWID |
|  | Articles reporting modelled data only |
|  | Articles reporting only data from a study not conducted in humans, environmental studies, technology assessments (studies on diagnostic and/or laboratory methods) |
|  | Opinion papers, editorials, guidelines or recommendations , perspectives and correspondence articles, systematic reviews or meta-analysis |
|  | Articles reporting data on the general population or pregnant women in countries with a population of >5 million inhabitants, but has a sample size <100 participants or has a sample size <50 participants for countries with a population of <5 million |

**Supplementary Table S3.** List and definition of key subgroups included in the systematic review

| **Population category** | **Definition** |
| --- | --- |
| **General population** | People living in a defined geographical area (all ages or adults only), excluding specific low risk populations such as children |
|  | Patients attending community and primary care settings, excluding hospitalized patients |
|  | Workforce or specific professional groups (e.g. workplace screening) excluding health care workers and specific recreational/ sports-related population sub-groups |
| **Pregnant women** | Pregnant women undergoing antenatal care screening |
| **Blood donors** | First-time blood donors (pre-screened and non-pre-screened) |

**Supplementary Table S4.** Variables used in Data Extraction

| **Variable** | **Description** | **Values** |
| --- | --- | --- |
| PMID | PubMed identifier, accession number | Numerical |
| Study ID | The ID links each study to the source article | Alpha-numerical (PMID plus a, b, c…. for each study) |
| First author | Surname of first author of the article |  |
| Year of publication | Year of publication of the article | Year: yyyy |
| Type of publication | Type of publication | Original article; review; report; abstract; commentary; erratum |
| Exclusion | If required, reason for exclusion |  |
| Disease | Disease for which the study report prevalence estimates | HBV; HCV; both |
| Country | Country for which the study report prevalence estimates | Country name |
| Period of sampling | Year/s during which study sampling was conducted | Year: yyyy |
| Population coverage | Coverage of the population by the sampling design in geographic/demographic terms | National; regional; multicentre; single centre/local |
| Population | Target population sampled in the study | General population; pregnant women; MSM; prisoner |
| Study population details | Narrative field for any further relevant information on the study population |  |
| Sampling approach | Description of sampling approach | Random sampling; convenience sampling; respondent-driven (i.e. snowballing); exhaustive (screening); not specified; other |
| Sample size | Total sample size (for all estimates) | Numerical |
| Response rate | Percentage of respondents | % |
| Type of sample | Description of sample type | Serum, saliva; dry blood spot |
| Type of test | Description of laboratory test used | HBsAg; HBV DNA; HBV rapid test; Anti-HCV; anti-HCV immunoblot; HCV RNA; HCV rapid test |
| Age group | Description of age group | 0-17 (children); >17 (adults); all |
| Mean / median age | Mean or median age of study population | Numerical |
| Age range | Minimum and maximum age of study population | Numerical |
| Prevalence number per estimate | Number screened and number positive (HBsAg, HBV DNA, anti-HCV, HCV RNA) | Numerical |
| Prevalence % per estimate | Prevalence estimate (HBsAg, HBV DNA, anti-HCV, HCV) | % |
| 95%CI per estimate | Calculated using Fisher method for 95% CI | % |
| Standardized | Weighted/standardized prevalence estimate available | Yes/No |
| Comments |  |  |

##### **Supplementary Table S5.** Risk of bias framework for general population studies

| **Domain** | **Scores and description** | |
| --- | --- | --- |
| Age | 0 | 1 |
|  | Clear age bias (towards children for example), not representative for the general population; no information | No clear bias in age profile of respondents; representative for the general population age distribution |
| Gender | 0 | 1 |
|  | Clear bias in gender; no information to suggest representativeness for the general population | No clear bias in gender distribution of subjects; could be considered representative if information is limited |
| Sampling method | 0 - Non-random or non-exhaustive  1 - Exhaustive or random and <50/60% response rate or no info  2 - Exhaustive or random and >50/60% response rate | |
| Geographical coverage | 0 – Single centre/local; 1 – Multi-centre/local or regional; 2 – Multi-centre/national | |

**Supplementary Table S6**. Risk of bias framework for studies among pregnant women

| **Domain** | **Scores and description** | |
| --- | --- | --- |
| Sampling method | 0 | 1 |
|  | Non-random or non-exhaustive | Exhaustive or random |
| Geographical coverage | 0 – Single centre/local; 1 – Multi-centre/local or regional; 2 – Multi-centre/national | |

**Supplementary Table S7.** Extracted and pooled (where possible) HBsAg prevalence estimates for the general population from included studies

| **Country** | **Author (Publ.Year)** | **Geographical coverage** | **Sampling period** | **Sampling method** | **Study population details** | **Age range** | **Sample size** | **Prevalence of HBsAg (95%CI)** |
| --- | --- | --- | --- | --- | --- | --- | --- | --- |
| **Belgium*** | Quoilin (2007) ^(^[^1^](#_ENREF_23)^)^ | Regional | 2002 | Random | Region of Flanders | Standardized | Standardized | 0∙7% (0∙5- 0∙8) |
| Belgium | Quoilin (2007) ^(^[^1^](#_ENREF_23)^)^ | Regional | 2002 | Random | Region of Flanders | 0 to >65 | 1,830 | 0∙7% (0∙4 - 1∙2) |
| Belgium | Nardone (2009) ^(^[^2^](#_ENREF_24)^)^ | N/R | 2002-2003 | Convenience | Geographically and gender representative residual lab samples | 1 to 15 | 1,175 | 0∙7% (0∙3 - 1∙3) |
| Belgium | Nardone (2009) ^(^[^2^](#_ENREF_24)^)^ | N/R | 2002-2003 | Convenience | Geographically and gender representative residual lab samples | 16 to 39 | 321 | 0∙6% (0∙1 - 2∙2) |
| **Croatia*** | Vilibic-Cavlek (2014) ^(^[^3^](#_ENREF_25)^)^ | Multi-centre | 2010-2011 | Convenience | Multi-centre study of patients attending a medical check-up, covers 20% of regions | 20 to 80 | 2,009 | 0∙7% (0∙4 - 1∙2) |
| Croatia | Burek (2010) ^(4)^ | Multi-centre | 2005-2007 | N/R | Multi-centre study of staff in 20 prisons (10∙9% of all prison staff) | N/R | 259 | 2∙3% (0∙9 - 5∙0) |
| **Czech Republic*** | Nardone (2009) ^(^[^2^](#_ENREF_24)^)^ | N/R | 2001 | Random |  | Standardized | Standardized | 0∙6% |
| Czech Republic | Nardone (2009) ^(^[^2^](#_ENREF_24)^)^ | N/R | 2001 | Random |  | 16 to >40 years | 1,669 | 0∙4% (0∙1-0∙8) |
| Czech Republic | Nardone (2009) ^(^[^2^](#_ENREF_24)^)^ | N/R | 2001 | Random |  | 1 to 15 years | 975 | 0∙3% (0∙1-0∙9) |
| Czech Republic | Nardone (2009) ^(^[^2^](#_ENREF_24)^)^ | N/R | 2001 | Random |  | 16 to 39 years | 1,148 | 0∙2% (0∙0-0∙6) |
| Czech Republic | Nardone (2009) ^(^[^2^](#_ENREF_24)^)^ | N/R | 2001 | Random |  | >40 years | 521 | 0∙8% (0∙2-2∙0) |
| **France*** | Meffre (2010) ^(^[^5^](#_ENREF_27)^)^ | National | 2004 | Random | National sample obtained via primary health care insurance units | 18 to 80 | 18,230 | 0∙7% (0∙5 - 0∙9) |
| France | Bottero (2014) ^(6)^ | Multi-centre | 2010-2011 | Convenience | Multi-centre screening study in Paris region includes multiple risk groups such as migrants and PWID | Mean age 33 | 3,929 | 2∙2% (1∙7 - 2∙7) |
| Germany# | Poethko-Müller (2013) ^(^[^7^](#_ENREF_29)^)^ | National | 2008 - 2011 | Random | National coverage, DEGS1 study | 18 to 79 | 7,047 | 0∙3% (0∙2 - 0∙6) |
| Germany# | Huetter (2014) ^(^[^8^](#_ENREF_30)^)^ | Local | 2002 | Random | Residents of Leutkirch, South Germany | 18 to 65, mean age 39∙4 | 2,256 | 0∙7% (0∙4 - 1∙1) |
| Germany | Wolffram (2015) ^(9)^ | Regional | 2012-2013 | Convenience | Check-up 35+ attendees (mid-life health check) in Northern Rhine Westphalia | Mean age 57∙5 | 21,008 | 0∙5% (0∙4 - 0∙6) |
| **Germany*** | Pooled | Pooled | 2002; 2008-2011 | Pooled | Pooled | Pooled | 9,303 | 0∙4% (0∙3 – 0∙5) |
| **Greece*** | Drositis (2013) ^(^[^10^](#_ENREF_32)^)^ | Regional | 2006-2010 | Random | Adult residents of Arkalochori, a province in Crete | Mean age 52 | 876 | 3∙3% (2∙2 - 4∙7) |
| Greece | Dounias (2005) ^(^[^11^](#_ENREF_33)^)^ | Local | 1999-2001 | Convenience | Municipal solid waste workers | Mean age 42 | 159 | 7∙5% (4∙0 - 12∙8) |
| **Hungary*** | Treso (2012) ^(^[^12^](#_ENREF_34)^)^ | National | 2007-2009 | Convenience | Prison staff | 21 to 60 | 1,066 | 0∙4% (0∙1 - 1∙0) |
| **Ireland*** | Talento (2010) ^(13)^ | National | 1992-2009 | Exhaustive | Living and deceased solid organ donors | N/R | 1,478 | 0∙1% (0∙0 - 0∙4) |
| Ireland | Nardone (2009) ^(^[^2^](#_ENREF_24)^)^ | N/R | 2003 | Convenience | Geographically and gender representative residual lab samples | 1 to 15 | 877 | 0∙0% (0∙0 - 0∙9) |
| Ireland | Nardone (2009) ^(^[^2^](#_ENREF_24)^)^ | N/R | 2003 | Convenience | Geographically and gender representative residual lab samples | 16 to >40 | 1,658 | 0∙1% (0∙0 - 0∙4) |
| Ireland | Nardone (2009) ^(^[^2^](#_ENREF_24)^)^ | N/R | 2003 | Convenience | Geographically and gender representative residual lab samples | 16 to 39 | 1,194 | 0∙1% (0∙0 - 0∙5) |
| Ireland | Nardone (2009) ^(^[^2^](#_ENREF_24)^)^ | N/R | 2003 | Convenience | Geographically and gender representative residual lab samples | >40 | 464 | 0∙2% (0∙0 - 1∙2) |
| Italy# | Cozzolongo (2009) ^(^[^14^](#_ENREF_36)^)^ | Local | 2005-2007 | Random | GP records in Bari, Apuglia | 18 to 93, mean age 47 | 2,195 | 0∙5% (0∙3 - 1∙0) |
| Italy# | Pendino (2005) ^(^[^15^](#_ENREF_37)^)^ | Local | 2002-2003 | Random | Census data from Cittanova, Calabria | 12 to 95 | 1,645 | 0∙8% (0∙4 - 1∙3) |
| Italy# | Floreani (2006) ^(^[^16^](#_ENREF_38)^)^ | Local | 2001 | Random | Residents of Arsita (Central Italy) | >8 | 142 | 0∙7% (0∙0 - 3∙9) |
| Italy | Boccalini (2013) ^(^[^17^](#_ENREF_39)^)^ | Regional | 2009 | Convenience | Residual blood samples from 0∙05% of residents in Tuscany | 1 to 50 | 1,071 | 2∙0% (1∙2 – 3∙0) |
| Italy | Fabris (2008) ^(^[^18^](#_ENREF_40)^)^ | Local | 2002 | Exhaustive | Vicenza, North East Italy. A broken sewer pipe prompted HAV vaccination, subjects were also tested for HBV/HCV | Mean age 42∙1 | 965 | 1∙0% (0∙5 - 1∙9) |
| Italy | Del Corno (2006) ^(^[^1^](#_ENREF_41)^9)^ | Local | N/R | Random | Four towns in Isola Bergamasca (Northern Italy) | 20 to 70 | 2,829 | 5∙8% (5∙0 - 6∙7) |
| Italy | Dazzani (2009) ^(^[^2^](#_ENREF_42)^0)^ | Local | 2008 | Convenience | Survey of residents in Bagnacavallo (Emilia-Romagna) | 30 to 60 | 3,207 | 0∙6% (0∙4 - 1∙0) |
| Italy | De Paschale (2012) ^(21)^ | Local | 2007-2008 | Convenience | Hospital lab samples from Legnano Hospital in Northern Italy | Mean age 51∙4 | 22,758 | 2∙1% (2∙0 - 2∙3) |
| Italy | Da Villa (2007) ^(22)^ | Local | 2006 | N/R | Cohort in Afragola, Naples | 6 to 58 | 1,540 | 0∙9% (0∙5 - 1∙5) |
| Italy | Squeri (2006) ^(23)^ | Local | 2005 | Convenience | Municipal solid waste workers | 20 to 68 | 327 | 4∙0% (2∙1 - 6∙7) |
| **Italy*** | Pooled | Pooled | 2001; 2002-2003; 2005-2007 | Pooled | Pooled | Pooled | 3,982 | 0∙7% (0∙4 – 1∙0) |
| **The Netherlands*** | Hahné (2012) ^(^[^24^](#_ENREF_18)^)^ | National | 2006-2007 | Random | National study (Pienter 2) | >18 months | 6,246 | 0∙2% (0∙1 - 0∙4) |
| The Netherlands | Veldhuijzen (2009) ^(2^[^5^](#_ENREF_53)^)^ | Local | 2004 | Random | Rotterdam municipal population register | 18 to 65 | 284 | 0∙7% (0∙1 - 2∙5) |
| Poland | Hartleb (2012) ^(^[^26^](#_ENREF_46)^)^ | National | N/R | Exhaustive | Study among elderly population∙ Sample from national population register, 42∙6% response | >65, mean age 79∙4 | 3,826 | 1∙1% (0∙8 - 1∙5) |
| Poland | Pszenny (2012) ^(^[^27^](#_ENREF_47)^)^ | Regional | 2000-2008 | Convenience | Retrospective study among deceased potential blood donors, 75% male | N/R | 4,774 | 0∙9% (0∙7 - 1∙2) |
| **Romania*** | Gheorghe (2013) ^(^[^28^](#_ENREF_48)^)^ | National | 2006-2008 | Random | National cross-sectional population survey | 18 to 69 | 13,127 | 4∙4% (4∙0 - 4∙8) |
| Romania | Nardone (2009) ^(^[^2^](#_ENREF_24)^)^ | N/R | 2002 | Convenience | Geographically and gender representative residual lab samples | 1 to 15 | 630 | 5∙1% (3∙5 - 7∙1) |
| Romania | Nardone (2009) ^(^[^2^](#_ENREF_24)^)^ | N/R | 2002 | Convenience | Geographically and gender representative residual lab samples | 16 to >40 | 629 | 6∙2% (4∙4 - 8∙4) |
| Romania | Nardone (2009) ^(^[^2^](#_ENREF_24)^)^ | N/R | 2002 | Convenience | Geographically and gender representative residual lab samples | 16 to 39 | 276 | 7∙6% (4∙8 - 11∙4) |
| Romania | Nardone (2009) ^(^[^2^](#_ENREF_24)^)^ | N/R | 2002 | Convenience | Geographically and gender representative residual lab samples | >40 | 353 | 5∙1% (3∙0 - 7∙9) |
| Slovakia | Nardone (2009) ^(^[^2^](#_ENREF_24)^)^ | National | 2002 | Random |  | 1 to 15 | 1,623 | 0∙1% (0∙0 - 0∙4) |
| **Slovakia*** | Nardone (2009) ^(^[^2^](#_ENREF_24)^)^ | National | 2002 | Random |  | 16 to >40 | 1,946 | 1∙1% (0∙7 - 1∙6) |
| Slovakia | Nardone (2009) ^(^[^2^](#_ENREF_24)^)^ | National | 2002 | Random |  | 16 to 39 | 1,270 | 0∙9% (0∙4 - 1∙5) |
| Slovakia | Nardone (2009) ^(^[^2^](#_ENREF_24)^)^ | National | 2002 | Random |  | >40 | 676 | 1∙5% (0∙7 - 2∙7) |
| Spain# | Pedraza-Flechas (2014) ^(^[^29^](#_ENREF_49)^)^ | Regional | 2008-2009 | Random | Attendees of blood extraction centres in region of Madrid∙ Bi-stage, cluster stratified sampling | 16 to 80 | 3,695 | 0∙7% (0∙5 - 1∙0) |
| Spain# | Lopez-Izquierdo (2007) ^(^[^30^](#_ENREF_50)^)^ | Regional | 2003 | Random | Study in Valladolid∙ Stratified random sampling based on health card | N/R | 364 | 0∙0% (0∙0 - 1∙0) |
| Spain# | Salleras (2007) ^(^[^31^](#_ENREF_51)^)^ | Regional | 2002 | Random | Two stage cluster sample from municipal electoral registers in Catalonia | 15 to >65 | 1,296 | 1∙4% (0∙8 -2∙2) |
| Spain | Salleras (2007) ^(^[^31^](#_ENREF_51)^)^ | Regional | 2002 | Random | Two stage cluster sample from schools and municipal electoral registers in Catalonia | 5 to >65 | 2,620 | 0∙7% (0∙4 - 1∙1) |
| Spain | Salleras (2007) ^(^[^31^](#_ENREF_51)^)^ | Regional | 2002 | Random | Two stage cluster sample from schools in Catalonia | 5 to 14 | 1,324 | 0∙0% (0∙0 - 0∙3) |
| Spain | Calleja-Panero (2013) ^(^[^32^](#_ENREF_52)^)^ | Multi-centre | 2007-2010 | Convenience | Study in Murcia and Madrid of working population at yearly insurance check-up∙ 73% male∙ | 42 | 5,017 | 0∙7% (0∙5 - 1∙0) |
| **Spain*** | Pooled | Pooled | 2002; 2003; 2008-2009 | Pooled | Pooled | Pooled | 5,355 | 0∙8% (0∙6 - 1∙1) |
| United Kingdom | Pepas (2011) ^(^[^33^](#_ENREF_54)^)^ | Local | 2007-2009 | Exhaustive | Patients undergoing Assisted Reproductive Treatment at Guy & St Thomas Hospital, London | N/R | 3,910 | 1∙7% (1∙3 - 2∙2) |

# Estimates that were used for the pooled estimate

* Higher quality estimate

***References***

1. Quoilin S, Hutse V, Vandenberghe H, et al. A population-based prevalence study of hepatitis A, B and C virus using oral fluid in Flanders, Belgium. Eur J Epidemiol. 2007;22(3):195-202

2. Nardone A, Anastassopoulou CG, Theeten H, et al. A comparison of hepatitis B seroepidemiology in ten European countries. Epidemiol Infect. 2009 Jul;137(7):961-9.

3. Vilibic-Cavlek T, Kucinar J, Ljubin-Sternak S, Kaic B, Lazaric-Stefanovic L, Kolaric B. Prevalence of viral hepatitis in Croatian adult population undergoing routine check-up, 2010–2011. Cent Eur J Public Health. 2014 Mar;22(1):29-33

4. Burek V, Horvat J, Butorac K, Mikulic R. Viral hepatitis B, C and HIV infection in Croatian prisons. Epidemiol Infect. 2010;138(11):1610–20.

5. Meffre C, Le Strat Y, Delarocque-Astagneau E, et al. Prevalence of hepatitis B and hepatitis C virus infections in France in 2004: social factors are important predictors after adjusting for known risk factors. J Med Virol. 2010 Apr;82(4):546-55.

6. Bottero J, Boyd A, Lemoine M, et al. Current state of and needs for hepatitis B screening: results of a large screening study in a low-prevalent, metropolitan region. PloS one. 2014;9(3):e92266.

7. Poethko-Müller C, Zimmermann R, Hamouda O, et al. Die Seroepidemiologie der Hepatitis A, B und C in Deutschland. Ergebnisse der Studie zur Gesundheit Erwachsener in Deutschland (DEGS1). Bundesgesundheitsblatt Gesundheitsforschung Gesundheitsschutz. 2013;56:707–15.

8. Huetter ML, Fuchs M, Hanle MM, et al. Prevalence of risk factors for liver disease in a random population sample in southern Germany. Zeitschrift fur Gastroenterologie. 2014;52(6):558-63.

9. Wolffram I, Petroff D, Batz O, et al. Prevalence of elevated ALT values, HBsAg, and anti-HCV in the primary care setting and evaluation of guideline defined hepatitis risk scenarios. J Hepatol. 2015 Jun;62(6):1256-64

10. Drositis I, Bertsias A, Lionis C, Kouroumalis E. Epidemiology and molecular analysis of hepatitis A, B and C in a semi-urban and rural area of Crete. Eur J Intern Med. 2013 Dec;24(8):839-45.

11. Dounias G, Kypraiou E, Rachiotis G, Tsovili E, Kostopoulos S. Prevalence of hepatitis B virus markers in municipal solid waste workers in Keratsini (Greece). Occup Med (Lond). 2005 Jan;55(1):60-3.

12. Treso B, Barcsay E, Tarjan A, et al. Prevalence and correlates of HCV, HVB, and HIV infection among prison inmates and staff, Hungary. J Urban Health. 2012 Feb;89(1):108-16

13. Talento AF, Hickey D, O'Neill D, et al. Serological screening of solid organ transplant donors in Ireland. J Hosp Infect. 2010;76:S58-S9.

14. Cozzolongo R, Osella AR, Elba S, et al. Epidemiology of HCV infection in the general population: a survey in a southern Italian town. Am J Gastroenterol. 2009 Nov;104(11):2740-6

15. Pendino GM, Mariano A, Surace P, et al. Prevalence and etiology of altered liver tests: a population-based survey in a Mediterranean town. Hepatology. 2005 May;41(5):1151-9.

16. Floreani A, Betterle C, Carderi I, et al. Is hepatitis C virus a risk factor for thyroid autoimmunity? J Viral Hepat. 2006 Apr;13(4):272-7.

17. Boccalini S, Pellegrino E, Tiscione E, et al. Sero-epidemiology of hepatitis B markers in the population of Tuscany, Central Italy, 20 years after the implementation of universal vaccination. Hum Vaccin Immunother. 2013 Mar;9(3):636-41.

18. Fabris P, Baldo V, Baldovin T, et al. Changing epidemiology of HCV and HBV infections in Northern Italy: a survey in the general population. J Clin Gastroenterol. 2008 May-Jun;42(5):527-32.

19. Del Corno G, Civardi E. Intrafamilial transmission of hepatitis B and C viruses in an Italian local health district. Ann Ig. 2006 Jul-Aug;18(4):287-95.

20. Dazzani F, Giacomoni P, Foschi FG, et al. Prevalence of transaminase abnormalities in general population in XXI century: Preliminary results from the Bagnacavallo study. Hepatology. 2009;50:794A-5A.

21. De Paschale M, Manco MT, Belvisi L, et al. Epidemiology of hepatitis D virus (HDV) infection in an urban area of northern Italy. Infection. 2012;40(5):485-91.

22. Da Villa G, Romano L, Sepe A, et al. Impact of hepatitis B vaccination in a highly endemic area of south Italy and long-term duration of anti-HBs antibody in two cohorts of vaccinated individuals. Vaccine. 2007;25(16):3133–6.

23. Squeri R, La Fauci V, Sindoni L, Cannavo G, Ventura Spagnolo E. Study on hepatitis B and C serologic status among municipal solid waste workers in Messina (Italy). J Prev Med Hyg. 2006 Sep;47(3):110-3.

24. Hahné SJ, De Melker HE, Kretzschmar M, et al. Prevalence of hepatitis B virus infection in Netherlands in 1996 and 2007. Epidemiol Infect. 2012 Aug;140(8):1469-80.

25. Veldhuijzen IK, van Driel HF, Vos D, et al. Viral hepatitis in a multi-ethnic neighborhood in the Netherlands: results of a community-based study in a low prevalence country. Int J Infect Dis. 2009 Jan;13(1):e9-e13.

26. Hartleb M, Gutkowski K, Zejda JE, Chudek J, Wiecek A. Serological prevalence of hepatitis B virus and hepatitis C virus infection in the elderly population: Polish nationwide survey–PolSenior. Eur J Gastroenterol Hepatol. 2012 Nov;24(11):1288-95.

27. Pszenny A, Hreńczuk M, Czerwiński J, Danielewicz R, Małkowski P. Epidemiologia zakażeń wirusami zapalenia wątroby typu B i C wśród zmarłych dawców narządów w Polsce. Probl Hig Epidemiol. 2012;93(3):579-85.

28. Gheorghe L, Csiki IE, Iacob S, Gheorghe C. The prevalence and risk factors of hepatitis B virus infection in an adult population in Romania: a nationwide survey. Eur J Gastroenterol Hepatol. 2013;25(1):56-64.

29. Pedraza-Flechas AM, Garcia-Comas L, Ordobas-Gavin M, et al. Hepatitis B virus infection and vaccine-induced immunity in Madrid (Spain). Gaceta sanitaria/SESPAS. 2014;28(6):492–5.

30. Lopez-Izquierdo R, Udaondo MA, Zarzosa P, et al. [Seroprevalence of viral hepatitis in a representative general population of an urban public health area in Castilla y Leon (Spain)]. Enferm Infecc Microbiol Clin. 2007;25(5):317-23.

31. Salleras L, Dominguez A, Bruguera M, et al. Declining prevalence of hepatitis B virus infection in Catalonia (Spain) 12 years after the introduction of universal vaccination. Vaccine. 2007;25(52):8726-31.

32. Calleja-Panero JL, Llop-Herrera E, Ruiz-Moraga M, et al. Prevalence of viral hepatitis (B and C) serological markers in healthy working population. Rev Esp Enferm Dig. 2013 May-Jun;105(5):249-54.

33. Pepas L, Macmahon E, El Toukhy T, Khalaf Y, Braude P. Viral screening before each cycle of assisted conception treatment is expensive and unnecessary: a survey of results from a UK inner city clinic. Hum Fertil (Camb). 2011 Dec;14(4):224-9.

**Supplementary Table S8.** Extracted and pooled (where possible) anti-HCV prevalence estimates for the general population from included studies

| **Country** | **Author (Publ.Year)** | **Geographical coverage** | **Sampling period** | **Sampling**  **method** | **Study population details** | **Age range** | **Sample size** | **Prevalence of**  **anti-HCV (95% CI)** |
| --- | --- | --- | --- | --- | --- | --- | --- | --- |
| **Belgium*** | Quoilin (2007) ^(^[^1^](#_ENREF_23)^)^ | Regional | 2002 | Random | Region of Flanders | 0 to >65 | 1,830 | 0∙1% (0∙0 - 0∙4) |
| Croatia | Burek (2010) ^(^[^2^](#_ENREF_26)^)^ | Multi-centre | 2005-2007 | N/R | Multi-centre study of staff in 20 prisons (10∙9% of all prison staff) | N/R | 259 | 0∙0% (0∙0 - 1∙4) |
| **Croatia*** | Vilibic-Cavlek (2014) ^(^[^3^](#_ENREF_25)^)^ | Multi-centre | 2010-2011 | Convenience | Multi-centre study of patients attending a medical check-up, covers 20% of regions | 20 to 80 | 1,930 | 0∙9% (0∙6 - 1∙5) |
| **France*** | Meffre (2010) ^(^[^4^](#_ENREF_27)^)^ | National | 2004 | Random | National sample obtained via primary health care insurance units | 18 to 80 | 18,230 | 0∙8% (0∙7 - 1∙1) |
| France | Sahajian (2007) ^(^[^5^](#_ENREF_55)^)^ | Multi-centre | 2003-2004 | Mixed | Under-privileged populations sampled via primary care services∙ Includes a small number of homeless people (n=89) and former/active PWID (n=16) | 18 to >60 | 944 | 4∙7% (3∙4 - 6∙2) |
| France | Poynard (2009) ^(^[^6^](#_ENREF_56)^)^ | Multi-centre | N/R | Convenience | Attendees of social security health centres | >40 | 7,463 | 0∙9% (0∙7 - 1∙1) |
| Germany# | Poethko-Müller (2013) ^(^[^7^](#_ENREF_29)^)^ | National | 2008-2011 | Random | National coverage: DEGS1 study | 18 to 79 | 7,047 | 0∙3% (0∙1 - 0∙5) |
| Germany# | Huetter (2014) ^(^[^8^](#_ENREF_30)^)^ | Local | 2002 | Random | Residents of Leutkirch, South Germany | 18 to 65, mean age 39∙4 | 2,256 | 0∙6% (0∙3 – 1∙0) |
| Germany | Wolffram (2015) ^(9)^ | Regional | 2012-2013 | Convenience | Check-up 35+ attendees (mid-life health check) in Northern Rhine Westphalia | Mean age 57∙5 | 21,008 | 1∙0% (0∙8 – 1∙1) |
| **Germany*** | Pooled | Pooled | 2002; 2008-2011 | Pooled | Pooled | Pooled | 9,303 | 0∙4% (0∙3 – 0∙5) |
| **Greece*** | Drositis (2013) ^(10)^ | Regional | 2006-2010 | Random | Adult residents of Arkalochori, a province in Crete | Mean age 52 | 876 | 2∙2% (1∙3 - 3∙4) |
| **Hungary*** | Treso (2012) ^(^[^11^](#_ENREF_34)^)^ | National | 2007-2009 | Convenience | Prison staff | 21 to 60 | 1,066 | 0∙5% (0∙2 - 1∙1) |
| **Ireland*** | Talento (2010) ^(^[^12^](#_ENREF_35)^)^ | National | 1992-2009 | Exhaustive | Living and deceased solid organ donors | N/R | 1,478 | 0∙1% (0∙0 - 0∙4) |
| Italy# | Cozzolongo (2009) ^(1^[^3^](#_ENREF_36)^)^ | Local | 2005-2007 | Random | GP records in Bari, Apuglia | 18 to 93, mean age 47 | 2,195 | 2∙6% (2∙0 - 3∙4) |
| Italy# | Pendino (2005) ^(^[^14^](#_ENREF_37)^)^ | Local | 2002-2003 | Random | Census data from Cittanova, Calabria | 12 to 95 | 1,645 | 6∙5% (5∙4 - 7∙8) |
| Italy# | Floreani (2006) ^(^[^15^](#_ENREF_38)^)^ | Local | 2001 | Random | Residents of Arsita (Central Italy) | >8 | 697 | 10∙2% (8∙0 - 12∙7) |
| Italy# | Petti (2006) ^(^[^16^](#_ENREF_57)^)^ | Local | N/R | Random | GP registration∙ Local coverage | >25 | 289 | 16∙3% (12∙2 - 21∙0) |
| Italy | Petti (2006) ^(^[^16^](#_ENREF_57)^)^ | Local | N/R | Random | GP registration∙ Local coverage | 25-49 | 133 | 3∙0% (0∙8 - 7∙5) |
| Italy | Petti (2006) ^(^[^16^](#_ENREF_57)^)^ | Local | N/R | Random | GP registration∙ Local coverage | >50 | 156 | 27∙6% (20∙7 - 35∙3) |
| Italy | Parisi (2014) ^(^[^17^](#_ENREF_58)^)^ | Local | 2011-2014 | Convenience | Milan∙ Hospital (2 points in one location), STI clinics (n=1) and GPs (n=6) | >18 | 4,507 | 0∙6% (0∙4 - 0∙9) |
| Italy | Guadagnino (2013) ^(^[^18^](#_ENREF_59)^)^ | Local | 2010 | Random | Study in a small town in Calabria | >18 | 1,012 | 5∙7% (4∙4 - 7∙4) |
| Italy | Fabris (2008) ^(^[^20^](#_ENREF_40)^)^ | Local | 2002 | Exhaustive | Vicenza, North East Italy. A broken sewer pipe prompted HAV vaccination, subjects were also tested for HBV/HCV | Mean age 42∙1 | 965 | 2∙6% (1∙7 - 3∙8) |
| Italy | Montella (2005) ^(21)^ | Regional | 2000-2002 | Convenience | Naples, residual sera from primary care tests | 19 to 65 | 1,972 | 8∙2% (7∙0 - 9∙5) |
| Italy | Del Corno (2006) ^(22)^ | Local | N/R | Random | Four towns in Isola Bergamasca (Northern Italy) | 20 to 70 | 960 | 4∙7% (3∙4 - 6∙2) |
| Italy | Dazzani (2009) ^(^[^2^](#_ENREF_42)^3)^ | Local | 2008 | Convenience | Survey of residents in Bagnacavallo (Emilia-Romagna) | 30 to 60 | 3,207 | 1∙1% (0∙8 - 1∙5) |
| Italy | De Paschale (2012) ^(2^[^4^](#_ENREF_43)^)^ | Local | 2007-2008 | Convenience | Hospital lab samples from Legnano Hospital in Northern Italy | Mean age 51∙4 | 425 | 4∙7% (2∙9 - 7∙2) |
| Italy | Squeri (2006) ^(^[^25^](#_ENREF_45)^)^ | Local | 2005 | Convenience | Municipal solid waste workers | 20 to 68 | 327 | 0∙9% (0∙2 - 2∙7) |
| **Italy*** | Pooled | Pooled | N/R | Pooled | Pooled | Pooled | 4,826 | 5∙9% (5∙2 – 6∙6) |
| **Latvia*** | Tolmane (2011) ^(2^[^6^](#_ENREF_61)^)^ | National | 2008 | Random | GP registration | 18 to 94 | 1,459 | 2∙4% (1∙7 - 3∙3) |
| Lithuania | Liakina (2012) ^(^[^2^](#_ENREF_62)^7)^ | National (covering 75% of population) | 2010 | Convenience | Shopping centre attendees  (62% female) | N/R | 1,514 | 2∙4% (1∙7 - 3∙4) |
| Lithuania | Liakina (2012) ^(^[^2^](#_ENREF_62)^7)^ | National (covering 75% of population) | 2010 | Convenience | Shopping centre attendees  (62% female) | Mean across age ranges |  | 2∙8% |
| Lithuania | Liakina (2012) ^(^[^2^](#_ENREF_62)^7)^ | National (covering 75% of population) | 2010 | Convenience | Shopping centre attendees  (62% female) | Standardized |  | 2∙8% |
| Lithuania | Liakina (2012) ^(^[^2^](#_ENREF_62)^7)^ | National (covering 75% of population) | 2010 | Convenience | Shopping centre attendees  (62% female) | Standardized to European population |  | 2∙9% |
| **The Netherlands*** | Vriend (2013) ^(^[^28^](#_ENREF_66)^)^ | National | 2006-2007 | Random | National study (Pienter 2) | 15 to 79 | 4,046 | 0∙1% (0∙0 - 0∙2) |
| The Netherlands | Veldhuijzen (2009) ^(29)^ | Local | 2004 | Random | Rotterdam municipal population register | 18 to 65 | 284 | 1∙1% (0∙2 - 3∙1) |
| The Netherlands | Slavenburg (2008) ^(30)^ | Regional | 2006 | Convenience | GP attendees in Arnhem and Nijmegen who had blood taken as part of clinical work up | N/R | 2,200 | 0∙2% (0∙1 - 0∙5) |
| Poland | Hartleb (2012) ^(31)^ | National | N/R | Exhaustive | Study of elderly population, sample from national population register | >65, mean age 79∙4 | 3,826 | 2∙9% (2∙4 - 3∙5) |
| Poland | Flisiak (2011) ^(^[^3^](#_ENREF_63)^2)^ | Multi-centre | 2009-2010 | Convenience | Consecutive patients in a GP outpatient clinic, 65∙4% female | Mean age 45 | 1,203 | 0∙9% (0∙5 - 1∙6) |
| Poland | Pszenny (2012) ^(33)^ | Regional | 2000-2008 | Convenience | Retrospective study among deceased potential blood donors, regional in scope, 75% male | N/R | 4,733 | 2∙6% (2∙2 - 3∙1) |
| **Romania*** | Gheorghe (2010) ^(3^[^4^](#_ENREF_48)^)^ | National | 2006-2008 | Random | National cross-sectional pop. survey | 18 to 69 | 13,146 | 3∙2% (2∙9 - 3∙6) |
| **Slovakia*** | Schreter (2007) ^(^[^35^](#_ENREF_64)^)^ | National | 2002 | Random | Residual serum samples | 15 to 69 | 2,124 | 2∙0% (1∙4 - 2∙7) |
| **Spain*** | Lopez-Izquierdo (2007) ^(36)^ | Regional | 2003 | Random | Study in Valladolid, stratified random sampling based on health card | N/R | 364 | 1∙1% (0∙3 - 2∙8) |
| Spain | Caballeria (2014) ^(37)^ | Multi-centre | 2010-2011 | Random | Multi-centre study, invitation-based screening via GP registers in Barcelona | 20 to 90, mean age 50∙6 | 238 | 0∙4% (0∙0 - 2∙3) |
| Spain | Caballeria (2014) ^(^[^37^](#_ENREF_65)^)^ | Multi-centre | 2010-2011 | Convenience | Multi-centre study, screening via flyers and posters in GP offices in Barcelona | Mean age 51∙2 | 69 | 1∙5% (0∙0 - 7∙8) |
| Spain | Calleja-Panero (2013) ^(^[^38^](#_ENREF_52)^)^ | Multi-centre | 2007-2010 | Convenience | Study in Murcia and Madrid of working population at yearly insurance check-up, 73% male | 42 | 5,017 | 0∙6% (0∙4 - 0∙9) |
| United Kingdom | Pepas (2011) ^(39)^ | Local | 2007-2009 | Exhaustive | Patients undergoing Assisted Reproductive Treatment at Guy & St Thomas Hospital, London | N/R | 3,953 | 0∙4% (0∙3 - 0∙7) |
| United Kingdom | Balogun (2009) ^(40)^ | Regional | 2000 | Convenience | Residual serum samples from Public Health and National Health Service Laboratories in England and Wales for routine diagnostic examination | >16 | 5,068 | 1∙2% (0∙9 - 1∙5) |

# Estimates that were used for the pooled estimate

* Higher quality estimate

***References***

1. Quoilin S, Hutse V, Vandenberghe H, et al. A population-based prevalence study of hepatitis A, B and C virus using oral fluid in Flanders, Belgium. Eur J Epidemiol. 2007;22(3):195-202.

2. Burek V, Horvat J, Butorac K, Mikulic R. Viral hepatitis B, C and HIV infection in Croatian prisons. Epidemiol Infect. 2010;138(11):1610–20.

3. Vilibic-Cavlek T, Kucinar J, Ljubin-Sternak S, Kaic B, Lazaric-Stefanovic L, Kolaric B. Prevalence of viral hepatitis in Croatian adult population undergoing routine check-up, 2010–2011. Cent Eur J Public Health. 2014 Mar;22(1):29-33.

4. Meffre C, Le Strat Y, Delarocque-Astagneau E, et al. Prevalence of hepatitis B and hepatitis C virus infections in France in 2004: social factors are important predictors after adjusting for known risk factors. J Med Virol. 2010 Apr;82(4):546-55.

5. Sahajian F, Vanhems P, Bailly F, Fabry J, Trepo C, Sepetjan M. Screening campaign of hepatitis C among underprivileged people consulting in health centres of Lyon area, France. Eur J Public Health. 2007 Jun;17(3):263-71.

6. Poynard T, Lebray P, Ingiliz P, et al. Screening for liver advanced fibrosis using non-invasive biomarker fibrotest in general population. J Hepatol. 2009;50:S369-S70.

7. Poethko-Müller C, Zimmermann R, Hamouda O, et al. Die Seroepidemiologie der Hepatitis A, B und C in Deutschland. Ergebnisse der Studie zur Gesundheit Erwachsener in Deutschland (DEGS1). Bundesgesundheitsblatt Gesundheitsforschung Gesundheitsschutz. 2013;56:707–15.

8. Huetter ML, Fuchs M, Hanle MM, et al. Prevalence of risk factors for liver disease in a random population sample in southern Germany. Zeitschrift fur Gastroenterologie. 2014;52(6):558-63.

9. Wolffram I, Petroff D, Batz O, et al. Prevalence of elevated ALT values, HBsAg, and anti-HCV in the primary care setting and evaluation of guideline defined hepatitis risk scenarios. J Hepatol. 2015 Jun;62(6):1256-64.

10. Drositis I, Bertsias A, Lionis C, Kouroumalis E. Epidemiology and molecular analysis of hepatitis A, B and C in a semi-urban and rural area of Crete. Eur J Intern Med. 2013 Dec;24(8):839-45.

11. Treso B, Barcsay E, Tarjan A, et al. Prevalence and correlates of HCV, HVB, and HIV infection among prison inmates and staff, Hungary. J Urban Health. 2012 Feb;89(1):108-16.

12. Talento AF, Hickey D, O'Neill D, et al. Serological screening of solid organ transplant donors in Ireland. J Hosp Infect. 2010;76:S58-S9.

13. Cozzolongo R, Osella AR, Elba S, et al. Epidemiology of HCV infection in the general population: a survey in a southern Italian town. Am J Gastroenterol. 2009 Nov;104(11):2740-6.

14. Pendino GM, Mariano A, Surace P, et al. Prevalence and etiology of altered liver tests: a population-based survey in a Mediterranean town. Hepatology. 2005 May;41(5):1151-9.

15. Floreani A, Betterle C, Carderi I, et al. Is hepatitis C virus a risk factor for thyroid autoimmunity? J Viral Hepat. 2006 Apr;13(4):272-7.

16. Petti S, Divizia M, Donia D, et al. Analysis of the shift of the transmission pattern for hepatitis C in a community in Central Italy. New Microbiol. 2006 Jul;29(3):207-9.

17. Parisi MR, Soldini L, Vidoni G, et al. Point-of-care testing for HCV infection: recent advances and implications for alternative screening. New Microbiol. 2014;37(4):449-57.

18. Guadagnino V, Stroffolini T, Caroleo B, et al. Hepatitis C virus infection in an endemic area of Southern Italy 14 years later: evidence for a vanishing infection. Dig Liver Dis. 2013 May;45(5):403-7.

20. Fabris P, Baldo V, Baldovin T, et al. Changing epidemiology of HCV and HBV infections in Northern Italy: a survey in the general population. J Clin Gastroenterol. 2008 May-Jun;42(5):527-32.

21. Montella M, Crispo A, Grimaldi M, et al. Prevalence of hepatitis C virus infection in different population groups in southern Italy. Infection. 2005;33(1):9-12.

22. Del Corno G, Civardi E. Intrafamilial transmission of hepatitis B and C viruses in an Italian local health district. Ann Ig. 2006 Jul-Aug;18(4):287-95.

23. Dazzani F, Giacomoni P, Foschi FG, et al. Prevalence of transaminase abnormalities in general population in XXI century: Preliminary results from the Bagnacavallo study. Hepatology. 2009;50:794A-5A.

24. De Paschale M, Manco MT, Belvisi L, et al. Epidemiology of hepatitis D virus (HDV) infection in an urban area of northern Italy. Infection. 2012;40(5):485-91.

25. Squeri R, La Fauci V, Sindoni L, Cannavo G, Ventura Spagnolo E. Study on hepatitis B and C serologic status among municipal solid waste workers in Messina (Italy). J Prev Med Hyg. 2006 Sep;47(3):110-3.

26. Tolmane I, Rozentale B, Keiss J, Arsa F, Brigis G, Zvaigzne A. The prevalence of viral hepatitis C in Latvia: a population-based study. Medicina (Kaunas). 2011; 47(10):532-5.

27. Liakina V, Valantinas J. Anti-HCV prevalence in the general population of Lithuania. Med Sci Monit. 2012 Mar;18(3):PH28-35.

28. Vriend HJ, Van Veen MG, Prins M, Urbanus AT, Boot HJ, Op De Coul EL. Hepatitis C virus prevalence in Netherlands: migrants account for most infections. Epidemiol Infect. 2013;141(6):1310–7.

29. Veldhuijzen IK, van Driel HF, Vos D, et al. Viral hepatitis in a multi-ethnic neighborhood in the Netherlands: results of a community-based study in a low prevalence country. Int J Infect Dis. 2009 Jan;13(1):e9-e13.

30. Slavenburg S, Verduyn-Lunel FM, Hermsen JT, Melchers WJ, te Morsche RH, Drenth JP. Prevalence of hepatitis C in the general population in the Netherlands. Neth J Med. 2008;66(1):13–7.

31. Hartleb M, Gutkowski K, Zejda JE, Chudek J, Wiecek A. Serological prevalence of hepatitis B virus and hepatitis C virus infection in the elderly population: Polish nationwide survey–PolSenior. Eur J Gastroenterol Hepatol. 2012 Nov;24(11):1288-95.

32. Flisiak R, Halota W, Horban A, Juszczyk J, Pawlowska M, Simon K. Prevalence and risk factors of HCV infection in Poland. Eur J Gastroenterol Hepatol. 2011 Nov;23(12):1213-7.

33. Pszenny A, Hreńczuk M, Czerwiński J, Danielewicz R, Małkowski P. Epidemiologia zakażeń wirusami zapalenia wątroby typu B i C wśród zmarłych dawców narządów w Polsce. Probl Hig Epidemiol. 2012;93(3):579-85.

34. Gheorghe L, Csiki IE, Iacob S, Gheorghe C. The prevalence and risk factors of hepatitis B virus infection in an adult population in Romania: a nationwide survey. Eur J Gastroenterol Hepatol. 2013;25(1):56-64.

35. Schreter I, Kristian P, Klement C, et al. [Prevalence of hepatitis C virus infection in Slovakia]. Klin Mikrobiol Infekc Lek. 2007;13(2):54–8.

36. Lopez-Izquierdo R, Udaondo MA, Zarzosa P, et al. [Seroprevalence of viral hepatitis in a representative general population of an urban public health area in Castilla y Leon (Spain)]. Enferm Infecc Microbiol Clin. 2007;25(5):317-23.

37. Caballeria L, Pera G, Bernad J, Canut S, Navarro E, Bruguera M. Strategies for the detection of hepatitis C viral infection in the general population. Rev Clin Esp (Barc). 2014 Jun-Jul;214(5):242-6.

38. Calleja-Panero JL, Llop-Herrera E, Ruiz-Moraga M, et al. Prevalence of viral hepatitis (B and C) serological markers in healthy working population. Rev Esp Enferm Dig. 2013 May-Jun;105(5):249-54.

39. Pepas L, Macmahon E, El Toukhy T, Khalaf Y, Braude P. Viral screening before each cycle of assisted conception treatment is expensive and unnecessary: a survey of results from a UK inner city clinic. Hum Fertil (Camb). 2011 Dec;14(4):224-9.

40. Balogun MA, Vyse AJ, Hesketh LM, Kafatos G, Parry JV, Ramsay ME. Estimating hepatitis C infection acquired in England, 1986–2000. Epidemiol Infect. 2009;137(9):1249-54.

**Supplementary Table S9.** Risk of bias assessment and characteristics of the general population studies included in the HBV and HCV prevalence analysis

| ***Study Characteristics*** | | | | ***Risk of Bias Assessment*** | | | | |
| --- | --- | --- | --- | --- | --- | --- | --- | --- |
| **Authors** | **Publication Year** | **Virus** | **Country** | **Age Bias (0 or 1)** | **Gender Bias**  **(0 or 1)** | **Sampling method**  **(0, 1 or 2)** | **Population coverage**  **(0, 1 or 2)** | **Total**  **Score**  **(0 - 6)** |
| Quoilin | 2007 | Both | Belgium | 1 | 1 | 1 | 1 | 4 |
| Nardone | 2009 | HBV | Belgium | 0 | 1 | 0 | 2 | 3 |
| Vilibic-Cavlek | 2014 | Both | Croatia | 1 | 1 | 0 | 2 | 4 |
| Burek | 2010 | Both | Croatia | 1 | 0 | 1 | 1 | 3 |
| Nardone | 2009 | HBV | Czech Republic | 0 | 1 | 1 | 2 | 4 |
| Meffre | 2010 | Both | France | 1 | 1 | 1 | 2 | 5 |
| Sahajian | 2007 | HCV | France | 1 | 1 | 0 | 1 | 3 |
| Bottero | 2014 | HBV | France | 1 | 1 | 0 | 1 | 3 |
| Poynard | 2009 | HCV | France | 0 | 1 | 0 | 1 | 2 |
| Huetter | 2014 | Both | Germany | 1 | 1 | 2 | 0 | 4 |
| Poethko-Müller | 2013 | Both | Germany | 1 | 1 | 1 | 2 | 5 |
| Wolffram | 2015 | Both | Germany | 0 | 1 | 0 | 1 | 2 |
| Dounias | 2005 | HBV | Greece | 1 | 0 | 0 | 0 | 1 |
| Drositis | 2013 | Both | Greece: Crete | 1 | 1 | 2 | 0 | 4 |
| Treso | 2012 | Both | Hungary | 1 | 1 | 0 | 2 | 4 |
| Nardone | 2009 | HBV | Ireland | 0 | 1 | 0 | 2 | 3 |
| Talento | 2010 | Both | Ireland | 1 | 1 | 2 | 2 | 6 |
| Floreani | 2006 | Both | Italy | 1 | 1 | 2 | 0 | 4 |
| Fabris | 2008 | Both | Italy | 1 | 1 | 1 | 0 | 3 |
| Squeri | 2006 | Both | Italy | 1 | 0 | 0 | 0 | 1 |
| Da Villa | 2007 | HBV | Italy | 1 | 1 | 0 | 0 | 2 |
| Dazzani | 2009 | Both | Italy | 1 | 1 | 0 | 0 | 2 |
| Boccalini | 2013 | HBV | Italy | 1 | 1 | 0 | 1 | 3 |
| Guadagnino | 2013 | HCV | Italy | 1 | 1 | 1 | 0 | 3 |
| Montella | 2005 | HCV | Italy | 1 | 1 | 0 | 1 | 3 |
| Pendino | 2005 | Both | Italy | 1 | 1 | 2 | 0 | 4 |
| Cozzolongo | 2009 | Both | Italy | 1 | 1 | 2 | 1 | 5 |
| De Paschale | 2012 | Both | Italy | 1 | 1 | 0 | 0 | 2 |
| Parisi | 2014 | HCV | Italy | 1 | 1 | 0 | 1 | 3 |
| Petti | 2006 | HCV | Italy | 1 | 1 | 2 | 0 | 4 |
| Petti | 2006 | HCV | Italy | 1 | 1 | 2 | 0 | 4 |
| Petti | 2006 | HCV | Italy | 1 | 1 | 2 | 0 | 4 |
| Giacomoni | 2010 | Both | Italy | 0 | 1 | 0 | 0 | 1 |
| Del Corno | 2006 | Both | Italy | 1 | 1 | 1 | 0 | 3 |
| Tolmane | 2011 | HCV | Latvia | 1 | 1 | 2 | 2 | 6 |
| Liakina | 2012 | HCV | Lithuania | 0 | 0 | 0 | 2 | 2 |
| Pszenny | 2012 | Both | Poland | 0 | 0 | 0 | 1 | 1 |
| Flisiak | 2011 | HCV | Poland | 1 | 0 | 0 | 1 | 2 |
| Hartleb | 2012 | Both | Poland | 0 | 1 | 1 | 2 | 4 |
| Nardone | 2009 | HBV | Romania | 0 | 1 | 0 | 2 | 3 |
| Gheorghe | 2013 | HBV | Romania | 1 | 1 | 2 | 2 | 6 |
| Gheorghe | 2010 | HCV | Romania | 1 | 1 | 2 | 2 | 6 |
| Nardone | 2009 | HBV | Slovakia | 0 | 1 | 1 | 2 | 4 |
| Schreter | 2007 | HCV | Slovakia | 1 | 1 | 2 | 2 | 6 |
| Salleras | 2007 | HBV | Spain | 0 | 1 | 2 | 0 | 3 |
| Salleras | 2007 | HBV | Spain | 1 | 1 | 2 | 0 | 4 |
| Salleras | 2007 | HBV | Spain | 0 | 1 | 2 | 0 | 3 |
| Lopez-Izquierdo | 2007 | Both | Spain | 1 | 1 | 2 | 0 | 5 |
| Calleja-Panero | 2013 | Both | Spain | 0 | 1 | 0 | 1 | 2 |
| Pedraza-Flechas | 2014 | HBV | Spain | 1 | 1 | 2 | 1 | 5 |
| Caballeria | 2014 | HCV | Spain | 1 | 1 | 1 | 0 | 3 |
| Caballeria | 2014 | HCV | Spain | 1 | 1 | 0 | 0 | 2 |
| Veldhuijzen | 2009 | Both | The Netherlands | 1 | 1 | 1 | 0 | 3 |
| Slavenburg | 2008 | HCV | The Netherlands | 1 | 1 | 0 | 0 | 2 |
| Hahné | 2012 | HBV | The Netherlands | 1 | 1 | 1 | 2 | 5 |
| Vriend | 2013 | HCV | The Netherlands | 1 | 1 | 1 | 2 | 5 |
| Pepas | 2011 | Both | UK: England | 0 | 1 | 2 | 0 | 3 |
| Balogun | 2009 | HCV | UK: England & Wales | 1 | 1 | 0 | 1 | 3 |

# Estimates that were used for the pooled estimate

* Higher quality estimate

**Supplementary Table S10.** Extracted and pooled (where possible) HBsAg prevalence estimates for pregnant women from included studies

| **Country** | **Author**  **(Publ. Year)** | **Geographical coverage** | **Sampling period** | **Sampling method** | **Study population details** | **Age range** | **Sample size** | **Prevalence estimate (95%CI)** |
| --- | --- | --- | --- | --- | --- | --- | --- | --- |
| Denmark# | Moller (2014) ^(1)^ | National | 2013 | Exhaustive | National antenatal screening programme | N/R | 60,977 | 0∙3% (0∙2 - 0∙3) |
| Denmark# | Harder (2011) ^(2)^ | National | 2005-2007 | Exhaustive | National antenatal screening programme | N/R | 140,376 | 0∙3% (0∙2 - 0∙3) |
| **Denmark*** | Pooled | Pooled | 2005-2007; 2013 | Pooled | Pooled | Pooled | 201,353 | 0∙3% (0∙2 – 0∙3) |
| **France*** | Richaud-Eyraud (2015) ^(3)^ | National | 2011 | N/R | ELFE cohort study, national sample | 25 to 34, mean age 30 | N/R | 0∙8% (0∙6 - 1∙1) |
| France | Braillon (2010) ^(4)^ | Regional | 2006 | Random | Regional coverage, lookback study design | Mean age 29 | 1,112 | 0∙2% (0∙0 - 0∙6) |
| Germany | Lobstein (2011) ^(5)^ | Local | 2006-2010 | Exhaustive | Deliveries in one hospital | N/R | 8,193 | 0∙5% (0∙3 - 0∙7) |
| Germany | Alba-Alejandre (2008) ^(6)^ | Local | 2001-2008 | Exhaustive | Antenatal screening in Leutkirch hospitals, South Germany | N/R | 15,873 | 0∙8% (0∙7 - 1∙0) |
| **Greece*** | Papaevangelou (2006) ^(7)^ | National | 2003 | Exhaustive | National antenatal screening programme | N/R | 3,384 | 2∙9% (2∙4 -3∙5) |
| Greece | Karatapanis (2012) ^(8)^ | Local | 2009-2011 | Exhaustive | Antenatal screening attendees | Mean age 27∙2 | 1,304 | 1∙2% (0∙6 - 1∙9) |
| Greece | Karatapanis (2012) ^(76)^ | Local | 2009-2011 | Exhaustive | Antenatal screening non-attendees screened post-partum | Mean age 26∙6 | 1,000 | 5∙3% (4∙0 - 6∙9) |
| Greece | Elefsiniotis (2010) ^(9)^ | Local | 2008-2009 | Exhaustive | Consecutive women delivering at the ObGyn department | N/R | 1,826 | 3∙8% (3∙0 - 4∙8) |
| Greece | Kafkoula (2009) ^(10)^ | Local | 2005-2007 | Exhaustive | Antenatal screening at Thriassio General Hospital | N/R | 2,188 | 0∙0% (0∙0 - 0∙3) |
| Greece | Betsas (2006) ^(11)^ | Local | 2004 | Exhaustive | Attendees of the University of Thessaloniki antenatal clinic | Mean age 32∙3 (Greek-born); 27∙1 (Migrant) | 544 | 3∙5% (2∙1 - 5∙4) |
| Ireland | Connell (2010) ^(12)^ | Local | 2004-2009 | Exhaustive | Antenatal screening lab data from Galway hospital | N/R | 24,008 | 0∙2% (0∙2 - 0∙4) |
| Italy# | Spada (2014) ^(13)^ | Multi-centre | 2008-2009 | Exhaustive | Multi-centre study in 41 hospitals across 13 regions | 14 to 53 | 16,858 | 0∙9% (0∙7 - 1∙0) |
| Italy# | Ruffini (2014) ^(14)^ | Regional | 2011-2012 | Exhaustive | Regional study, one-third migrant women | >17 | 10,093 | 0∙8% (0∙6 - 1∙0) |
| **Italy*** | Pooled | Pooled | 2008-2009; 2011-2012 | Pooled | Pooled | Pooled | 26,951 | 0∙8% (0∙7 – 1∙0) |
| The Netherlands# | Op de Coul (2011) ^(15)^ | National | 2006 | Exhaustive | National antenatal screening programme | N/R | 186,137 | 0∙3% (0∙3 - 0∙4) |
| The Netherlands# | Op de Coul (2011) ^(15)^ | National | 2007 | Exhaustive | National antenatal screening programme | N/R | 190,140 | 0∙3% (0∙3 - 0∙3) |
| The Netherlands# | Op de Coul (2011) ^(15)^ | National | 2008 | Exhaustive | National antenatal screening programme | N/R | 185,941 | 0∙4% (0∙4 - 0∙4) |
| **The Netherlands*** | Pooled | Pooled | 2006-2008 | Pooled | Pooled | Pooled | 562,218 | 0∙3% (0∙3 – 0∙4) |
| **Norway*** | Kristiansen (2009) ^(16)^ | Regional | 2003-2004 | Exhaustive | Multi-centre study in all hospitals and delivery rooms in Northern Norway | 16 to 44, mean age 29∙3 | 1,668 | 0∙1% (0∙0 - 0∙3) |
| Slovakia | Kristian (2013) ^(17)^ | Regional | 2008-2009 | Convenience | Residual serum samples from regional departments of clinical microbiology, Eastern Slovakia | N/R | 13,798 | 2∙1% (1∙9 - 2∙4) |
| Slovakia | Kristian (2010) ^(18)^ | Regional | 2000-2004 | Convenience | Residual serum samples from nine regional departments of clinical microbiology, Eastern Slovakia | N/R | 10,739 | 2∙3% (2∙1 - 2∙6) |
| **Spain*** | Salleras (2009) ^(19)^ | Regional | 2008-2009 | Random /  Exhaustive | Regional antenatal screening programme | 15 to 49 | 1,534 | 0∙1% (0∙0 - 0∙5) |
| Spain | Lopez-Fabal (2013) ^(20)^ | Local | 2007-2010 | Convenience | Study in a hospital and a health centre in Madrid | 19 to 49, mean age 30 | 6,939 | 0∙9% (0∙6 - 1∙1) |
| Spain | Sampedro (2010) ^(21)^ | Local | 2007-2008 | Exhaustive | Single centre study, 8∙4% migrants | N/R | 4,169 | 0∙6% (0∙4 - 0∙9) |
| United Kingdom# | Schnier (2014) ^(22)^ | National | 2009-2010 | Exhaustive | Antenatal screening from 4 laboratories (covering 60% of Scottish population) | 15 to 44 | 129,171 | 0∙3% (0∙3 - 0∙3) |
| United Kingdom# | Godbole (2013) ^(23)^ | Local | 2007-2010 | Exhaustive | Antenatal screening in 4 London hospitals | 15 to 46, mean age 29 | 38,227 | 1∙0% (0∙9 - 1∙2) |
| United Kingdom | Pepas (2011) ^(24)^ | Local | 2003 | N/R | Antenatal screening at Guy & St Thomas Hospital, London | N/R | 25,082 | 1∙4% (1∙3 - 1∙6) |
| **United Kingdom*** | Pooled | Pooled | 2007-2010 | Pooled | Pooled | Pooled | 167,398 | 0∙5% (0∙4 – 0∙5) |

# Estimates that were used for the pooled estimate

* Higher quality estimate

***References***

1. Statens Serum Institut. Hepatitis B, HIV and syphilis screening of pregnant women, 2013. Statens Serum Institut 2014 [cited 2015 25 May]. Available from: http://www.ssi.dk/English/News/EPINEWS/2014/No%2022%20-%202014.aspx.

2. Harder KM, Cowan S, Eriksen MB, Krarup HB, Christensen PB. Universal screening for hepatitis B among pregnant women led to 96% vaccination coverage among newborns of HBsAg positive mothers in Denmark. Vaccine. 2011;29(50):9303–7.

3. Richaud-Eyraud E, Brouard C, Antona D, La Ruche G, Tourdjman M, Dufourg MN, Lot F. Infectious diseases screening during pregnancy: results from the ELFE survey in maternity units, mainland France, 2011. Bull Epidemiol Hebd 2015;15-16.

4. Braillon A, Nguyen-Khac E, Merlin J, Dubois G, Gondry J, Capron D. [HBsAg screening during pregnancy in the French province Picardy Gynecol Obstet Fertil. 2010 Jan;38(1):13-7.

5. Lobstein S, Faber R, Tillmann HL. Prevalence of hepatitis B among pregnant women and its impact on pregnancy and newborn complications at a tertiary hospital in the eastern part of Germany. Digestion. 2011;83(1- 2):76-82.

6. Alba-Alejandre I, Kainer F, Friese K, Mylonas I. HBsAg screening during pregnancy. Geburtshilfe Frauenheilkunde. 2009;69(6):537-40.

7. Papaevangelou V, Hadjichristodoulou C, Cassimos D, Theodoridou M. Adherence to the screening program for HBV infection in pregnant women delivering in Greece. BMC Infect Dis. 2006 May 9;6:84.

8. Karatapanis S, Skorda L, Marinopoulos S, et al. Higher rates of chronic hepatitis B infection and low vaccination-induced protection rates among parturients escaping HBsAg prenatal testing in Greece: a 2-year prospective study. Eur J Gastroenterol Hepatol. 2012 Aug;24(8):878-83.

9. Elefsiniotis I, Tsoumakas K, Vezali E, Glynou I, Drakoulis N, Saroglou G. Spontaneous preterm birth in women with chronic hepatitis B virus infection. Int J Gynaecol Obstet. 2010 Sep;110(3):241-4.

10. Kafkoula H, Kouvardas S, Kydonopoulou A, et al. Evaluation of infectious diseases in pregnant women in Thriassio region during a period of years. Vox Sang. 2009;96:176–7.

11. Betsas G, Karamba S, Daniilidis A, et al. Prevalence of hepatitis B virus (HBV) inflammation in pregnant women in Northern Greece. Eur J Inflamm. 2006;4(2):125–8.

12. O'Connell K, Cormican M, Hanahoe B, Smyth B. Prevalence of antenatal hepatitis B virus carriage in the west of Ireland. Ir Med J. 2010 Mar;103(3):91-2.

13. Spada E, Tosti ME, Zuccaro O, Stroffolini T, Mele A. Evaluation of the compliance with the protocol for preventing perinatal hepatitis B infection in Italy. J Infect. 2011 Feb;62(2):165-71.

14. Ruffini E, Compagnoni L, Tubaldi L, et al. [Congenital and perinatal infections in the Marche region (Italy): an epidemiological study and differences between ethnic groups]. Infez Med. 2014 Sep;22(3):213-21.

15. Op de Coul EL, Hahné S, van Weert YW, et al. Antenatal screening for HIV, hepatitis B and syphilis in the Netherlands is effective. BMC Infect Dis. 2011 Jun 30;11:185.

16. Kristiansen MG, Eriksen BO, Maltau JM, et al. Prevalences of viremic hepatitis C and viremic hepatitis B in pregnant women in northern Norway. Hepatogastroenterology. 2009 Jul- Aug;56(93):1141-5.

17. Kristian P, Veselska ZD, Paralicova Z, et al. Regional and ethnic aspects of viral hepatitis B among pregnant women. Cent Eur J Public Health. 2013 Mar;21(1):22-5.

18. Kristian P, Schreter I, Paralicova Z, Jarcuska P, Siegfried L, Porubcin S. Regional and ethnical aspects of viral hepatitis B in pregnant women in Slovakia. Int J Infect Dis. 2010;14:e227-e8.

19. Salleras L, Dominguez A, Bruguera M, et al. Seroepidemiology of hepatitis B virus infection in pregnant women in Catalonia (Spain). J Clin Virol. 2009 Apr;44(4):329-32.

20. Lopez-Fabal F, Gomez-Garces JL. [Serological markers of Spanish and immigrant pregnant women in the south of Madrid during the period 2007–2010]. Rev Esp Quimioter. 2013 Jun;26(2):108-11.

21. Sampedro A, Mazuelas P, Rodriguez-Granger J, Torres E, Puertas A, Navarro JM. [Serological markers in immigrant and Spanish pregnant women in Granada]. Enferm Infecc Microbiol Clin. 2010 Dec;28(10):694-7.

22. Schnier C, Wallace L, Tempelton K, et al. Use of laboratory-based surveillance data to estimate the number of people chronically infected with hepatitis B living in Scotland. Epidemiol Infect. 2014 Oct;142(10):2121-30.

23. Godbole G, Irish D, Basarab M, et al. Management of hepatitis B in pregnant women and infants: a multicentre audit from four London hospitals. BMC Pregnancy Childbirth. 2013 Dec 1;13:222.

24. Pepas L, Macmahon E, El Toukhy T, Khalaf Y, Braude P. Viral screening before each cycle of assisted conception treatment is expensive and unnecessary: a survey of results from a UK inner city clinic. Hum Fertil (Camb). 2011 Dec;14(4):224-9.

**Supplementary Table S11.** Extracted and pooled (where possible) anti-HCV prevalence estimates for pregnant women from included studies

| **Country** | **Author (Publ. Year)** | **Geographical coverage** | **Sampling period** | **Sampling method** | **Study population details** | **Age range** | **Sample size** | **Prevalence estimate (95%CI)** |
| --- | --- | --- | --- | --- | --- | --- | --- | --- |
| Austria | Diab-Elschahawi (2013) ^(1)^ | Local | 2009-2011 | Exhaustive | Universal antenatal screening at Vienna University Hospital | 18 to 43 | 4,222 | 1∙7% (1∙4 - 2∙2) |
| Greece | Kafkoula (2009) ^(2)^ | Local | 2005-2007 | Exhaustive | Antenatal screening at Thriassio General Hospital | N/R | 2,188 | 1∙3% (0∙9 - 1∙8) |
| Ireland | Lambert (2013) ^(3)^ | Local | 2007-2008 | Exhaustive | Antenatal care attendees of Rotunda hospital | N/R | 8,976 | 0∙9% (0∙7 - 1∙1) |
| Ireland | Martyn (2011) ^(4)^ | Local | 2006 | Exhaustive | Deliveries at a single hospital | N/R | 4,666 | 1∙4% (1∙1 - 1∙8) |
| Ireland | Martyn (2011) ^(4)^ | Local | 2007 | Exhaustive | Deliveries at a single hospital | N/R | 9,222 | 0∙7% (0∙6 - 0∙9) |
| Italy | Ruffini (2014) ^(5)^ | Regional | 2011-2012 | Exhaustive | Regional study, one third migrant women | >17 | 9,977 | 0∙4% (0∙3 - 0∙5) |
| Italy | Veronesi (2007) ^(6)^ | Local | 1996-2001 | Exhaustive | All deliveries at the hospital in Palma | N/R | 13,025 | 0∙8% (0∙7 - 1∙0) |
| Italy | Lagana (2015) ^(7)^ | Local | 2003-2013 | Convenience | Out-patient clinic attendees, mostly migrant women | N/R | 320 | 0∙9% (0∙2 - 2∙7) |
| The Netherlands | Urbanus (2011) ^(8)^ | Local | 2003 | Random | Antenatal screening in Amsterdam, 64% non-Dutch | >15 | 4,563 | 0∙3% (0∙2 - 0∙5) |
| Norway | Kristiansen (2009) ^(9)^ | Regional | 2003-2004 | Exhaustive | Multi-centre study in all hospitals and delivery rooms in Northern Norway | 16 to 44, mean age 29∙3 | 1,668 | 0∙9% (0∙5 - 1∙5) |
| Slovenia | Kopilovic (2015) ^(10)^ | National | 2013 | Exhaustive | Residual sera from antenatal screening | N/R | 9,574 | 0∙1% (0∙1 - 0∙2) |
| Slovenia | Kopilovic (2015) ^(10)^ | National | 2009 | Exhaustive | Residual sera from antenatal screening | N/R | 8,064 | 0∙1% (0∙0 - 0∙2) |
| Slovenia | Kopilovic (2015) ^(10)^ | National | 2003 | Exhaustive | Residual sera from antenatal screening | N/R | 7,281 | 0∙2% (0∙1 - 0∙3) |
| Slovenia | Kopilovic (2015) ^(10)^ | National | 2003, 2009 & 2013 | Exhaustive | Residual sera from antenatal screening | N/R | 24,919 | 0∙1% (0∙1 - 0∙2) |
| Spain | Seisdedos (2011) ^(11)^ | Multi-centre  (6 regions) | 2012 | Random | HIV- women screened in 6 regions | N/R | 8,555 | 0∙2% (0∙1 - 0∙3) |

# Estimates that were used for the pooled estimate

* Higher quality estimate

***References***

1. Diab-Elschahawi M, Dosch V, Honsig C, et al. Evaluation of a universal vs a targeted hepatitis C virus screening strategy among pregnant women at the Vienna University Hospital. Am J Infect Control. 2013 May;41(5):459-60.

2. Kafkoula H, Kouvardas S, Kydonopoulou A, et al. Evaluation of infectious diseases in pregnant women in Thriassio region during a period of years. Vox Sang. 2009;96:176–7.

3. Lambert J, Jackson V, Coulter-Smith S, et al. Universal antenatal screening for hepatitis C. Ir Med J. 2013;106(5):136–9.

4. Martyn F, Phelan O, O'Connell M. Hepatitis C: is there a case for universal screening in pregnancy? Ir Med J. 2011;104(5):144–6.

5. Ruffini E, Compagnoni L, Tubaldi L, et al. [Congenital and perinatal infections in the Marche region (Italy): an epidemiological study and differences between ethnic groups]. Infez Med. 2014 Sep;22(3):213-21.

6. Veronesi L, Verrotti Di Pianella C, Benassi L, Benaglia G, Affanni P, Tanzi ML. Mother to child transmission of hepatitis C virus in a province of northern Italy. J Prev Med Hyg. 2007 Jun;48(2):47-9.

7. Lagana AS, Gavagni V, Musubao JV, Pizzo A. The prevalence of sexually transmitted infections among migrant female patients in Italy. Int J Gynaecol Obstet. 2015 Feb;128(2):165-8.

8. Urbanus AT, van de Laar TJ, van den Hoek A, et al. Hepatitis C in the general population of various ethnic origins living in the Netherlands: Should non-Western migrants be screened? J Hepatol. 2011 Dec;55(6):1207-14.

9. Kristiansen MG, Eriksen BO, Maltau JM, et al. Prevalences of viremic hepatitis C and viremic hepatitis B in pregnant women in northern Norway. Hepatogastroenterology. 2009 Jul- Aug;56(93):1141-5.

10. Kopilović BPM, Seme K, Klavs I. Hepatitis C virus infection among pregnant women in Slovenia: study on 31,849 samples obtained in four screening rounds during 1999, 2003, 2009 and 2013. Euro Surveill. 2015 Jun 4;20(22):21144.

11. Seisdedos T, Diaz A, Bleda MJ, Ortiz M, Garcia A, Diez M. Prevalence of maternal hepatitis C infection according to HIV serostatus in six Spanish regions (2003–2006). Eur J Public Health. 2011 Oct;21(5):643-5.

**Supplementary Table S12.** Risk of bias assessment and characteristics of the studies in pregnant women included in the HBV and HCV prevalence analysis

| ***Study Characteristics*** | | | | ***Risk of Bias Assessment*** | | |
| --- | --- | --- | --- | --- | --- | --- |
| **Authors** | **Publication Year** | **Virus** | **Country** | **Sampling method  (0 or 1)** | **Population coverage (0, 1 or 2)** | **Total Score**  **(0 - 3)** |
| Diab-Elschahawi | 2013 | HCV | Austria | 1 | 0 | 1 |
| Moller | 2014 | HBV | Denmark | 1 | 2 | 3 |
| Harder | 2011 | HBV | Denmark | 1 | 2 | 3 |
| Braillon | 2010 | HBV | France | 1 | 0 | 1 |
| Richaud-Eyraud | 2015 | HBV | France | 1 | 1 | 2 |
| Alba-Alejandre | 2009 | HBV | Germany | 1 | 0 | 1 |
| Lobstein | 2011 | HBV | Germany | 1 | 0 | 1 |
| Papaevangelou | 2006 | HBV | Greece | 1 | 2 | 3 |
| Betsas | 2006 | HBV | Greece | 1 | 0 | 1 |
| Kafkoula | 2009 | Both | Greece | 1 | 0 | 1 |
| Elefsiniotis | 2010 | HBV | Greece | 1 | 0 | 1 |
| Karatapanis | 2012 | HBV | Greece | 1 | 0 | 1 |
| Karatapanis | 2012 | HBV | Greece | 1 | 0 | 1 |
| Martyn | 2011 | HCV | Ireland | 1 | 0 | 1 |
| Martyn | 2011 | HCV | Ireland | 1 | 0 | 1 |
| O'Connell | 2010 | HBV | Ireland | 1 | 0 | 1 |
| Lambert | 2013 | HCV | Ireland | 1 | 0 | 1 |
| Veronesi | 2007 | HCV | Italy | 1 | 0 | 1 |
| Lagana | 2015 | Both | Italy | 0 | 0 | 0 |
| Spada | 2011 | HBV | Italy | 1 | 2 | 3 |
| Ruffini | 2014 | HBV | Italy | 1 | 1 | 2 |
| Ruffini | 2014 | HCV | Italy | 1 | 1 | 2 |
| Kristiansen | 2009 | HBV | Norway | 1 | 1 | 2 |
| Kristian | 2010 | HBV | Slovakia | 0 | 1 | 1 |
| Kristian | 2013 | HBV | Slovakia | 0 | 1 | 1 |
| Kopilovic | 2015 | HCV | Slovenia | 1 | 2 | 3 |
| Kopilovic | 2015 | HCV | Slovenia | 1 | 2 | 3 |
| Kopilovic | 2015 | HCV | Slovenia | 1 | 2 | 3 |
| Kopilovic | 2015 | HCV | Slovenia | 1 | 2 | 3 |
| Kopilovic | 2015 | HCV | Slovenia | 1 | 2 | 3 |
| Lopez-Fabal | 2013 | HBV | Spain | 0 | 1 | 1 |
| Seisdedos | 2011 | HCV | Spain | 1 | 2 | 3 |
| Sampedro | 2010 | HBV | Spain | 1 | 0 | 1 |
| Salleras | 2009 | HBV | Spain | 1 | 1 | 2 |
| Op de Coul | 2011 | HBV | The Netherlands | 1 | 2 | 3 |
| Op de Coul | 2011 | HBV | The Netherlands | 1 | 2 | 3 |
| Op de Coul | 2011 | HBV | The Netherlands | 1 | 2 | 3 |
| Urbanus | 2011 | HCV | The Netherlands | 1 | 0 | 1 |
| Pepas | 2011 | Both | UK, England | 1 | 0 | 1 |
| Godbole | 2013 | HBV | UK, England | 1 | 1 | 2 |
| Schnier | 2014 | HBV | UK, Scotland | 1 | 1 | 2 |

**Supplementary Table S13.** Data used for HBsAg and anti-HCV prevalence estimate for the EU/EAA

| **Countries** | **Total Population*** | **HBV prevalence estimate (95%CI)** | **Estimated number of chronic HBV cases** | **HCV prevalence estimate (95%CI)** | **Estimated number of chronic HCV cases** | **Source HBV prevalence estimate** | **Source HCV prevalence estimate** |
| --- | --- | --- | --- | --- | --- | --- | --- |
| **Austria** | 8,506,889 | 0∙099% (0∙072 – 0∙132) | 8,422 | 0∙039% (0∙023 – 0∙061) | 2,322 | Blood donor data | Blood donor data |
| **Belgium** | 11,203,992 | 0∙7% (0∙5-0∙8) | 78,428 | 0∙1% (0∙0-0∙4) | 7,843 | Higher-quality estimates | Higher-quality estimates |
| **Bulgaria** | 7,245,677 | 3∙224% (3∙039 - 3∙418) | 233,601 | 0∙342% (0∙282 – 0∙410) | 17,346 | Blood donor data | Blood donor data |
| **Croatia** | 4,246,809 | 0∙7% (0∙5-0∙9) | 29,728 | 0∙9% (0∙6-1∙5) | 26,755 | Higher-quality estimates | Higher-quality estimates |
| **Cyprus** | 858,000 | 0∙441% (0∙270 - 0∙681) | 3784 | 0∙221% (0∙106 – 0∙405) | 1,327 | Blood donor data | Blood donor data |
| **Czech Republic**** | 10,512,419 | 0∙6% (0∙34-0∙99) | 63,075 | 0∙216% (0∙177 – 0∙261) | 15,895 | Higher-quality estimates | Blood donor data |
| **Denmark** | 5,627,235 | 0∙016% (0∙004 – 0∙040) | 900 | 0∙016% (0∙004 – 0∙040) | 630 | Blood donor data | Blood donor data |
| **Estonia** | 1,315,819 | 0∙267% (0∙128 – 0∙490) | 3,513 | 0∙959% (0∙673 – 1∙326) | 8,833 | Blood donor data | Blood donor data |
| **Finland** | 5,451,270 | 0∙000% (0∙000 – 0∙019) | 0 | 0∙025% (0∙008 – 0∙059) | 954 | Blood donor data | Blood donor data |
| **France** | 65,835,579 | 0∙7% (0∙5-0∙9) | 460,849 | 0∙8% (0∙7-1∙1) | 368,679 | Higher-quality estimates | Higher-quality estimates |
| **Germany** | 80,767,463 | 0∙4% (0∙3-0∙5) | 323,070 | 0∙4% (0∙3-0∙5) | 226,149 | Higher-quality estimates | Higher-quality estimates |
| **Greece** | 10,926,807 | 3∙3% (2∙2-4∙7 | 360,585 | 2∙2% (1∙3-3∙4) | 168,273 | Higher-quality estimates | Higher-quality estimates |
| **Hungary** | 9,877,365 | 0∙4% (0∙1-1∙0) | 39,509 | 0∙5% (0∙2-1∙1) | 34,571 | Higher-quality estimates | Higher-quality estimates |
| **Iceland** | 325,671 | 0∙072% (0∙002 – 0∙398) | 234 | 0∙000% (0∙000 – 0∙264) | 0 | Blood donor data | Blood donor data |
| **Ireland** | 4,605,501 | 0∙1% (0∙0-0∙4) | 4,606 | 0∙1% (0∙0-0∙4) | 3,224 | Higher-quality estimates | Higher-quality estimates |
| **Italy** | 60,782,668 | 0∙7% (0∙4-1∙0) | 425,479 | 5∙9% (5∙2-6∙6) | 2,510,324 | Higher-quality estimates | Higher-quality estimates |
| **Latvia***** | 2,001,468 | 1,127% | 22,557 | 2∙4% (1∙7-3∙3) | 33,625 | Blood donor data | Higher-quality estimates |
| **Liechtenstein** | 37,129 | - | - | - | - | - | - |
| **Lithuania** | 2,943,472 | 0∙560% (0∙468 – 0∙665) | 16,483 | 2∙9% (2∙10-3∙85) | 59,752 | Blood donor data | Lower-quality estimates |
| **Luxembourg** | 549,680 | 0∙000% (0∙000 – 0∙406) | 0 | 0∙221% (0∙027 – 0∙794) | 850 | Blood donor data | Blood donor data |
| **Malta** | 425,384 | 0∙174% (0∙047 – 0∙445) | 740 | 0∙043% (0∙001 – 0∙242) | 128 | Blood donor data | Blood donor data |
| **The Netherlands** | 16,829,289 | 0∙2% (0∙1-0∙4) | 33,659 | 0∙1% (0∙0-0∙2) | 11,781 | Higher-quality estimates | Higher-quality estimates |
| **Norway** | 5,107,970 | 0∙028% (0∙009 – 0∙065) | 1,430 | 0∙033% (0∙012 – 0∙073) | 1,180 | Blood donor data | Blood donor data |
| **Poland** | 38,017,856 | 0∙450% (0∙425 – 0∙476) | 171,080 | 2∙9% (2∙4-3∙5) | 771,762 | Blood donor data | Lower-quality estimates |
| **Portugal***** | 10,427,301 | 0∙094% | 9,802 | 0∙165% | 12,044 | Blood donor data | Blood donor data |
| **Romania** | 19,947,311 | 4∙4% (4∙0-4∙8) | 877,682 | 3∙2% (2∙9-3∙6) | 446,820 | Higher-quality estimates | Higher-quality estimates |
| **Slovakia** | 5,415,949 | 1∙1% (0∙7-1∙6) | 59,575 | 2∙0% (1∙4-2∙7) | 75,823 | Higher-quality estimates | Higher-quality estimates |
| **Slovenia** | 20,61,085 | 0∙087% (0∙043 – 0∙155) | 1,793 | 0∙016% (0∙002 – 0∙057) | 231 | Blood donor data | Blood donor data |
| **Spain** | 46,512,199 | 0∙8% (0∙6-1∙1) | 372,098 | 1∙1% (0∙3-2∙8) | 358,144 | Higher-quality estimates | Higher-quality estimates |
| **Sweden** | 9,644,864 | 0∙043% (0∙026 – 0∙065) | 4,147 | 0∙059% (0∙040 – 0∙085) | 3,983 | Blood donor data | Blood donor data |
| **United Kingdom** | 64,308,261 | 1∙7% (1∙3-2∙2) | 1,093,240 | 0∙9% (0∙7-1∙1) | 405,142 | Lower-quality estimates | Lower-quality estimates |
| ***Total*** | ***512,318,382*** | ***0∙9% (0∙7-1∙2)*** | ***4,700,068*** | ***1∙1% (0∙9-1∙4)*** | ***5,574,391*** | **-** | **-** |

* Eurostat 2014
** Back-calculated 95%CI from reported prevalence and sample size
*** No sample size reported
